# An Indole Dearomatization Strategy for the Synthesis of Pseudo‐Natural Products

**DOI:** 10.1002/cbic.202500182

**Published:** 2025-04-30

**Authors:** Joseph G. F. Hoock, Annina Burhop, Luca C. Greiner, Beate Schölermann, Celine Da Cruz Lopes Guita, Jie Liu, Sukdev Bag, Axel Pahl, Sonja Sievers, Rebecca Scheel, Carsten Strohmann, Slava Ziegler, Michael Grigalunas, Herbert Waldmann

**Affiliations:** ^1^ Department of Chemical Biology Max Planck Institute of Molecular Physiology 44227 Dortmund Germany; ^2^ Compound Management and Screening Center 44227 Dortmund Germany; ^3^ Faculty of Chemistry and Inorganic Chemistry TU Dortmund University 44227 Dortmund Germany; ^4^ Faculty of Chemistry Chemical Biology TU Dortmund University 44227 Dortmund Germany

**Keywords:** fused ring systems, reduction, heterocycles, morphological profiling, pseudo‐natural products

## Abstract

The indole moiety is a privileged fragment that frequently populates existing bioactive compound collections. The development of an indole‐dearomatization sequence and its application for library expansion of a collection of indole‐containing pseudo‐natural products (NPs) are described. The resulting compounds are topologically distinct from the original compound class. Phenotyping by means of the cell painting assay initially indicates that the dearomatized compounds are morphologically different than the original pseudo‐NP compound class and guiding NPs. However, analysis by means of a new subprofile analysis of the same cell painting assay data indicates that similar morphologies persist throughout the compound classes. Further biological studies support the findings of the subprofile analysis and highlight its potential to more effectively characterize novel compounds. The biological findings suggest that a plethora of indole‐dearomatization reactions can be applied to existing indole‐containing compound collections to rapidly access new biologically relevant scaffolds.

## Graphical abstract

An indole‐dearomatization/reduction strategy is developed to expand a collection of indole‐containing pseudo‐NPs. This approach enhances the three‐dimensionality of the original compound classes through hybridization‐changing conversions leading to topologically distinct novel structures. Morphological profiling reveals bioactivities that persist throughout the compound classes.

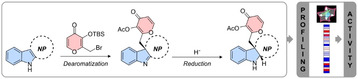

## 1 Introduction

Indole‐containing compounds are prevalent in natural products (NPs) and bioactive compounds.^[^ ^1^ ^]^ In addition to indoles being a privileged structure for bioactivity, their chemical reactivity makes them suitable substrates for library expansion into different areas of chemical and biological space.^[^ ^2^ ^]^ Of particular interest are dearomatization reactions which can introduce new stereocenters and significantly alter the 3D topology of the starting indole.^[^ ^3^, ^4^, ^5^ ^]^

**Figure 1:**
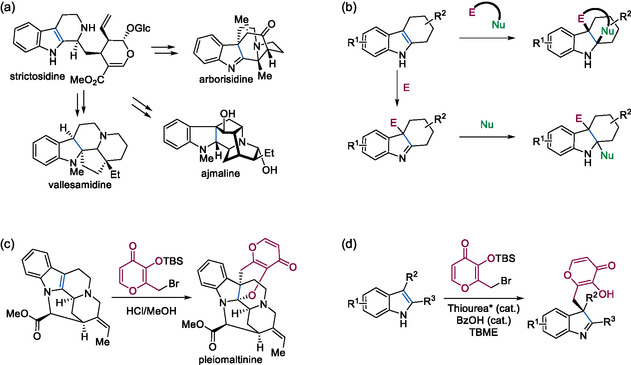
a) Divergent biosynthesis of indolenine and indoline monoterpene alkaloid NPs from the intermediate strictosidine via intramolecular indole dearomatizations. b) Synthetic dearomatization of indoles via annaulation or a step‐wise electrophilic and nucleophilic addition. E, electrophile; Nu, nucleophile. c) Indole‐dearomative annulation employing a γ‐pyrone derivative to complete the synthesis of the NP pleiomaltinine.^[^ ^19^ ^]^ d) Enantioselective indole‐dearomatization employing a γ‐pyrone derivative resulting in indolenines.^[^ ^20^ ^]^

This diversification strategy has been successfully exploited by Nature via biosynthetic pathways such as those employing strictosidine as a divergent intermediate (**Figure** **1**a).^[^ ^6^, ^7^ ^]^ While there are exceptions, biosynthetic dearomatizations are typically intramolecular leading to complex polycyclic scaffolds.^[^ ^8^, ^9^ ^]^ Synthetic chemistry offers a complementary approach to Nature's biosynthetic repertoire in that numerous divergent indole intermediates can be readily synthesized,^[^ ^10^, ^11^ ^]^ e.g., via Fischer indole or Pictet–Spengler reactions, and subjected to intermolecular dearomatization for the incorporation of new fragments to the core scaffold (Figure 1b).^[^ ^12^, ^13^ ^]^ Following this logic, we aimed to expand an indole‐containing pseudo‐NP collection via dearomatization.

The pseudo‐NP design principle combines NP fragments in arrangements that are not observed in Nature.^[^ ^14^, ^15^, ^16^, ^17^ ^]^ This leads to novel scaffolds that retain the biological relevance of NPs but are not obtainable through existing biosynthetic pathways. The intermolecular dearomatization of an indole‐containing pseudo‐NP collection could increase stereogenic content and three‐dimensionality of indole‐containing pseudo‐NPs while simultaneously installing an additional NP fragment. The resulting scaffolds may therefore be substantially different than the original compounds which may lead to new bioactivity profiles that would add further value to the overall compound collection. With this design principle in mind, we developed reaction conditions and explored the dearomatization of a collection of griseofulvin‐indole (GF‐indole) pseudo‐NPs. Cheminformatic analyses were used to show the shape diversity and NP‐likeness of the compound collection. Initial biological characterization of the collection via the cell painting assay (CPA) showed morphological diversity of the dearomatized products relative to their original counterparts; however, a recently developed subprofile analysis suggested that each pseudo‐NP class retained the native tubulin modulating bioactivity of the guiding NP GF.^[^ ^18^ ^]^ Further biological investigations confirmed this hypothesis and highlight the value of subprofile analyses in deconvoluting CPA results. Additionally, three other previously reported GF‐based pseudo‐NP collections were evaluated and did not retain the native tubulin modulating activity of the guiding NP GF, indicating that employing the pseudo‐NP concept to bioactive fragment‐sized NPs can in some cases but does not always change or abolish the bioactivity of the guiding NP.

## 2 Development of the Indole Dearomatization Method

Development of the dearomatization reaction was inspired by the work of Porco et al. in the synthesis of pleiomaltinine^[^ ^19^ ^]^ (Figure 1c) and the development of an enantioselective dearomatization by Jacobsen et al. (Figure 1d) in which the reaction of a quinone methide precursor with indole‐containing substrates is described.^[^ ^20^ ^]^ Depending on the indole employed, either an annulated indoline (Figure 1c) or a ring‐opened indolenine product (Figure 1d) is formed.

Investigation of dearomatization conditions was performed with spiro indolyl tetrahydropyran **1** with freshly generated maltol derivative **2** (Table S1). Initial attempts employed an excess of hydrochloric^[^ ^19^ ^]^ and led to formation of a compound in low yield, presumably **3a** which could not be isolated in pure form (**Table** **1**, entry 1). Product formation was improved when a mixture of MeOH/CH_2_Cl_2_ (3:1) without a Brønsted acid was employed (Table 1, entry 2); however, again, product instability circumvented isolation.

**Table 1:** Screening of reaction conditions for the dearomatization of **1**.

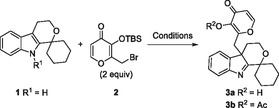				
Entry	Procedure	Product	Conditions	Yielda) [%]
1	A	**3a**	CH_3_CN/HCl in dioxane (1.5 equiv)	–b)
2	A	**3a**	MeOH/CH_2_Cl_2_ (3:1)	–b)
3	B	**3b**	MeOH/CH_2_Cl_2_ (3:1)	70
4	Bc)	**3b**	TFE/CH_2_Cl_2_ (3:1)	quant. (78)d)

Procedure A: **1** (1 equiv), **2** (2 equiv), solvent system, 22 °C, 15–60 min. Procedure B: 1) **1** (1 equiv), **2** (2 equiv), solvent system, 22 °C. 2) Ac_2_O (2.4 equiv), Et_3_N (3 equiv), DMAP (0.1 equiv), DCM, 22 °C, 30 min.

a) Yield determined by NMR;

b) Not isolable;

c) Reaction was conducted at 50 °C;

d) Isolated yield after GPC purification.

It was hypothesized that subsequent to the intermolecular dearomatization of the indole, cleavage of the OTBS group could occur, enhancing the reactivity of the enol group toward undesired side reactions. Despite various attempts, *N*‐acylation, ‐sulfonylation, and ‐carbamoylation of the suspected annulated product were unsuccessful. To circumvent the side reactions and enable product isolation, the in situ formed enol was trapped by acylation. To this end, after dearomatization of **1,** treatment of the mixture with acetic anhydride resulted in the exclusive formation of *O*‐acetylated product **3b** with a yield of ≈70% (Table 1, entry 3). Optimized conditions for formation of the ring‐opened product **3b** were finally found and consisted of treatment of **1** (1 equiv) and **2** (2 equiv) in TFE/CH_2_Cl_2_ (3:1) at 50 °C for 30 min followed by acetylation (Ac_2_O (2.4 equiv), Et_3_N (3 equiv), DMAP (0.1 equiv), and DCM, 22 °C, 30 min (Table 1, entry 4). The structure of **3b** was confirmed by X‐ray diffraction^[^ ^21^ ^]^ (Figure S1, Table S2, Supporting Information) and the compound was stable in a range of solvents and acidic media.

**Figure 2:**
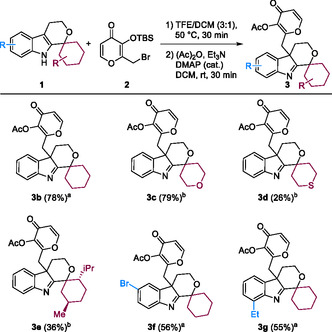
Substrate scope for dearomatization and subsequent *O*‐acetylation of spiro‐indole tetrahydropyrans. Isolated yield after ^a^GPC or ^b^silica chromatography purification.

Initial substrate scope exploration (**Figure** **2**) revealed that starting materials with spirocyclic cyclohexyl and *4 H*‐pyrano moieties gave dearomatized products in high yields (**3b,** X‐ray structure in SI, and **3c**, respectively) while a *4 H*‐thiopyrano and a sterically demanding menthone moiety led to lower yield (**3d** and **3e**, respectively). Reaction with bromo‐ and ethyl‐substituted indoles resulted in moderate yields of the desired dearomatized products **3f** and **3g**.

**Figure 3:**
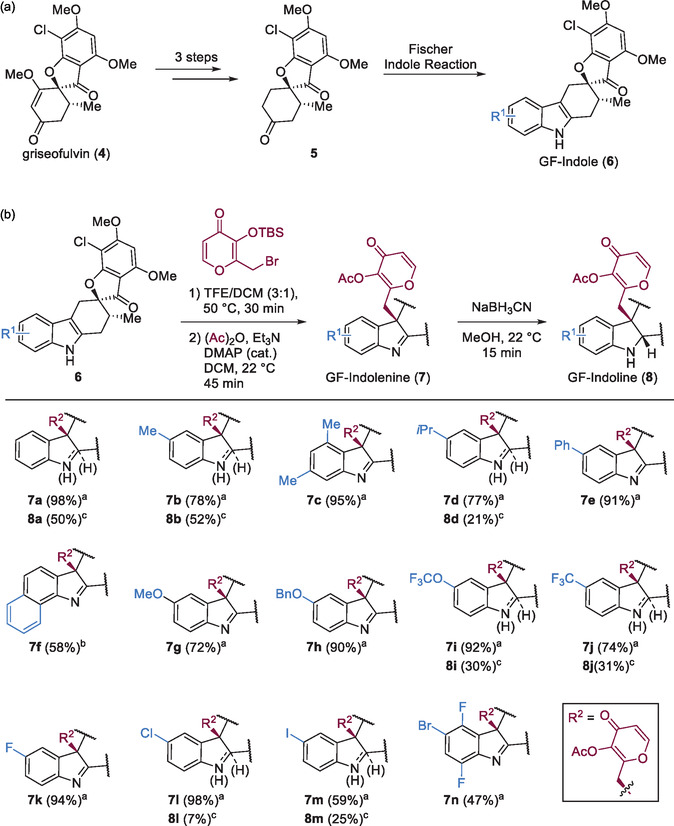
Synthesis of GF‐indole (**6**) intermediate and synthetic scope of GF‐indolenines (**7**) and GF‐indolines (**8**). a) Synthesis of GF‐indoles pseudo‐NPs in a four step reaction sequence. b) Substrate scope for the dearomatization, acetylation, and reduction sequence of GF‐indole pseudo‐NPs. Isolated yield over two steps after ^a^GPC or ^b^silica chromatography. ^c^Isolated yield over three steps after silica chromatography. The absolute stereochemistry of GF‐indolenine **7k** and GF‐Indoline **8j** was determined by X‐ray diffraction (Table S3)^[^ ^34^ ^]^ and NOESY NMR correlations, respectively, and other GF‐indolenines and GF‐indolines were assigned analogously (see Supporting Information for details).

The indole dearomatization was used to expand a collection of GF‐indole pseudo‐NPs. The scaffold of the GF‐indole compound was obtained by combination of the polyketide NP GF (**4**) and the alkaloid NP fragment indole by means of a four‐step reaction sequence (**Figure** **3**a).^[^ ^21^ ^]^

The dearomatization reaction performed well for all attempted reactions with GF‐indole substrates (**6**) resulting in good to excellent yields of *O*‐acetylated products (**7**) over two steps (Figure 3b). Various substituents on the indole ranging from alkyl residues, ethers, and halogens were tolerated. Additionally, the GF‐indolenine products can be reduced to the corresponding indolines with NaBH_3_CN in moderate yields over a three‐step sequence (Figure 3b). In total, 14 GF‐indolenine and 7 GF‐indoline pseudo‐NPs were synthesized. NMR stability tests (in CDCl_3_) indicated that **7f**, **7g**, and **7h** significantly decomposed after 4 days, whereas the other GF‐indolenines retained high purity after 1 month. Compounds **7f**–**7h** were therefore excluded from further analyses.

## 3 Cheminformatic Analysis

**Figure 4:**
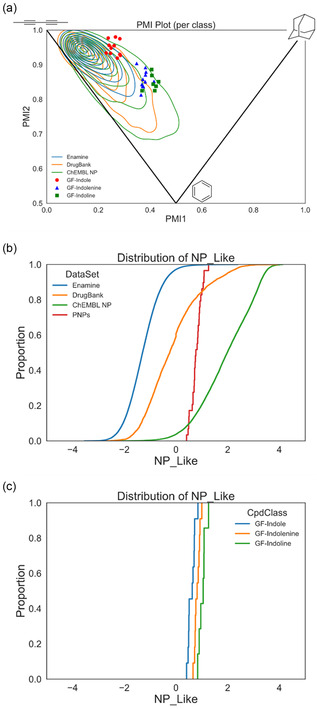
Cheminformatic analyses of the pseudo‐NPs reference sets. a) Principle moments of inertia plot showing the shape of the pseudo‐NP classes (GF‐indole (**6**, red circles), GF‐indolenine (**7**, blue triangles), and GF‐indoline (**8**, green squares)) and reference sets (Enamine (blue lines), DrugBank (orange lines), and ChEMBL NPs (green lines)). The corners of the triangle within the plot indicate a sphere‐like shape (top right), disk‐like shape (bottom middle), and rod‐like shape (top left). b) NP‐likeness score of the pseudo‐NPs (red) and reference sets (Enamine (blue), DrugBank (orange), and ChEMBL NPs (green)). c) NP‐likeness score of the individual pseudo‐NP classes (GF‐indole (**6**, blue), GF‐indolenine (**7**, orange), and GF‐indoline (**8**, green)). The NP‐likeness scores range from −5 (least NP‐like) to +5 (most NP‐like).

Cheminformatic analyses were employed to evaluate the differences between the GF‐indole starting materials, the new classes of dearomatized pseudo‐NPs, and reference datasets. The reference datasets are Enamine Advanced Screening Collection (representing a typical screening library), DrugBank compounds (representing approved and investigational drugs), and ChEMBL NPs (representing bioactive NPs). Characterization of shape by a principal moments of inertia analysis^[^ ^22^ ^]^ revealed that both the GF‐indolenines and the GF‐indolines are shifted away from the rod/disk‐like axis toward a more spherical shape relative to the GF‐indole starting materials and indicates that the dearomatized compounds have more 3D character (**Figure** **4**a). Relative to the majority of reference dataset compounds, the dearomatized pseudo‐NPs extend into a sparsely populated area of highly 3D shapes.

Compounds were evaluated by a NP‐likeness score^[^ ^23^ ^]^ in which structures with a more positive score are more NP‐like while those with a more negative score are less NP‐like. The GF‐derived pseudo‐NPs have a narrow distribution of NP‐likeness scores (+0.41 to 1.26) with the highest density of NP‐likeness overlapping where DrugBank compounds and ChEMBL NPs intersect (Figure 4b). Interestingly, through the pseudo‐NP synthetic sequence (i.e., GF‐indole → GF‐indolenine → GF‐indoline), the NP‐likeness score increases (Figure 4c).

## 4 Biological Evaluation via CPA

Biological evaluation and comparison of GF and GF‐derived pseudo‐NPs were done by the CPA.^[^ ^24^, ^25^ ^]^ The CPA is a morphological profiling method that quantifies phenotypic changes in cells upon compound treatment via staining of cellular compartments, fluorescence microscopy, and image analysis to extract 579 features that are condensed into a morphological profile. Activity in the CPA can be measured by the percentage of significantly changed features relative to DMSO controls known as an induction value.^[^ ^25^ ^]^ Individual profiles with similar induction values can be compared to other research compounds to evaluate performance diversity or to annotated reference compounds to facilitate mode of action or target hypotheses.

**Figure 5:**
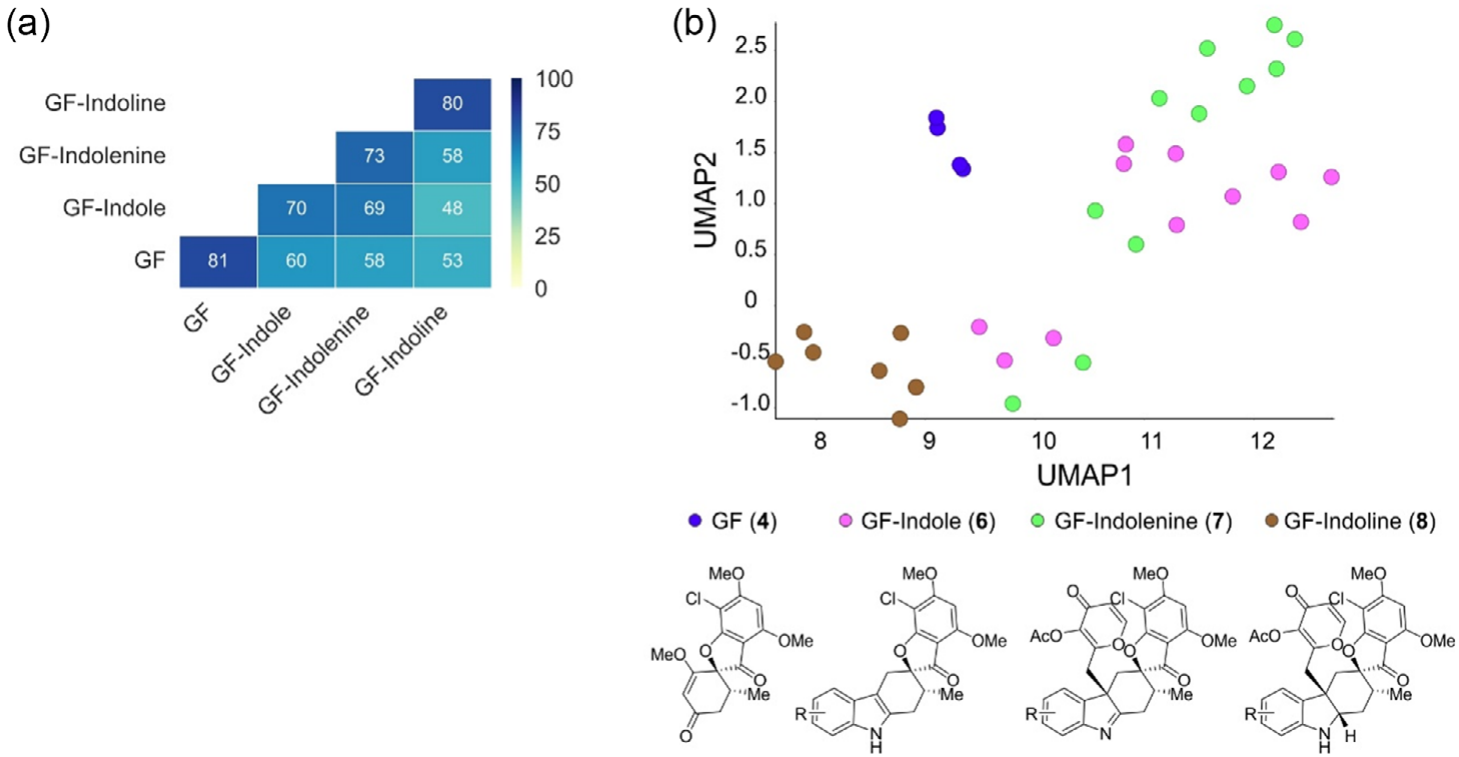
Morphological comparison of GF and GF‐derived pseudo‐NP classes via the CPA. a) Cross‐similarity analysis of median biosimilarity percentages within and between subclasses. Biosimilarity percentages ≥75% are considered similar. b) UMAP plot (7 neighbors) of GF (blue), GF‐indole (pink), GF‐indolenine (green), and GF‐indoline (brown) CPA profiles. Four biological replicates were used for GF in the analysis. Induction values of compounds are between 11% and 50%.

We initially analyzed the CPA dataset for the pseudo‐NP collection using a previously established workflow.^[^ ^21^, ^26^ ^]^ Several profiles, e.g., of entire compound classes, can be quantitatively compared through cross‐similarity analyses and calculation of median biosimilarity percentages (MBPs) in which MBPs ≥75% are considered similar. A morphological profile of each GF‐derived pseudo‐NP was selected with an induction between 11% and 50% and a relative cell count >50% for analysis. Similar induction ranges have been used for CPA analyses in previous studies and ensure that profile clustering is not influenced by the induction values.^[^ ^21^, ^26^ ^]^ Intraclass MBPs were calculated in which GF and GF‐indoline compounds were found to be similar (81% and 80%, respectively) whereas GF‐indole and GF‐indolenine classes were less homogeneous (70% and 73%, respectively) (**Figure** **5**). The fusion of an indole moiety to GF resulted in a significant morphological shift (GF compared to GF‐indole = 60% MBP). Dearomatization of GF‐indoles afforded compounds that were slightly below the similarity threshold of 75% (GF‐indole vs GF‐indolenine = 69%), while reduction of GF‐indolenines led to significantly different morphological profiles (GF‐indolenine vs GF‐indoline = 58%) (Figure 5). No classes were biosimilar to GF, which suggests, according to the CPA profiles, the pseudo‐NPs may not retain the native tubulin modulating activity of GF.^[^ ^27^ ^]^

As an alternative approach to cross‐similarity calculations, uniform manifold approximation and projection (UMAP) was employed as a tool for dimension reduction analysis to visualize clusters of compounds with similar profiles. The UMAP plot is in agreement with the cross‐similarity calculations, i.e., compound classes form distinguishable clusters, indicating similarities within compound classes and significant differences between compound classes. GF (blue) and GF‐indoline (brown) classes are significantly different than the others while GF‐indole (pink) and GF‐indolenine (green) form distinguishable clusters but are more expansive and have some overlap with each other. Overall, the biosimilarity and UMAP analyses suggest that the pseudo‐NP classes are morphologically different to each other and indicate that the pseudo‐NP collection does not have similarity to the parent NP GF which modulates tubulin and induces mitotic arrest.

For comparison, a second approach using a recently developed subprofile analysis was done.^[^ ^18^ ^]^ Briefly, a set of annotated compounds that have biosimilar profiles are identified and their shared significant features are extracted. The median of each feature is determined and features are combined to afford a median profile that is representative for a bioactivity cluster. Compounds that produce profiles with >80% similarity to a bioactivity cluster subprofile may suggest a potential mode of action without prior knowledge of the top biosimilar reference compounds. So far, 13 bioactivity cluster subprofiles have been defined which includes a profile for tubulin modulating compounds.^[^ ^18^, ^28^ ^]^

**Figure 6:**
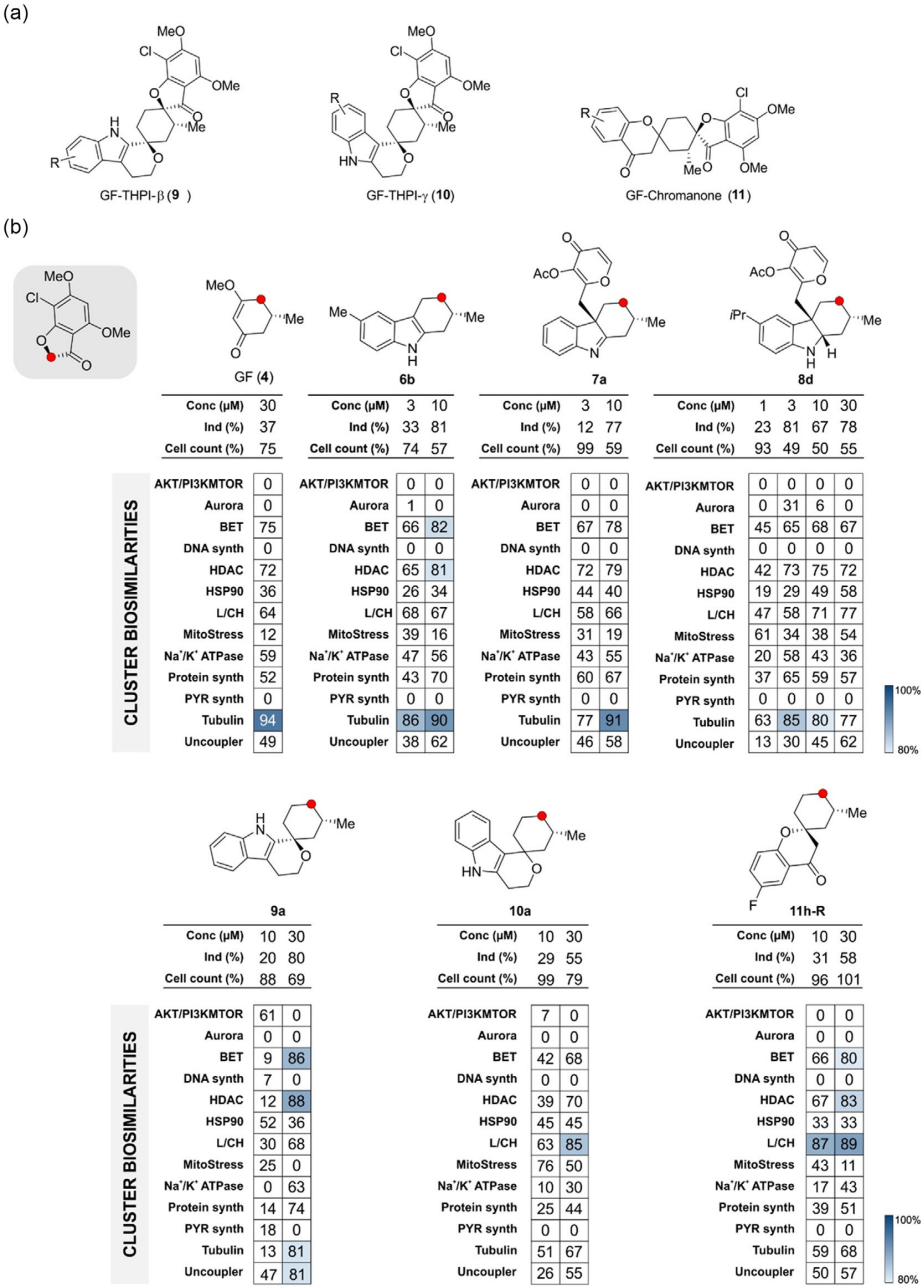
Similarities of selected compounds to cluster with different mode of action. a) Structure of compounds of GF‐derived compound classes. b) Similarities of selected compounds representative of each compound class to the 13 previously defined bioactivity clusters. Conc, concentration, Ind, induction; L/CH, lysosomotropism/cholesterol homeostasis; PYR, pyrimidine; synth, synthesis.

All profiles of compounds **6**‐**8** that have an induction between 10% and 85%, cell count ≥50%, and concentrations ≤30 μM were subjected to a subprofile analysis (**Figure** **6**, Table S4, Supporting Information). Additionally, other GF‐derived compound classes, i.e., GF‐THPI‐β (**9**), GF‐THPI‐γ (**10**), and GF‐Chromanone (**11**), were included for comparison (Figure 6a, Table S5, Supporting Information).^[^ ^21^, ^29^, ^30^ ^]^ Around half of compounds **6**–**8** induced profiles that were similar (≥80% biosimilarity) to the tubulin cluster subprofile. These findings are interesting as GF is one compound that was used to define the tubulin cluster (tubulin cluster subprofile similarity of 94% at 30 μM, Figure 6b). In light of these findings the conclusions of the initial CPA analysis that compound classes **6**–**8** neither have interclass similarities nor similarities to GF (Figure 5) were reconsidered. On the other hand, only one compound (**9a**) from **9** to **11** led to a profile with borderline similarity to the tubulin cluster subprofile (biosimilarity 81%). However, tubulin modulating compounds were not among the compounds with similar profiles. Therefore, the similarity to the tubulin cluster most likely stems from a cell death phenotype as recently reported.^[^ ^31^ ^]^ The cluster subprofile analysis of the GF‐THPI‐β class assigned compound and **9b**, **9d**, and **9e** as inhibitors of de novo pyrimidine biosynthesis, and this activity was previously confirmed.^[^ ^32^ ^]^ Moreover, for several THPI‐γ compounds, a similarity to the mitochondrial stress cluster was detected.^[^ ^28^ ^]^ This is in line with our earlier observations that compound **11c** impairs mitochondrial respiration and shares profile similar to oligomycin A.^[^ ^30^ ^]^

**Figure 7:**
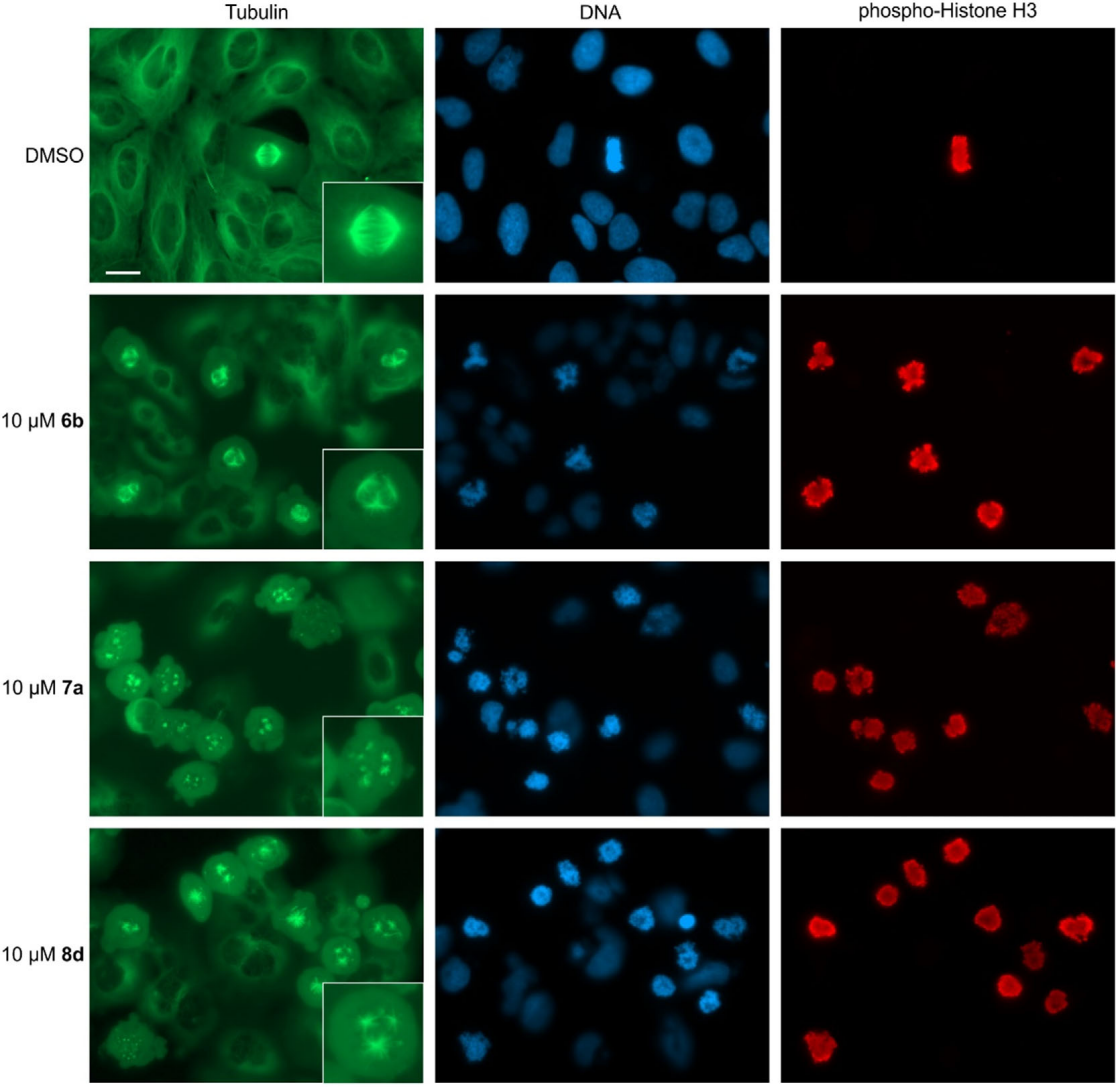
Influence of GF‐derived PNP on microtubules and mitosis. U2OS cells were treated with the compounds for 24 h prior to fixation and staining for tubulin, DNA and phospho‐Histone H3. Inlets show the morphology of representative mitotic spindles. Data are representative of *n* = 3. Scale bar: 20 μm.

For further evaluation of each GF‐derived pseudo‐NP compound class representative compounds were selected from each class that induces a profile with ≥80% biosimilarity to the tubulin cluster subprofile at the lowest concentration (Figure 6b). Compounds **6b**, **7a**, and **8d** induced profiles that are similar to the tubulin cluster subprofile at 3 and/or 10 μM, whereas a 30 μM concentration is needed for **9a** to reach 80% biosimilarity. The profiles of compounds **10a** and **11h‐R** did not display similarity to the tubulin cluster up to 30 μM. The ability of these six compounds to modulate tubulin polymerization was assessed in an in vitro tubulin polymerization assay. Only compound **8d** substantially inhibited tubulin polymerization (Figure S3, Supporting Information). As inhibition of tubulin polymerization arrests cells in mitosis, we visualized the mitotic marker phospho‐histone H3 along with DNA and tubulin in cells. GF **6b**, **7a**, and **8d** at 10 μM led to accumulation of cells in mitosis. Mitotic cells displayed impaired mitotic spindle architecture: **6b** led to formation of multipolar spindles, whereas for **7a** and **8 d** spindle formation is disturbed (**Figure** **7**). After treatment with **7a** or **8d**, multiple tubulin aggregation sites were observed from which only short, if at all, microtubules emanated (Figure 7). This is indicative of disturbed microtubule dynamics in cells. GF‐derived compounds **11a**, **10a**, and **11h‐R** did not have any effect on cell morphology and the tubulin cytoskeleton even at 30 μM (Figure S4, Supporting Information).

In conclusion, an indole‐dearomatization/acetylation and indolenine reduction sequence has been developed and applied for library expansion to a collection of indole‐containing pseudo‐NPs. Modulation of the tubulin cytoskeleton by GF was exceeded by coupling the fragment to indole, indolenine, and indoline. The resulting indolenines and indolines are topologically distinct from the original pseudo‐NP compound class. A range of indole‐dearomatization reactions are available and may be applied to existing indole‐containing compound collections to rapidly expand into new areas of chemical and biological space.^[^ ^21^, ^33^ ^]^

## Conflict of Interest

The authors declare no conflict of interest.

## Supporting information

Supplementary Material

## Data Availability

The data that support the findings of this study are available from the corresponding author upon reasonable request.
