# Supplementary material for: An Indole Dearomatization Strategy for the Synthesis of Pseudo‐Natural Products
Source: Chembiochem. 2025 Apr 30;26(10):e202500182. doi: 10.1002/cbic.202500182 (PMC12118328; doi:10.1002/cbic.202500182)
Supplement: Supplementary file 1 — Supplementary Material [file CBIC-26-e202500182-s001.zip › Supporting Information for Accepted Article_revised.pdf]

# Supporting Information

# An Indole Dearomatization Strategy for the Synthesis of Pseudo-Natural Products

# Contents

Supplementary Information Figures ........ 3
Experimental Details ........ 7
Cell Painting Assay ........ 7
Synthetic Experimental Details ........ 12
General Details ........ 12
Spiro-indole substrates **1** ........ 13
Optimization and Synthesis of Compound **2** ........ 17
Spiro-indolenine library members **3b**-**3g** ........ 20
Synthesis of Griseofulvin-Indolenines and Indolines (**7** and **8**) ........ 25
Synthesis of GF-THPI-$\beta$ (**9**), GF-THPI-$\gamma$ (**10**) and GF-Chromanones (**11**) ........ 39
Structural Determination of **8j** ........ 40
X-Ray Crystallography Data ........ 41
X-ray analysis of compound **3b** ........ 41
X-ray analysis of compound **7k** ........ 44
References ........ 47
NMR Spectra ........ 48

# Supplementary Information Figures

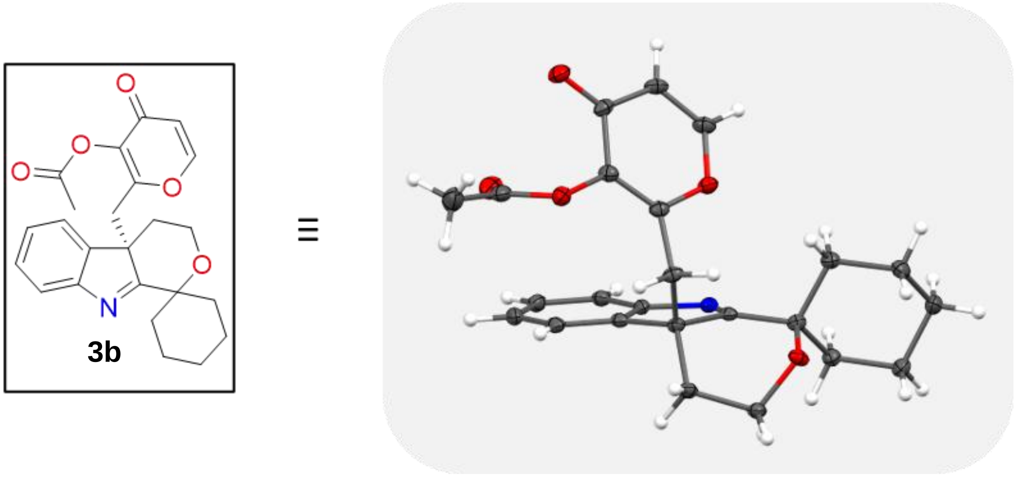


Figure S1: X-ray crystal structure of **3b**.

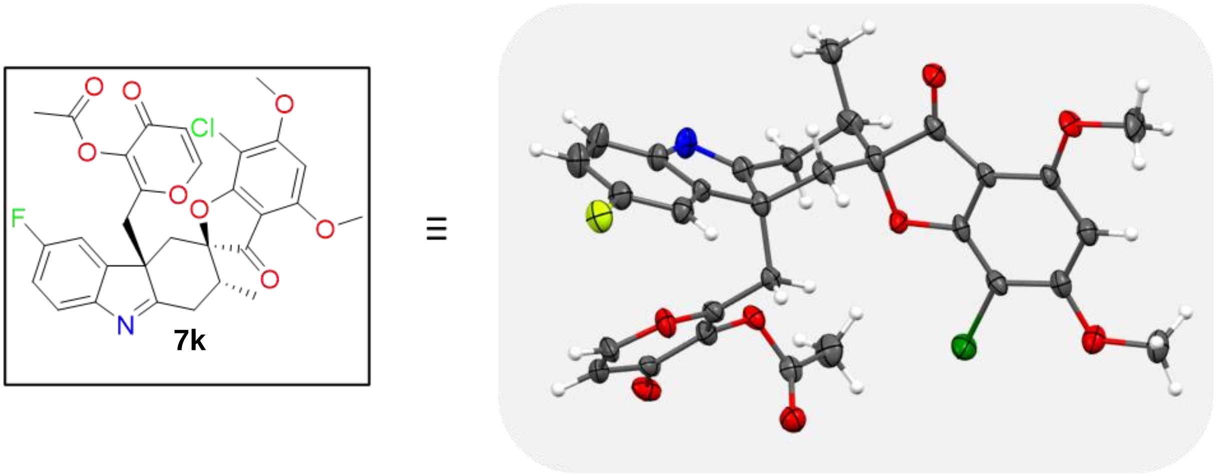


Figure S2: X-ray crystal structure **7k**. The newly generated stereocenter was determined to have the (*S*) configuration and was assumed to be analogous for similar products (**7**).

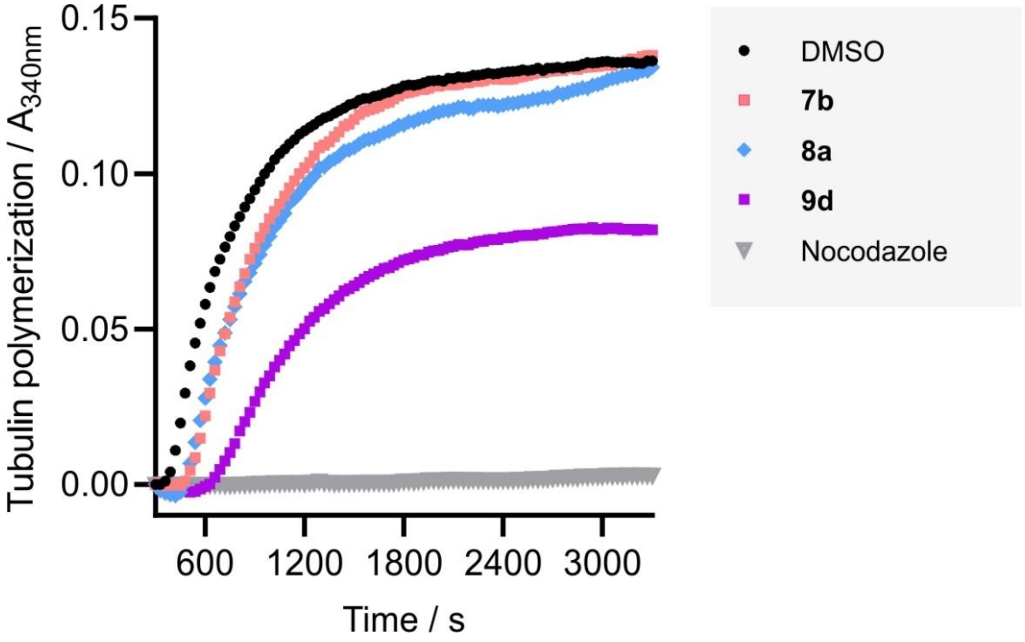


Figure S3: Influence of the compounds on *in vitro* tubulin polymerization. Compound concentration: 20 µM. Nocodazole (2 µM) was used as a control. Data are representative of n=4.

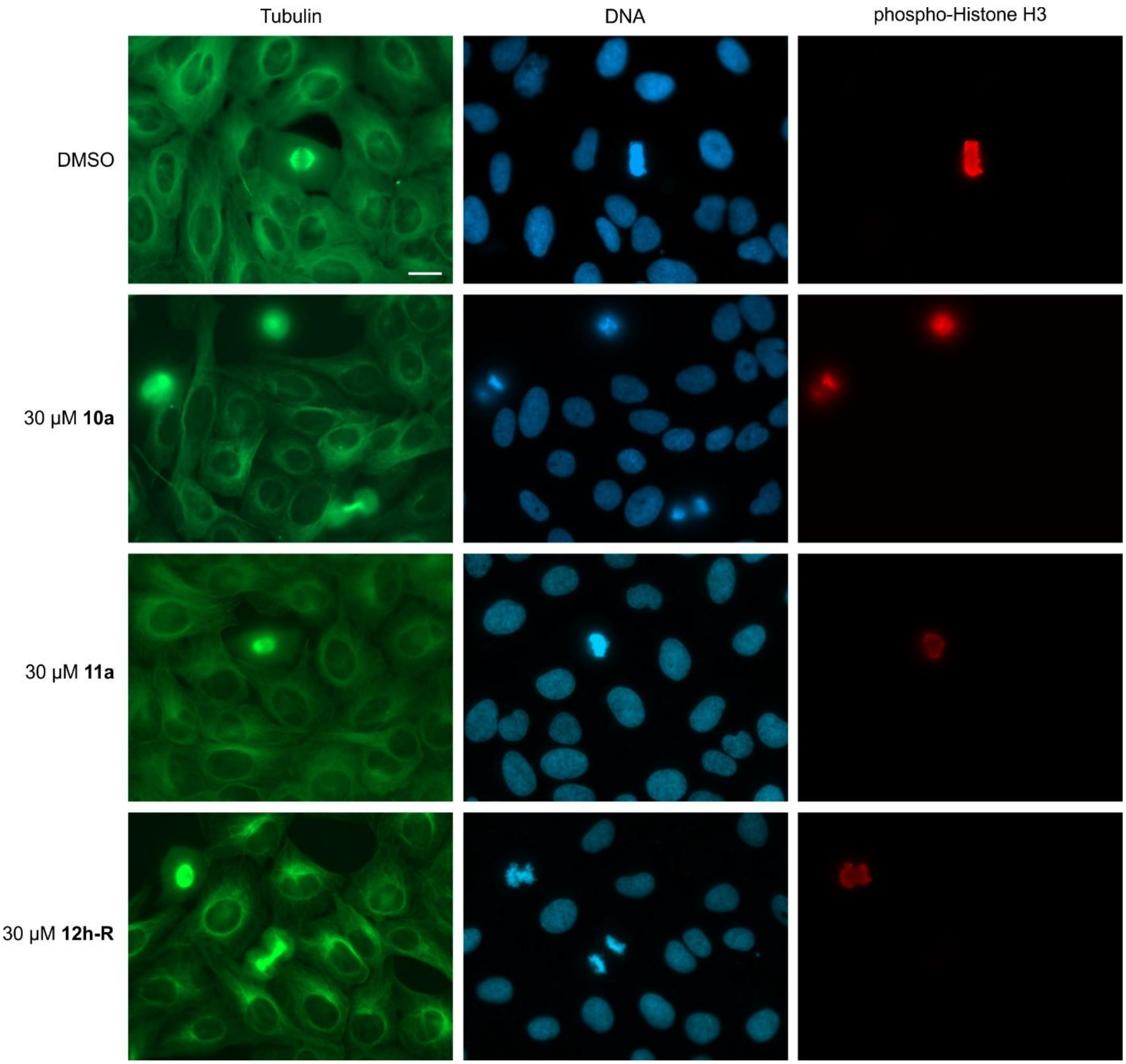


Figure S4: Influence of GF-derived compounds on microtubules and mitosis. U2OS cells were treated with the compounds for 24 h prior to fixation and staining for tubulin, DNA and phospho-Histone H3. Data representative of n=4. Scale bar: 20 μm.

# Experimental Details

## Cell Painting Assay

The described assay follows closely the method described by Bray et al.[1] Initially, 5 µl U2OS medium were added to each well of a 384-well plate (PerkinElmer CellCarrier-384 Ultra). Subsequently, U2OS cell were seeded with a density of 1600 cells per well in 20 µl medium. The plate was incubated for 10 min at the ambient temperature, followed by an additional 4 h incubation (37 °C, 5% CO2). Compound treatment was performed with the Echo 520 acoustic dispenser (Labcyte) at final concentrations of 10 µM, 3 µM or 1 µM. Incubation with compound was performed for 20 h (37 °C, 5% CO2). Subsequently, mitochondria were stained with Mito Tracker Deep Red (Thermo Fisher Scientific, Cat. No. M22426). The Mito Tracker Deep Red stock solution (1 mM) was diluted to a final concentration of 100 nM in prewarmed medium. The medium was removed from the plate leaving 10 µl residual volume and 25 µl of the Mito Tracker solution were added to each well. The plate was incubated for 30 min in darkness (37 °C, 5% CO2). To fix the cells 7 µl of 18.5 % formaldehyde in PBS were added, resulting in a final formaldehyde concentration of 3.7 %. Subsequently, the plate was incubated for another 20 min in darkness (RT) and washed three times with 70 µl of PBS. (Biotek Washer Elx405). Cells were permeabilized by addition of 25 µl 0.1% Triton X-100 to each well, followed by 15 min incubation (RT) in darkness. The cells were washed three times with PBS leaving a final volume of 10 µl. To each well 25 µl of a staining solution were added, which contains 1% BSA, 5 µl/ml Phalloidin (Alexa594 conjugate, Thermo Fisher Scientific, A12381), 25 µg/ml Concanavalin A (Alexa488 conjugate, Thermo Fisher Scientific, Cat. No. C11252), 5 µg/ml Hoechst 33342 (Sigma, Cat. No. B2261-25mg), 1.5 µg/ml WGA-Alexa594 conjugate (Thermo Fisher Scientific, Cat. No. W11262) and 1.5 µM SYTO 14 solution (Thermo Fisher Scientific, Cat. No. S7576). The plate is incubated for 30 min (RT) in darkness and washed three times with 70 µl PBS. After the final washing step, the PBS was not aspirated. The plates were sealed and centrifuged for 1 min at 500 rpm.

The plates were prepared in triplicates with shifted layouts to reduce plate effects and imaged using a Micro XL High-Content Screening System (Molecular Devices) in 5 channels (DAPI: Ex350-400/ Em410-480; FITC: Ex470-500/ Em510-540; Spectrum Gold: Ex520-545/ Em560-585; TxRed: Ex535-585/ Em600-650; Cy5: Ex605-650/ Em670-715) with 9 sites per well and 20x magnification (binning 2).

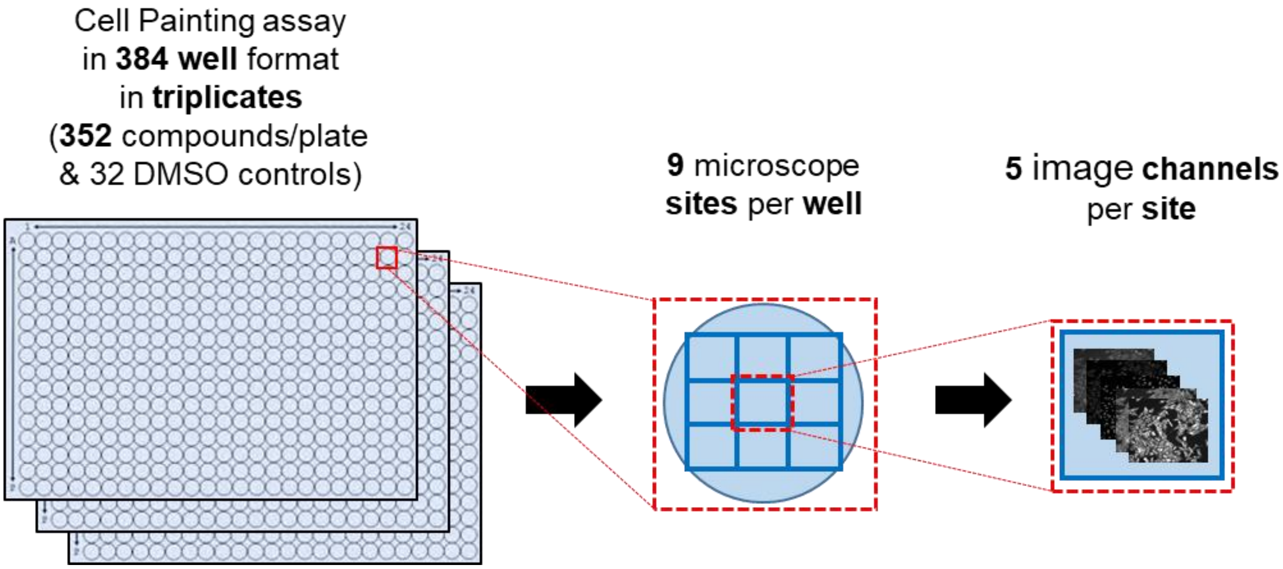


The generated images were processed with the *CellProfiler* package (https://cellprofiler.org/, version 3.0.0) on a computing cluster of the Max Planck Society to extract 1716 cell features per microscope site. The data was then further aggregated as medians per well (9 sites -> 1 well), then over the three replicates.

Further analysis was performed with custom *Python* (https://www.python.org/) scripts using the *Pandas* (https://pandas.pydata.org/) and *Dask* (https://dask.org/) data processing libraries as well as the *Scientific Python* (https://scipy.org/) package (separate publication to follow).

From the total set of 1716 features, a subset of highly reproducible and robust features was determined using the procedure described by Woehrmann et al.[2] in the following way: Two biological repeats of one plate containing reference compounds were analyzed. For every feature, its full profile over each whole plate was calculated. If the profiles from the two repeats showed a similarity >= 0.8 (see below), the feature was added to the set.

This procedure was only performed once and resulted in a set of 579 robust features out of the total of 1716 that was used for all further analyses.

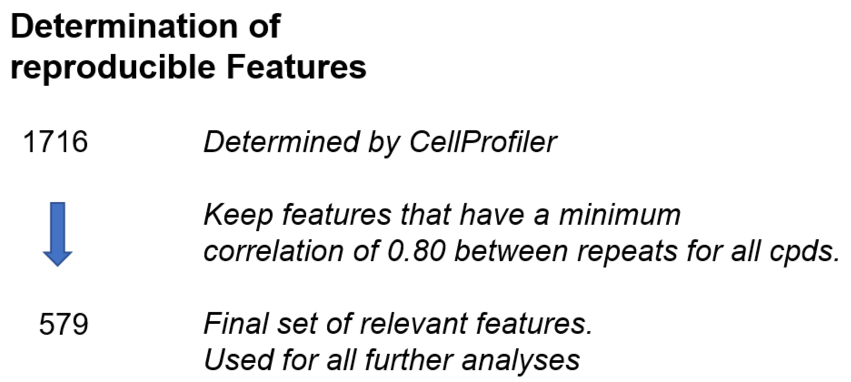


The phenotypic profiles were compiled from the Z-scores of all individual cellular features, where the Z-score is a measure of how far away a data point is from a median value.

Specifically, Z-scores of test compounds were calculated relative to the Median of DMSO controls. Thus, the Z-score of a test compound defines how many MADs (Median Absolute Deviations) the measured value is away from the Median of the controls as illustrated by the following formula:

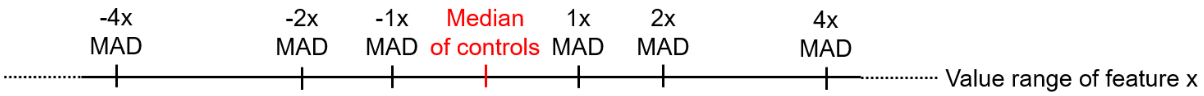


$$z\_score = \frac{value_{meas.} - Median_{Controls}}{MAD_{Controls}}$$

The phenotypic compound profile is then determined as the list of Z-scores of all features for one compound.

In addition to the phenotypic profile, an induction value was determined for each compound as the fraction of significantly changed features, in percent:

$$Induction\ [\%] = \frac{number\ of\ features\ with\ abs.\ values > 3}{total\ number\ of\ features}$$

Similarities of phenotypic profiles were calculated from the correlation distances between two profiles
(https://docs.scipy.org/doc/scipy/reference/generated/scipy.spatial.distance.correlation.html;

Similarity = 1 - Correlation Distance) and the compounds with the most similar profiles were determined from a set of 3000 reference compounds that was also measured in the assay.

An example for two compounds with highly similar profiles (96% similarity):

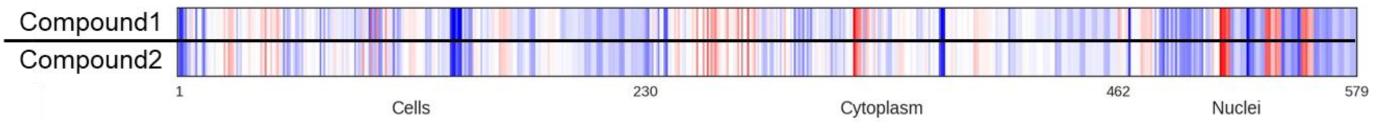


An example for two compounds with low similarity profiles (0% similarity):

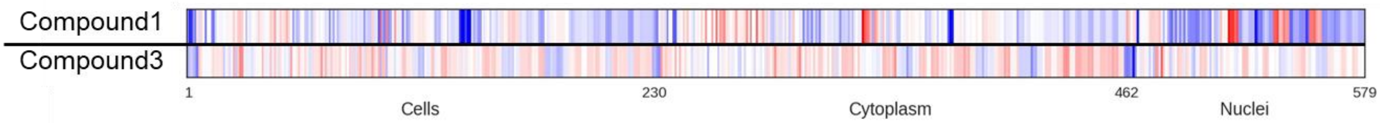


Each colored band represents one Z-score of a feature.

### *In vitro* tubulin polymerization assay

*In vitro* tubulin polymerization assay was performed as described previously by Pahl et al.[3] Porcine α/β-tubulin (Cytoskeleton, T240-B) was. Next, porcine α/β-tubulin (diluted in a general buffer containing 80 mM PIPES (pH 6.9), 2 mM $MgCl_2$ and 0.5 mM EGTA to final concentration 10 µM) was added to the wells of a 96-well plate to a solution containing $MgCl_2$ and glutamate (Sigma Aldrich, 49621-250G, final concentration of 0.88 µM and 0.8 mM, respectively). Subsequently, compounds were added to the tubulin solution to a final concentration of 20 µM. Samples were incubated at room temperature for 20 min prior to further incubation on ice for 20 min. Subsequently, GTP (Thermo Fisher, R0461) was added to a final concentration of 500 µM. Tubulin polymerization was monitored for 60 min by means of turbidity measurements at 340 nm using Infinite M200 plate reader (Tecan).

### Immunofluorescence

5,000 U2OS cells were seeded per well in a 96-well plate. After incubation overnight, cells were treated with the compounds or DMSO as a control for 24 hours. Cells were then fixed using 3.7 % paraformaldehyde in phosphate-buffered saline (PBS) and permeabilized with 0.1% Triton X-100 (in PBS). Cells were stained with DAPI to visualize DNA. Tubulin cytoskeleton was stained using anti-tubulin-FITC antibody (Sigma-Aldrich, Cat. No. F2168). Phospho-Histone H3 antibody

coupled to AF594 (Cell signaling, Cat. No. 8481) was used to detect mitotic cells. Images were acquired using Observer Z1 (Carl Zeiss, Germany) using 40 X objectives (LD Plan-Neofluar).

### Quantification and statistical analysis

Data are representative of independent (biological) replicates. n: number of biological replicates.

# Synthetic Experimental Details

## General Details

GF-Indoles (**6**) were prepared from Griseofulvin (**4**) according to Grigalunas et al.[4]

All reactions were performed in dried glassware under inert Argon atmosphere if not indicated differently. Dry solvents were purchased from Fischer Scientific and/or Acros and used without further treatment. Oxygen and/or moisture sensitive solutions were transferred using syringes and cannulas. Chemicals were purchased at their purest grades from Fischer Scientific and/or Acros and/or TLC Pharmaceutical Standards and/or Sigma Aldrich and/or Santa Cruz Biotechnology and/or Apollo Scientific. Solvents for HPLC analysis and purification were purchased at their purest grades from Fischer Scientific and/or Acros. TLC was performed on silica coated aluminium plates (Merck 60 $F_{254}$) and visualization was achieved under UV irradiation (254 nm), potassium permanganate stain (1.5 g $KMnO_4$, 10 g $K_2CO_3$, 1.25 mL of 10% aqueous NaOH solution and 200 mL of water) or *p*-anisaldehyde stain (0.7 mL *p*-anisaldehyde, 9.5 mL conc. $H_2SO_4$, 2.7 mL of acetic acid and 250 mL of EtOH). Analytical UHPLC-MS and LC-MS was performed on an Agilent 1290 Infinity system equipped with a mass detector (column: Zorbax Eclipse C18 Rapid Resolution 2.1x50 mm 1.8µm) and on a Thermo Scientific fleet station (column: Nuleodur C18 gravity EC 50/3, 1.8 µm). Flash column chromatography purification of crude products was achieved through FC, silica gel 60, 0.035-0.070 or medium pressure liquid chromatography (MPLC, Grace Reveleris X2) with Reveleris HP High Performance Silica Cartridges (No. 5170200 or 5170201) using the indicated solvents. Preparative HPLC purification was carried out on an Agilent 1100 system equipped with a mass detector (columns: Nuleodur C18 gravity VP 125/10 5 µm, Nuleodur C18 gravity VP 125/21 5 µm, Nuleodur C4 gravity VP 125/10 5 µm). Recycling gel permeation chromatography was performed on a Japan Analytical Industry LaboACE using JAIGEL 1-HH and 2-HH 20 mm x 600 mm, flowrate 7 ml/min, columns and $CHCl_3$ as solvent. NMR spectra were recorded on Bruker AV 400 Avance III HD (NanoBay), Agilent Technologies DD2, Bruker AV 500 Avance III HD (Prodigy), Bruker AV 600 Avance III HD (CryoProbe) or Bruker AV 700 Avance III HD (CryoProbe) spectrometers. Data is reported in ppm with reference to the used deuterated solvent ($CDCl_3$: 7.26 ppm, 77.16 ppm; d6-DMSO: 2.50 ppm, 39.52 ppm; MeOD: 3.31 ppm; 49.00 ppm). Signals were assigned to their corresponding Hydrogens or Carbons based on 2D-NMR correlations ($^1H/^1H$ COSY, $^1H/^1H$ NOESY, $^1H/^{13}C$ HSQC, $^1H/^{13}C$ HMBC). High-resolution mass spectrometry (HRMS) was performed on a TSQ

(Thermo Quest) quadrupole mass spectrometer. Microwave reactions were carried out in a CEM Discover SP Activent machine.

## Spiro-indole substrates 1

General procedure for the synthesis of spiro-indoles (**1**) via the oxa-Pictet Spengler reaction:[5]

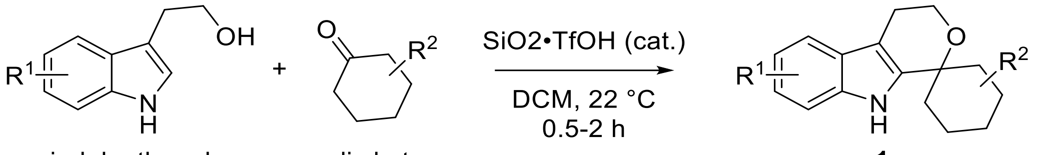


An indoleethanol (1.0 eq.) was added to an oven-dried microwave vial and dissolved in dry DCM (5 ml). $SiO_2$·TfOH and a cyclic ketone (1.5 eq.) was added. After flushing of the reaction tube with argon, the reaction mixture was stirred at rt for 0.5 – 2 h. The reaction was stopped by filtration with ethyl acetate (EtOAc) as indicated by TLC and LCMS analysis. The remaining solvent was evaporated to dryness. Afterwards, the crude reaction mixture was purified by means of silica flash column chromatography (CH/EtOAc 10-35% v/v) to isolate the title compound (**1**).

4',9'-dihydro-3'H-spiro[cyclohexane-1,1'-pyrano[3,4-b]indole] (**1a**)

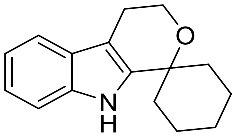


**1a**

White amorph solid (1.43 g, 96%); $\mathbf{R_f}$ = 0.15 (CH/EtOAc 10% v/v); **$^1$H NMR** (400 MHz, Chloroform-*d*) *δ* 7.50 (ddt, *J* = 7.5, 1.4, 0.6 Hz, 1H), 7.33 (ddd, *J* = 7.9, 1.2, 0.7 Hz, 1H), 7.13 (m, 2H), 4.02 (d, *J* = 5.5 Hz, 2H), 2.80 (t, *J* = 5.5 Hz, 2H), 2.05 (m, 2H), 1.79 (m, 3H), 1.62 (m, 4H), 1.30 (m, 1H) ppm. **$^{13}$C NMR** (126 MHz, Chloroform-*d*) *δ* 139.7, 135.7, 127.2, 121.7, 119.7, 118.4, 110.9, 107.0, 72.6, 59.8, 35.9, 25.6, 22.6, 21.5 ppm. **HR-MS (ESI)** *m/z* calculated for $C_{16}H_{20}NO$ $[M+H]^+$: 242.1539, found 242.1539.

9'-methyl-4',9'-dihydro-3'H-spiro[cyclohexane-1,1'-pyrano[3,4-b]indole] (**1b**)

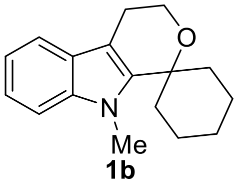

**1b**

White crystalline solid (228 mg, 76%); $^{1}$**H NMR** (500 MHz, Chloroform-*d*) δ 7.50 (dd, *J* = 7.8, 1.2 Hz, 1H), 7.27 (d, *J* = 8.2 Hz, 1H), 7.20 (ddd, *J* = 8.3, 6.9, 1.2 Hz, 1H), 7.11 (m, 1H), 3.95 (t, *J* = 5.5 Hz, 2H), 3.78 (s, 3H), 2.82 (t, *J* = 5.4 Hz, 2H), 1.99 (dq, *J* = 13.4, 2.2 Hz, 2H), 1.85 (m, 5H), 1.62 (m, 2H), 1.29 (dtd, *J* = 16.3, 13.6, 12.8, 4.0 Hz, 1H) ppm. $^{13}$**C NMR** (126 MHz, Chloroform-*d*) δ 140.0, 137.5, 126.4, 121.5, 119.2, 118.2, 108.9, 107.6, 73.6, 59.3, 34.2, 31.8, 27.0, 25.6, 23.0, 21.4 ppm. **HR-MS (ESI)** *m/z* calculated for $C_{17}H_{22}NO$ $[M+H]^+$: 256.1696, found 256.1696.

9'-methyl-4',9'-dihydro-3'H-spiro[cyclohexane-1,1'-pyrano[3,4-b]indole] (1**c**)

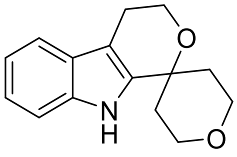

**1c**

White amorph solid (108 mg, 72%); $\mathbf{R_f}$ = 0.13 (CH/EtOAc 10% v/v); $^{1}$**H NMR** (700 MHz, Chloroform-*d*) δ 7.78 (s, 1H), 7.50 (d, *J* = 7.8 Hz, 1H), 7.35 (d, *J* = 8.0 Hz, 1H), 7.18 (td, *J* = 7.5, 6.9, 1.2 Hz, 1H), 7.12 (t, *J* = 7.4 Hz, 1H), 4.02 (t, *J* = 5.4 Hz, 2H), 3.93 (td, *J* = 11.9, 1.9 Hz, 2H), 3.88 (dd, *J* = 11.5, 5.1 Hz, 2H), 2.82 (t, *J* = 5.4 Hz, 2H), 2.04 (td, *J* = 13.8, 12.9, 5.3 Hz, 2H), 1.90 (d, *J* = 13.8 Hz, 2H) ppm. $^{13}$**C NMR** (176 MHz, Chloroform-*d*) δ 137.8, 135.9, 127.1, 122.2, 119.9, 118.5, 111.1, 107.9, 70.2, 63.5, 60.2, 36.0, 22.6 ppm. **HR-MS (ESI)** *m/z* calculated for $C_{15}H_{18}NO_2$ $[M+H]^+$: 244.1332, found 244.1333.

2',3',4,5',6',9-hexahydro-3H-spiro[pyrano[3,4-b]indole-1,4'-thiopyran] (**1d**)

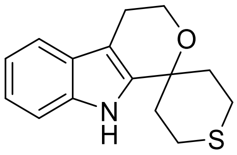

**1d**

White porous solid (138 mg, 85%); **$R_f$** = 0.4 (CH/EtOAc 10% v/v); **$^1$H NMR** (700 MHz, Chloroform-*d*) $\delta$ 7.68 (s, 1H), 7.50 (d, *J* = 7.8 Hz, 1H), 7.34 (d, *J* = 8.1 Hz, 1H), 7.18 (t, *J* = 7.6 Hz, 1H), 7.12 (t, *J* = 7.4 Hz, 1H), 3.99 (t, *J* = 5.4 Hz, 2H), 3.21 (m, 2H), 2.80 (t, *J* = 5.4 Hz, 2H), 2.44 (dt, *J* = 15.7, 3.4 Hz, 2H), 2.29 (d, *J* = 14.1 Hz, 2H), 2.01 (td, *J* = 13.5, 3.6 Hz, 2H) ppm. **$^{13}$C NMR** (176 MHz, Chloroform-*d*) $\delta$ 138.6, 135.8, 127.1, 119.9, 118.5, 111.1, 107.3, 71.0, 59.8, 36.7, 31.1, 23.7, 22.5 ppm. **HR-MS (ESI)** *m/z* calculated for $C_{15}H_{18}NOS$ $[M+H]^+$: 260.1104, found 260.1106.

(2S,5R)-2-isopropyl-5-methyl-4',9'-dihydro-3'H-spiro[cyclohexane-1,1'-pyrano[3,4-b]indole] (**1e**)

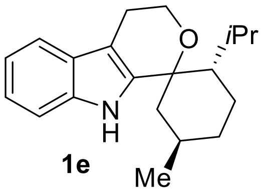

**1e**

White Solid (134 mg, 72%); **$R_f$** = 0.73 (CH/EtOAc 10% v/v); **$^1$H NMR** (400 MHz, Chloroform-*d*) $\delta$ 7.61 (s, 1H), 7.50 (ddt, *J* = 7.4, 1.5, 0.7 Hz, 1H), 7.33 (ddd, *J* = 7.9, 1.3, 0.8 Hz, 1H), 7.14 (m, 2H), 4.09 (ddd, *J* = 11.2, 5.7, 1.1 Hz, 1H), 3.86 (td, *J* = 11.3, 3.4 Hz, 1H), 2.91 (ddd, *J* = 15.1, 11.3, 5.7 Hz, 1H), 2.64 (ddd, *J* = 15.1, 3.4, 1.1 Hz, 1H), 2.23 (m, 1H), 1.87 (dtd, *J* = 12.0, 3.6, 2.1 Hz, 2H), 1.71 (m, 1H), 1.59 (dq, *J* = 13.2, 3.4 Hz, 1H), 1.48 (m, 2H), 1.12 (dd, *J* = 14.0, 11.9 Hz, 1H), 1.01 (m, 2H), 0.94 (dd, *J* = 7.8, 6.6 Hz, 1H), 0.90 (d, *J* = 6.5 Hz, 3H), 0.86 (m, 3H), 0.74 (d, *J* = 7.0 Hz, 3H) ppm. **$^{13}$C NMR** (101 MHz, Chloroform-*d*) $\delta$ 138.8, 135.8, 127.3, 121.5, 119.6, 118.2, 111.0, 108.7, 77.5, 59.8, 51.0, 44.2, 35.4, 27.8, 27.7, 24.0, 22.6, 22.4, 20.9, 18.9 ppm. **HR-MS (ESI)** *m/z* calculated for $C_{20}H_{28}NO$ $[M+H]^+$: 298.2165, found 298.2168.

6'-bromo-4',9'-dihydro-3'H-spiro[cyclohexane-1,1'-pyrano[3,4-b]indole] (**1f**)

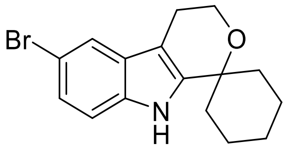

**1f**

White Solid (54 mg, 81%); $\mathbf{R_f}$ = 0.37 (CH/EtOAc 10% v/v); **$^1$H NMR** (400 MHz, Chloroform-*d*) δ 7.70 (s, 1H), 7.61 (dt, *J* = 1.9, 0.6 Hz, 1H), 7.23 (dd, *J* = 8.6, 1.9 Hz, 1H), 7.18 (dd, *J* = 8.6, 0.6 Hz, 1H), 3.99 (t, *J* = 5.5 Hz, 2H), 2.74 (t, *J* = 5.5 Hz, 2H), 2.04 (m, 2H), 1.77 (dddd, *J* = 14.9, 7.1, 3.9, 1.9 Hz, 3H), 1.62 (m, 4H), 1.29 (m, 1H) ppm. **HR-MS (ESI)** *m/z* calculated for $C_{16}H_{19}BrNO$ $[M+H]^+$: 320.0645, found 320.0650.

8'-ethyl-4',9'-dihydro-3'H-spiro[cyclohexane-1,1'-pyrano[3,4-b]indole] (**1g**)

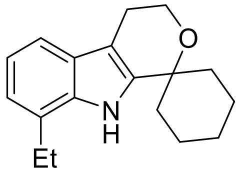

**1g**

White solid (66 mg, 93%); $\mathbf{R_f}$ = 0.5 (CH/EtOAc 10% v/v); **$^1$H NMR** (400 MHz, Chloroform-*d*) δ 7.52 (s, 1H), 7.36 (dd, *J* = 7.7, 1.2 Hz, 1H), 7.08 (m, 1H), 7.01 (d, *J* = 7.2 Hz, 1H), 4.01 (t, *J* = 5.4 Hz, 2H), 2.87 (q, *J* = 7.6 Hz, 2H), 2.80 (t, *J* = 5.4 Hz, 2H), 2.06 (m, 2H), 1.80 (td, *J* = 10.2, 3.9 Hz, 3H), 1.66 (m, 4H), 1.38 (t, *J* = 7.6 Hz, 3H), 1.33 (m, 1H) ppm. **$^{13}$C NMR** (101 MHz Chloroform-*d*) δ 139.3, 134.4, 127.0, 126.3, 120.4, 120.0, 116.1, 107.6, 72.6, 59.9, 35.9, 27.1, 25.6, 24.2, 22.7, 21.5, 14.0 ppm. **HR-MS (ESI)** *m/z* calculated for $C_{18}H_{24}NO$ $[M+H]^+$: 270.1852, found 270.1853.

## Optimization and Synthesis of Compound 2

Table S1: Investigation of different reaction conditions in order to replace carbon tetrachloride ($CCl_4$) in the second reaction step of the synthesis of the reactive maltol species.

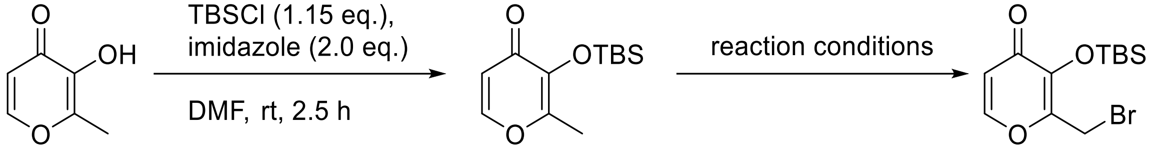


| Entry | Reagents | Solvent | *T* [°C] | *t* [min] | Yield* [%] |
|---|---|---|---|---|---|
| 1 | NBS (1.05 eq.), AIBN (0.13 eq.) | $CCl_4$ | 105 | 60 | 32 |
| 2 | NBS (1.05 eq.), AIBN (0.13 eq.) | $CHCl_3$ | 85 | 60 | 7 |
| 3 | NBS (1.05 eq.), AIBN (0.13 eq.) | DCE | 105 | 60 | 5[a] |
| 4 | NBS (1.05 eq.), AIBN (0.13 eq.) | DMC | 105 | 120 | 15[a] |
| 5 | DBDMH (1.05 eq.), AIBN (0.13 eq.) | DMC | 105 | 120 | - |
| 6 | NBS (1.05 eq.), AIBN (0.13 eq.) | DMC | 105 | 60 | 66[b] |
| 7 | NBS (1.05 eq.), AIBN (0.13 eq.), mw | DMC | 105 | 1 | 75[b] |

*Yield determined by NMR. After isolation [a] via silica flash chromatography or [b] aqueous work up.

3-((tert-butyldimethylsilyl)oxy)-2-methyl-4H-pyran-4-one

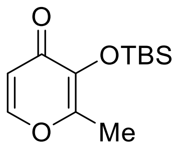


An oven-dried 500 ml round-bottom flask was charged with maltol (5.08 g, 40.28 mmol, 1.0 eq.) and anhydrous DMF (80 ml) was added under an argon atmosphere. After the addition of TBSCl (7.10 g, 47.13 mmol, 1.17 eq.), the resulting reaction mixture was stirred for 5 min. Then imidazole

was added (5.48 g, 80.56 mmol, 2.0 eq) and the solution was stirred at rt for 2.5 h. Since the reaction was finished as indicated by TLC, the reaction was quenched by the addition of sodium hydrogen carbonate ($NaHCO_3$,120 ml). After the separation of the layers, the aqueous phase was extracted with cyclohexane (3 x 50 ml) and the combined organic phases were subsequently dried over sodium sulfate ($NaSO_4$). The resulting slurry was filtrated and the remaining solvent was evaporated to dryness which gave the title compound as white crystals (9.54 g, 99% yield). $\mathbf{R_f}$ = 0.53 (CH/EtOAc 30% v/v). **$^1$H NMR** (400 MHz, Chloroform-*d*) $\delta$ 7.57 (dt, $J$ = 5.6, 1.5 Hz, 1H), 6.29 (ddd, $^{3/4}J$ = 5.6, 2.2, 1.3 Hz, 1H), 2.31 (s, 3H), 0.96 (s, 9H), 0.26 (s, 6H). **$^{13}$C NMR** (100 MHz, Chloroform-*d*) $\delta$ 174.2, 154.7, 151.9, 142.0, 115.3, 25.9, 18.7, 14.7, 3.8 ppm. **HR-MS (ESI)** m/z calculated for $C_{12}H_{21}O_3Si$ $[M+H]^+$: 241.1254, found 241.1252. All spectral data were in agreement with reported values.[6]

2-(bromomethyl)-3-((tert-butyldimethylsilyl)oxy)-4H-pyran-4-one (**2**)

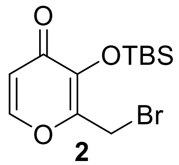


**2**

An oven dried high pressure flask charged with 3-((tert-butyldimethylsilyl)oxy)-2-methyl-4H-pyran-4-one (150 mg, 0.62 mmol, 1.0 eq.). Then DMC (3 ml), NBS (116.62 mg, 0.66 mmol, 1.05 eq.) and AIBN (13.32 mg, 0.08 mmol, 0.13 eq.) were subsequently added. The resulting suspension was heated to 105 °C for 1min in a microwave irradiator under an argon atmosphere. Then the reaction mixture was cooled to rt and quenched with $NaHCO_3$ (5 ml). After separation of the layers, the aqueous layer was extracted with cyclohexane (3 x 3 ml) and the combined organic phases were dried over $NaSO_4$. The remaining solvent was evaporated to dryness and the crude reaction mixture was stored under reduced pressure before being subjected to the next reaction. **$^1$H NMR** (400 MHz, Chloroform-*d*) $\delta$ 7.66 (d, $J$ = 5.6 Hz, 1H), 6.33 (d, $J$ = 5.6 Hz, 1H), 4.43 (s, 2H), 0.99 (s, 9H), 0.31 (s, 6H) ppm. **$^{13}$C NMR** (176 MHz, Chloroform-*d*) $\delta$ ppm: 174.5, 153.5, 151.2, 143.5, 115.9, 25.8, 23.2, 18.9, -3.4 ppm. **HR-MS (ESI)** m/z calculated for $C_{12}H_{20}BrO_3Si$ $[M+H]^+$: 319.0360, found 319.0365. All spectral data were in agreement with reported values.[6]

## Spiro-indolenine library members 3b-3g

General two-step procedure to generate library compounds **3b**-**3g**

Spiro-Indole (1.0 eq.) was added to an argon flushed round-bottom flask and dissolved in dry DCM (1 M). Afterwards, 2-(bromomethyl)-3-((tert-butyldimethylsilyl)oxy)-4H-pyran-4-one **2** (2.0 eq.) was dissolved in TFE (0.32 M) and added to the stirring solution. The reaction mixture was heated to 50 °C and continuously stirred for 30 minutes. After complete conversion of the starting material as indicated by TLC and LC-MS analysis, the solvent was evaporated to dryness and the crude was directly used in the next reaction (100% conversion of the starting material assumed).

Crude spiro-indolenine (1.0 eq.) was dissolved in dry DCM (2 ml) under argon atmosphere. Then, catalytic amounts of DMAP (0.1 eq.) and $Et_3N$ (3.0 eq.) were added and the brown reaction mixture was stirred for 5 minutes at 0 °C. Following, $(Ac)_2O$ (2.4 eq.) was added and the dark brown reaction mixture was continuously stirred at rt. After 30 minutes, the starting material was completely converted into the desired product as indicated by TLC and LC-MS analysis. The reaction was quenched by addition of $NaHCO_3$ (5 ml) and the aqueous layer was subsequently extracted with EtOAc (3 x 5 ml). The combined organic layers were dried under $NaSO_4$ and evaporated to dryness. The resulting crude was purified by either silica flash column chromatography (CH/EtOAc 100% v/v) or gel perfusion chromatography ($CHCl_3$) to isolate the title compound.

(*S*)-2-((3',4'-dihydro-4a'H-spiro[cyclohexane-1,1'-pyrano[3,4-b]indol]-4a'-yl)methyl)-4-oxo-4H-pyran-3-yl acetate (**3b**)

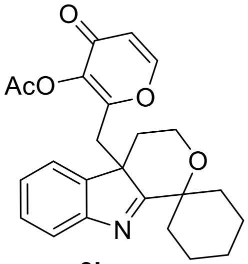

**3b**

White amorph solid (78%). **$^{1}$H NMR** (700 MHz, Chloroform-*d*) $\delta$ 7.56 (d, $J$ = 7.7 Hz, 1H), 7.37 (d, $J$ = 5.6 Hz, 1H), 7.30 (t, $J$ = 7.3 Hz, 1H), 7.14 (q, $J$ = 7.3 Hz, 2H), 6.22 (d, $J$ = 5.6 Hz, 1H), 4.09 (dt, $J$ = 11.9, 8.0 Hz, 1H), 3.80 (ddd, $J$ = 11.7, 8.1, 3.2 Hz, 1H), 3.52 (d, $J$ = 15.1 Hz, 1H), 3.38 (d, $J$ = 15.0 Hz, 1H), 2.57 (ddd, $J$ = 13.2, 7.3, 3.2 Hz, 1H), 2.32 (s, 3H), 2.18 (d, $J$ = 13.4 Hz, 1H), 2.05 – 1.95 (m, 2H), 1.88 (td, $J$ = 13.2, 3.7 Hz, 1H), 1.79 – 1.68 (m, 2H), 1.68 – 1.53 (m, 4H), 1.43 – 1.35 (m, 1H) ppm. **$^{13}$C NMR** (176 MHz, Chloroform-*d*) $\delta$ 189.8, 171.5, 167.3, 158.1, 153.8, 141.3, 139.3, 128.7, 125.8, 122.3, 120.9, 116.9, 78.0, 56.3, 55.2, 34.7, 34.0, 33.3, 33.2, 25.4, 25.3, 21.4, 21.2, 20.5 ppm. **HRMS**-ESI (*m/z*): [M + H]$^+$ calculated for $C_{24}H_{26}NO_5$ [M+H]$^+$ = 408.1806, found 408.1802.

(*S*)-2-((2,3,3',4',5,6-hexahydro-4a'*H*-spiro[pyran-4,1'-pyrano[3,4-b]indol]-4a'-yl)methyl)-4-oxo-4H-pyran-3-yl acetate (**3c**)

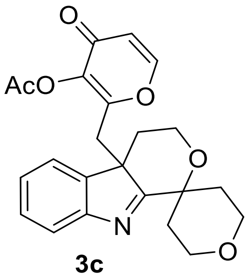

**3c**

Yellow solid (79%). **$^{1}$H NMR** (700 MHz, Chloroform-*d*) $\delta$ 7.58 (s, 1H), 7.36 (s, 1H), 7.32 (s, 1H), 7.17 (d, $J$ = 16.8 Hz, 2H), 6.23 (s, 1H), 4.11 (q, $J$ = 7.3 Hz, 1H), 3.94 (dt, $J$ = 11.3, 3.7 Hz, 1H), 3.89 (td, $J$ = 11.1, 3.1 Hz, 1H), 3.83 (m, 3H), 3.47 (s, 1H), 3.38 (s, 1H), 2.58 (dd, $J$ = 13.8, 2.5 Hz, 1H), 2.47 (m, 1H), 2.32 (s, 3H), 2.17 (m, 2H), 1.86 (m, 1H), 1.63 (m, 1H) ppm. **$^{13}$C NMR** (176 MHz, Chloroform-*d*) $\delta$ 186.7, 171.4, 167.3, 157.6, 154.1, 153.8, 141.4, 139.3, 128.9, 126.2, 122.1,

121.2, 116.9, 75.4, 63.6, 62.8, 56.8, 55.0, 35.8, 34.3, 33.7, 33.3, 21.2, 20.5 ppm. **HRMS**-ESI (*m/z*): $[M + H]^+$ calculated for $C_{23}H_{24}NO_6$ $[M+H]^+$ = 410.1598, found 410.1596.

(*S*)-2-((2',3,3',4,5',6'-hexahydro-4a*H*-spiro[pyrano[3,4-b]indole-1,4'-thiopyran]-4a-yl)methyl)-4-oxo-4H-pyran-3-yl acetate (**3d**)

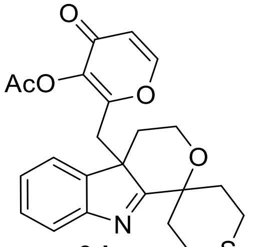

**3d**

White amorph solid (26%). $^1$**H NMR** (700 MHz, Chloroform-*d*) δ 7.60 (d, *J* = 7.7 Hz, 1H), 7.37 (d, *J* = 5.7 Hz, 1H), 7.33 (t, *J* = 7.6 Hz, 1H), 7.17 (m, 2H), 6.23 (d, *J* = 5.7 Hz, 1H), 4.11 (t, *J* = 7.0 Hz, 1H), 3.83 (ddd, *J* = 11.7, 8.1, 3.3 Hz, 1H), 3.51 (d, *J* = 15.1 Hz, 1H), 3.39 (d, *J* = 15.1 Hz, 1H), 3.17 (t, *J* = 11.7 Hz, 1H), 3.03 (t, *J* = 12.2 Hz, 1H), 2.58 (m, 2H), 2.47 (m, 3H), 2.33 (s, 3H), 2.24(m, 2H), 1.57 (dt, *J* = 14.1, 8.4 Hz, 1H) ppm. $^{13}$**C NMR** (176 MHz, Chloroform-*d*) δ 187.9, 171.3, 167.2, 157.6, 153.6, 140.9, 139.2, 128.8, 126.0, 122.1, 121.0, 116.8, 76.3, 56.2, 54.9, 35.3, 34.0, 34.0, 33.0, 26.9, 23.3, 23.0, 20.4 ppm. **HRMS**-ESI (*m/z*): $[M + H]^+$ calculated for $C_{23}H_{24}NO_5S$ $[M+H]^+$ = 426.1370, found 426.1368.

2-(((2*S*,4a'*S*,5*R*)-2-isopropyl-5-methyl-3',4'-dihydro-4a'*H*-spiro[cyclohexane-1,1'-pyrano[3,4-b]indol]-4a'-yl)methyl)-4-oxo-4H-pyran-3-yl acetate (**3e**)

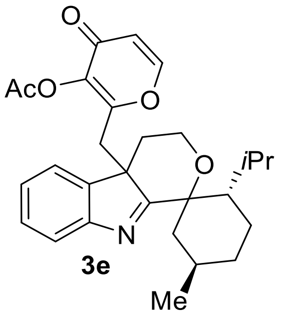

**3e**

White pale solid (36%). $^1$**H NMR** (700 MHz, Chloroform-*d*) δ 7.59 (d, *J* = 7.7 Hz, 1H), 7.42 (d, *J* = 5.7 Hz, 1H), 7.32 (t, *J* = 7.6 Hz, 1H), 7.15 (t, *J* = 7.4 Hz, 1H), 7.05 (d, *J* = 7.4 Hz, 1H), 6.26 – 6.23 (m, 1H), 4.16 (td, *J* = 12.1, 2.1 Hz, 1H), 3.94 (dd, *J* = 12.0, 4.7 Hz, 1H), 3.46 (d, *J* = 14.8 Hz, 1H), 3.07 (d, *J* = 14.7 Hz, 1H), 2.44 (d, *J* = 13.7 Hz, 1H), 2.41 – 2.36 (m, 1H), 2.25 (s, 3H), 2.15 – 2.10

(m, 1H), 1.87 – 1.78 (m,2H), 1.80 – 1.74 (m, 1H), 1.72 (td, *J* = 13.1, 5.2 Hz, 1H), 1.59 (td, *J* = 10.2, 8.5, 3.1 Hz, 2H), 1.25 – 1.19 (m, 1H), 1.03 (dtd, *J* = 15.8, 7.4, 6.7, 4.4 Hz, 1H), 0.93 (t, *J* = 6.8 Hz, 6H), 0.77 (d, *J* = 6.9 Hz, 3H) ppm. **$^{13}$C NMR** (176 MHz, Chloroform-*d*) *δ* 185.6, 171.3, 167.1, 157.6, 153.7, 141.1, 139.5, 128.8, 125.7, 121.8, 120.9, 116.8, 82.8, 57.2, 54.8, 47.8, 41.8, 35.4, 34.6, 33.9, 27.8, 27.6, 26.9, 23.7, 22.4, 21.3, 20.3, 18.7. **HRMS**-ESI (*m/z*): [M + H]$^+$ calculated for $C_{28}H_{34}NO_5$ [M+H]$^+$ = 464.2432, found 464.2431 (-0.9372 ppm). $[\alpha]_D^{20}$ +33.6 (*c* 0.11, $CHCl_3$).

(*S*)-2-((6'-bromo-3',4'-dihydro-4a'H-spiro[cyclohexane-1,1'-pyrano[3,4-b]indol]-4a'-yl)methyl)-4-oxo-4H-pyran-3-yl acetate (**3f**)

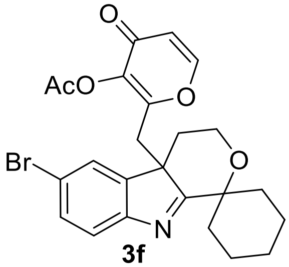


**3f**

Yellow solid (56 %). **$^1$H NMR** (700 MHz, Chloroform-*d*) *δ* 7.45 (d, *J* = 1.5 Hz, 2H), 7.41 (d, *J* = 5.7 Hz, 1H), 7.29 (s, 1H), 6.27 (d, *J* = 5.7 Hz, 1H), 4.09 (dt, *J* = 12.3, 7.7 Hz, 1H), 3.81 (ddd, *J* = 11.9, 8.2, 3.5 Hz, 1H), 3.50 (d, *J* = 15.0 Hz, 1H), 3.37 (d, *J* = 15.0 Hz, 1H), 2.55 (ddd, *J* = 13.8, 7.4, 3.4 Hz, 1H), 2.36 (s, 3H), 2.18 (d, *J* = 12.9 Hz, 1H), 2.01 (td, *J* = 13.3, 12.5, 4.0 Hz, 1H), 1.96 (d, *J* = 12.0 Hz, 1H), 1.87 (td, *J* = 13.1, 4.0 Hz, 1H), 1.79 – 1.73 (m, 2H), 1.70 – 1.66 (m, 1H), 1.66 – 1.62 (m, 1H), 1.62 – 1.54 (m, 2H), 1.40 (ddd, *J* = 16.1, 8.1, 3.8 Hz, 1H) ppm. **$^{13}$C NMR** (176 MHz, Chloroform-*d*) *δ* 190.4, 171.3, 167.1, 157.1, 153.6, 143.3, 139.4, 131.8, 125.9, 121.9, 119.5, 116.9, 77.9, 56.0, 55.6, 34.3, 33.5, 33.3, 33.2, 25.2, 21.2, 21.0, 20.4 ppm. **HRMS**-ESI (*m/z*): [M + H]$^+$ calculated for $C_{24}H_{25}BrNO_5$ [M+H]$^+$ = 486.0911, found 486.0910.

(*S*)-2-((8'-ethyl-3',4'-dihydro-4a'H-spiro[cyclohexane-1,1'-pyrano[3,4-b]indol]-4a'-yl)methyl)-4-oxo-4H-pyran-3-yl acetate (**3g**)

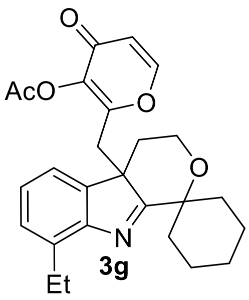

3g

Yellow solid (55%). **$^1$H NMR** (700 MHz, Chloroform-*d*) *δ* 7.37 (d, *J* = 5.7 Hz, 1H), 7.12 (d, *J* = 7.5 Hz, 1H), 7.06 (t, *J* = 7.5 Hz, 1H), 6.93 (d, *J* = 7.3 Hz, 1H), 6.23 (d, *J* = 5.7 Hz, 1H), 4.08 (dt, *J* = 12.2, 7.8 Hz, 1H), 3.79 (ddd, *J* = 11.7, 8.0, 3.5 Hz, 1H), 3.49 (d, *J* = 15.0 Hz, 1H), 3.32 (d, *J* = 14.8 Hz, 1H), 2.95 (ddt, *J* = 34.4, 14.1, 7.3 Hz, 2H), 2.53 (ddd, *J* = 13.7, 7.2, 3.6 Hz, 1H), 2.32 (s, 3H), 2.15 (d, *J* = 13.7 Hz, 1H), 2.11 – 2.06 (m, 1H), 1.92 (t, *J* = 12.4 Hz, 2H), 1.79 – 1.73 (m, 2H), 1.65 – 1.60 (m, 2H), 1.56 (dt, *J* = 13.8, 8.3 Hz, 1H), 1.43 (qd, *J* = 12.0, 3.7 Hz,1H), 1.25 (t, *J* = 7.6 Hz, 3H) ppm. **$^{13}$C NMR** (176 MHz, Chloroform-*d*) *δ* 187.5, 171.5, 167.2, 158.2, 153.6, 141.3, 139.2, 136.8, 128.1, 125.6, 119.5, 116.7, 78.0, 56.2, 54.9, 34.4, 34.1, 33.4, 33.3, 25.3, 25.1, 24.3, 21.4, 21.2, 20.4, 15.4 ppm. **HRMS**-ESI (*m/z*): $[M + H]^+$ calculated for $C_{25}H_{28}NO_6$ $[M+H]^+$ = 438.1912, found 436.2115.

## Synthesis of Griseofulvin-Indolenines and Indolines (7 and 8)

General Procedure for the Synthesis of Griseofulvin-Indolenines (**7**)

An argon flushed round-bottom flask was charged with the corresponding indole substrate (1.0 eq.). After dissolving the starting material in anhydrous $CH_2Cl_2$ (1 M), 2-(bromomethyl)-3-((tert-butyldimethylsilyl)oxy)-4H-pyran-4-one (2.0 eq.) **2** dissolved in TFE (0.32 M) was added. The reaction mixture was heated to 50 °C and continuously stirred for 30 min. After complete conversion indicated by TLC and LC-MS analysis, the solvent was removed *in vacuo* and directly applied to the next step.

The crude indolenine (1.0 eq.) was dissolved in anhydrous $CH_2Cl_2$ (2 ml) under argon atmosphere. After catalytic amounts of DMAP (0.1 eq.) and $Et_3N$ (3.0 eq.) were added, the mixture was stirred (5 min at 0 °C). $(Ac)_2O$ (2.4 eq.) was added and the dark reaction mixture was stirred for at 22 °C. After complete conversion, the reaction was quenched with $NaHCO_3$ (5 ml) and the aqueous layer was extracted with EtOAc (3 x 5 ml). The combined organic layers were dried over $NaSO_4$ and the solvent was removed *in vacuo*. The crude product was purified either by flash column chromatography (CH/EtOAc 100% v/v) or gel perfusion chromatography to isolate the desired compound.

General Procedure for the Reduction of Griseofulvin-Indolenines to Griseofulvin Indolines (**8**)

The acetylated spiro-indolenine was dissolved in anhydrous methanol (0.5 mL) under argon atmosphere. After the addition of $NaBH_3CN$ (1.5 eq.), the reaction was stirred for 15 min at 22 °C. The reaction mixture was quenched with brine (3 mL) and the aqueous layer was extracted with EtOAc (3 x 5ml). The combined organic layers were dried over $NaSO_4$ and the solvent was removed *in vacuo*. The crude product was purified by flash column chromatography (CH/EtOAc 40-80% v/v) to afford the desired compound. The yield was determined over three steps.

## Griseofulvin-Indolenine (7)

2-(((2*R*,2'*R*,4a'*S*)-7-chloro-4,6-dimethoxy-2'-methyl-3-oxo-1',2'-dihydro-3H-spiro[benzofuran-2,3'-carbazol]-4a'(4'H)-yl)methyl)-4-oxo-4H-pyran-3-yl acetate (**7a**)

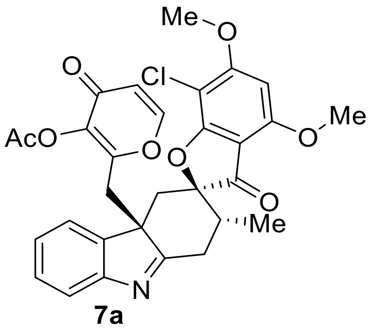


**7a**

White crystalline solid (98%). **$^1$H NMR** (500 MHz, Chloroform-*d*) $\delta$ 7.56 (d, $J$ = 7.8 Hz, 1H), 7.33 (m, 1H), 7.27 (s, 1H), 7.24 (d, $J$ = 6.9 Hz, 1H), 7.19 (t, $J$ = 7.4 Hz, 1H), 6.15 (s, 1H), 6.15 (d, $J$ = 5.5 Hz, 1H), 4.04 (s, 3H), 4.03 (d, $J$ = 8.4 Hz, 1H), 3.98 (s, 3H), 3.65 (dd, $J$ = 12.9, 5.4 Hz, 1H), 3.28 (d, $J$ = 14.6 Hz, 1H), 2.86 (d, $J$ = 12.7 Hz, 1H), 2.48 (m, 1H), 2.39 (m, 1H), 2.29 (s, 3H), 2.08 (d, $J$ = 15.1 Hz, 1H), 1.00 (d, $J$ = 7.2 Hz, 3H) ppm**.** **$^{13}$C NMR** (126 MHz, Chloroform-*d*) $\delta$ 195.4, 183.5, 171.5, 167.1, 166.9, 164.5, 158.2, 157.4, 154.0, 153.5, 141.8, 139.3, 129.0, 125.9, 122.4, 120.7, 116.6, 104.6, 97.8, 91.8, 89.6, 57.2, 56.6, 56.1, 40.6, 39.4, 34.5, 34.0, 20.6, 13.5 ppm. **HRMS**-ESI (*m/z*): [M + H]$^+$ calculated for $C_{30}H_{27}ClNO_8$ [M+H]$^+$ = 564.1420, found 564.1416.

2-(((2*R*,2'*R*,4a'*S*)-7-chloro-4,6-dimethoxy-2',6'-dimethyl-3-oxo-1',2'-dihydro-3H-spiro [benzofuran-2,3'-carbazol]-4a'(4'H)-yl)methyl)-4-oxo-4H-pyran-3-yl acetate (**7b**)

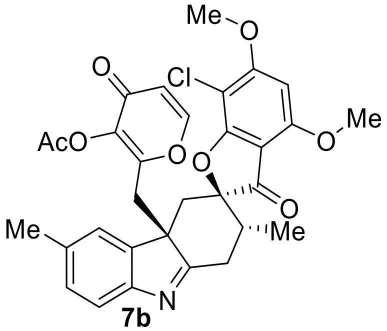


**7b**

Yellow pale solid (78%). **$^1$H NMR** (500 MHz, Chloroform-*d*) $\delta$ 7.44 (d, $J$ = 7.8 Hz, 1H), 7.31 (d, $J$ = 5.8 Hz, 1H), 7.13 (d, $J$ = 7.9 Hz, 1H), 7.02 (s, 1H), 6.16 (d, $J$ = 5.8 Hz, 1H), 6.15 (s, 1H), 4.04 (s, 3H), 3.98 (s, 3H), 3.98 (d, $J$ = 14.5 Hz, 1H), 3.63 (dd, $J$ = 13.0, 5.3 Hz, 1H), 3.28 (d, $J$ = 14.6 Hz, 1H), 2.86 (d, $J$ = 12.5 Hz, 1H), 2.47 (m, 1H), 2.36 (s, 3H), 2.36 (m, 1H), 2.28 (s, 3H), 2.08 (d,

$J$ = 15.3 Hz, 1H), 0.99 (d, $J$ = 7.2 Hz, 3H) ppm. **$^{13}$C NMR** (126 MHz, Chloroform-*d*) $\delta$ 195.3, 180.9, 171.4, 166.8, 166.8, 164.3, 158.1, 157.3, 153.9, 150.9, 141.7, 139.2, 135.7, 129.5, 123.1, 119.9, 116.5, 104.4, 97.7, 91.7, 89.5, 57.0, 56.4, 55.7, 40.5, 39.2, 34.4, 33.7, 21.5, 20.4, 13.3 ppm. **HRMS**-ESI (*m/z*): [M + H]$^+$ calculated for $C_{31}H_{29}ClNO_8$ [M+H]$^+$ = 578.1576 found 578.1574. $[\alpha]_D^{20}$ +33.8 (*c* 0.23, $CHCl_3$).

2-(((2*R*,2'*R*,4a'*S*)-7-chloro-4,6-dimethoxy-2',5',7'-trimethyl-3-oxo-1',2'-dihydro-3H-spiro [benzofuran-2,3'-carbazol]-4a'(4'H)-yl)methyl)-4-oxo-4H-pyran-3-yl acetate (**7c**)

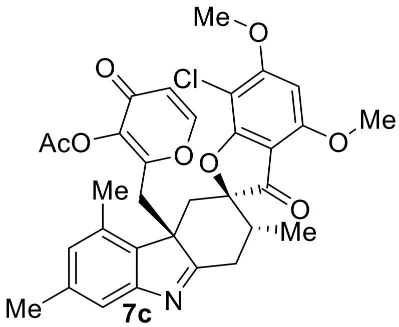

**7c**

Orange amorph solid (95%). **$^1$H NMR** (500 MHz, Chloroform-*d*) $\delta$ 7.26 (d, $J$ = 3.4 Hz, 1H), 7.14 (s, 1H), 6.75 (s, 1H), 6.15 (s, 1H), 6.13 (d, $J$ = 5.8 Hz, 1H), 4.09 (d, $J$ = 14.8 Hz, 1H), 4.03 (s, 3H), 3z.98 (s, 3H), 3.62 (dd, $J$ = 13.0, 5.3 Hz, 1H), 3.36 (d, $J$ = 14.6 Hz, 1H), 2.79 (d, $J$ = 11.7 Hz, 1H), 2.50 (dd, $J$ = 15.2, 1.6 Hz, 1H), 2.44 (m, 1H), 2.33 (s, 3H), 2.32 (s, 3H), 2.32 (s, 3H), 2.19 (d, $J$ = 15.1 Hz, 1H), 0.97 (d, $J$ = 7.3 Hz, 3H) ppm. **$^{13}$C NMR** (126 MHz, Chloroform-*d*) $\delta$ 195.6, 182.7, 171.4, 167.0, 166.8, 164.4, 158.1, 157.4, 154.3, 153.9, 139.2, 138.8, 136.3, 132.9, 128.5, 118.9, 116.4, 104.5, 97.7, 91.8, 89.4, 57.1, 56.4, 56.3, 39.9, 38.3, 33.7, 33.0, 21.4, 20.5, 18.1, 13.3 ppm. **HRMS**-ESI (*m/z*): [M + H]$^+$ calculated for $C_{32}H_{31}ClNO_8$ [M+H]$^+$ = 592.1733 found 592.1731. $[\alpha]_D^{20}$ –9.5 (*c* 0.32, $CHCl_3$).

2-(((2*R*,2'*R*,4a'*S*)-7-chloro-6'-isopropyl-4,6-dimethoxy-2'-methyl-3-oxo-1',2'-dihydro-3H-spiro [benzofuran-2,3'-carbazol]-4a'(4'H)-yl)methyl)-4-oxo-4H-pyran-3-yl acetate (**7d**)

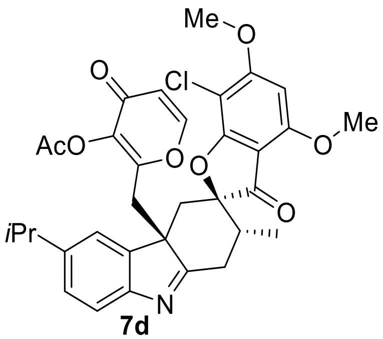

**7d**

Yellow pale solid (77%). **$^1$H NMR** (700 MHz, Chloroform-*d*) $\delta$ 7.44 (d, $J$ = 8.0 Hz, 1H), 7.25 (d, $J$ = 5.7 Hz, 1H), 7.16 (dd, $J$ = 8.1, 1.6 Hz, 1H), 7.09 (d, $J$ = 1.5 Hz, 1H), 6.15 (s, 1H), 6.13 (d, $J$ = 5.7 Hz, 1H), 4.04 (s, 3H), 3.98 (s, 3H), 3.91 (d, $J$ = 14.5 Hz, 1H), 3.62 (dd, $J$ = 13.0, 5.4 Hz, 1H), 3.29 (d, $J$ = 14.6 Hz, 1H), 2.90 (hept, $J$ = 6.8 Hz, 1H), 2.82 (d, $J$ = 12.9 Hz, 1H), 2.45 (m, 1H), 2.37 (dd, $J$ = 15.2, 1.9 Hz, 1H), 2.28 (s, 3H), 2.08 (d, $J$ = 15.2 Hz, 1H), 1.21 (dd, $J$ = 9.4, 6.9 Hz, 6H), 0.99 (d, $J$ = 7.3 Hz, 3H) ppm. **$^{13}$C NMR** (176 MHz, Chloroform-*d*) $\delta$ 195.5, 182.5, 171.4, 167.2, 167.0, 164.5, 158.2, 157.7, 153.9, 151.7, 146.8, 141.9, 139.3, 127.1, 120.4, 120.2, 116.6, 104.6, 97.9, 92.0, 89.6, 57.1, 56.5, 56.0, 40.5, 39.4, 34.6, 34.3, 33.9, 24.4, 24.2, 20.5, 13.4 ppm. **HRMS**-ESI (*m/z*): [M + H]$^+$ calculated for $C_{33}H_{33}ClNO_8$ [M+H]$^+$ = 606.1889 found 606.1886. $[\alpha]_D^{20}$ +25.0 (*c* 0.26, $CHCl_3$).

2-(((2*R*,2'*R*,4a'*S*)-7-chloro-4,6-dimethoxy-2'-methyl-3-oxo-6'-phenyl-1',2'-dihydro-3H-spiro [benzofuran-2,3'-carbazol]-4a'(4'H)-yl)methyl)-4-oxo-4H-pyran-3-yl acetate (**7e**)

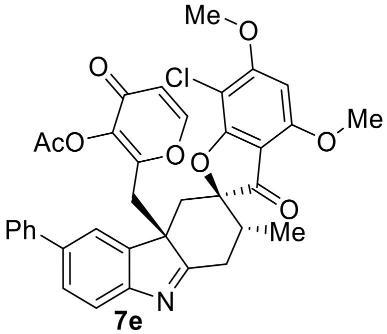

**7e**

Yellow pale solid (91%). **$^1$H NMR** (500 MHz, Chloroform-*d*) $\delta$ 7.62 (d, $J$ = 7.9 Hz, 1H), 7.55 (ddd, $J$ = 7.2, 5.2, 2.7 Hz, 3H), 7.44 (m, 3H), 7.35 (d, $J$ = 7.3 Hz, 1H), 7.30 (d, $J$ = 5.8 Hz, 1H), 6.16 (d, $J$ = 5.6 Hz, 2H), 4.06 (d, $J$ = 19.4 Hz, 1H), 4.05 (s, 3H), 3.99 (s, 3H), 3.69 (dd, $J$ = 13.0, 5.3 Hz, 1H), 3.33 (d, $J$ = 14.5 Hz, 1H), 2.90 (d, $J$ = 12.7 Hz, 1H), 2.50 (m, 1H), 2.43 (m, 1H), 2.18 (s, 3H), 2.15 (d, $J$ = 13.4 Hz, 1H), 1.02 (d, $J$ = 7.3 Hz, 3H) ppm. **$^{13}$C NMR** (126 MHz, Chloroform-*d*) $\delta$

195.2, 183.7, 171.3, 166.9, 166.8, 164.4, 158.1, 157.1, 153.9, 152.5, 142.3, 140.9, 139.3, 128.8, 128.1, 127.4, 127.2, 121.4, 120.6, 116.5, 104.4, 97.7, 91.6, 89.5, 57.0, 56.4, 56.1, 40.5, 39.3, 34.6, 33.9, 20.3, 13.4 ppm. **HRMS**-ESI (*m/z*): $[M + H]^+$ calculated for $C_{36}H_{31}ClNO_8$ $[M+H]^+$ = 640.1733, found 640.1732. $[\alpha]_D^{20}$ $+110.1$ (*c* 0.1, $CHCl_3$).

2-(((2*R*,6b'*S*,9'*R*)-7-chloro-4,6-dimethoxy-9'-methyl-3-oxo-9',10'-dihydro-3H-spiro[benzofuran-2,8'-benzo[a]carbazole]-6b'(7'H)-yl)methyl)-4-oxo-4H-pyran-3-yl acetate (**7f**)

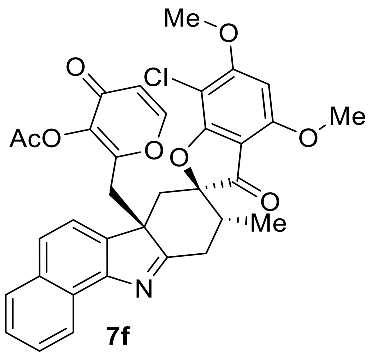


**7f**

White pale solid (58%). **$^1$H NMR** (700 MHz, Chloroform-*d*) *δ* 8.56 (d, *J* = 8.2 Hz, 1H), 7.87 (d, *J* = 8.2 Hz, 1H), 7.71 (d, *J* = 8.2 Hz, 1H), 7.58 (t, *J* = 7.5 Hz, 1H), 7.51 (t, *J* = 7.5 Hz, 1H), 7.37 (d, *J* = 8.3 Hz, 1H), 7.15 (d, *J* = 5.3 Hz, 1H), 6.16 (s, 1H), 6.05 (d, *J* = 5.7 Hz, 1H), 4.08 (d, *J* = 14.7 Hz, 1H), 4.05 (s, 3H), 3.98 (s, 3H), 3.74 (dd, *J* = 13.0, 5.2 Hz, 1H), 3.39 (d, *J* = 14.7 Hz, 1H), 3.00 (d, *J* = 10.7 Hz, 1H), 2.53 (s, 1H), 2.48 (d, *J* = 15.3 Hz, 1H), 2.29 (s, 3H), 2.06 (d, *J* = 15.2 Hz, 1H), 1.01 (d, *J* = 7.2 Hz, 3H) ppm. **$^{13}$C NMR** (176 MHz, Chloroform-*d*) *δ* 195.4, 183.5, 171.3, 167.0, 166.9, 164.4, 158.1, 157.3, 153.7, 149.5, 139.1, 138.4, 134.1, 128.0, 127.3, 126.5, 126.2, 126.1, 123.6, 119.5, 116.5, 104.5, 97.8, 91.7, 89.5, 57.0, 56.4, 42.6, 40.8, 39.5, 34.1, 34.0, 20.4, 13.3 ppm. **HRMS**-ESI (*m/z*): $[M + H]^+$ calculated for $C_{34}H_{29}ClNO_8$ $[M+H]^+$ = 614.1576 found 614.1578. $[\alpha]_D^{20}$ $-8.6$ (*c* 0.20, $CHCl_3$).

2-(((2*R*,2'*R*,4a'*S*)-7-chloro-4,6,6'-trimethoxy-2'-methyl-3-oxo-1',2'-dihydro-3H-spiro [benzofuran-2,3'-carbazol]-4a'(4'H)-yl)methyl)-4-oxo-4H-pyran-3-yl acetate (**7g**)

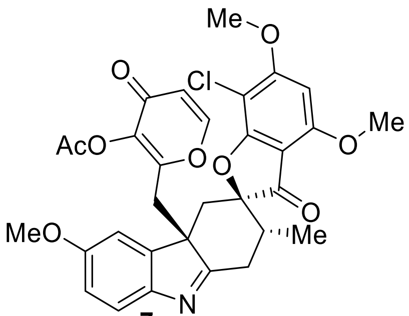

**7g**

Yellow pale solid (72%). **$^1$H NMR** (700 MHz, Chloroform-*d*) *δ* 7.45 (d, *J* = 8.5 Hz, 1H), 7.32 (d, *J* = 5.7 Hz, 1H), 6.84 (dd, *J* = 8.5, 2.5 Hz, 1H), 6.79 (d, *J* = 2.4 Hz, 1H), 6.17 (d, *J* = 5.8 Hz, 1H), 6.15 (s, 1H), 4.04 (s, 3H), 4.01 (m, 1H), 3.98 (s, 3H), 3.79 (s, 3H), 3.61 (dd, *J* = 13.0, 5.3 Hz, 1H), 3.26 (d, *J* = 14.6 Hz, 1H), 2.81 (d, *J* = 12.5 Hz, 1H), 2.45 (m, 2H), 2.33 (d, *J* = 15.3 Hz, 1H), 2.28 (s, 3H), 2.07 (d, *J* = 15.2 Hz, 1H), 0.99 (d, *J* = 7.3 Hz, 3H) ppm. **$^{13}$C NMR** (176 MHz, Chloroform-*d*) *δ* 195.3, 181.0, 171.3, 166.9, 166.8, 164.3, 158.2, 158.1, 157.3, 153.9, 146.9, 143.3, 139.2, 120.8, 116.5, 113.3, 109.3, 104.4, 97.7, 91.7, 89.5, 57.0, 56.4, 56.0, 55.7, 40.4, 39.2, 34.5, 33.7, 20.4, 13.3 ppm. **HRMS**-ESI (*m/z*): [M + H]$^+$ calculated for $C_{31}H_{29}ClNO_9$ [M+H]$^+$ = 594.1525 found 594.1527. $[\alpha]_D^{20}$ +30.2 (*c* 0.23, $CHCl_3$).

2-(((2*R*,2'*R*,4a'*S*)-6'-(benzyloxy)-7-chloro-4,6-dimethoxy-2'-methyl-3-oxo-1',2'-dihydro-3H-spiro [benzofuran-2,3'-carbazol]-4a'(4'H)-yl)methyl)-4-oxo-4H-pyran-3-yl acetate (**7h**)

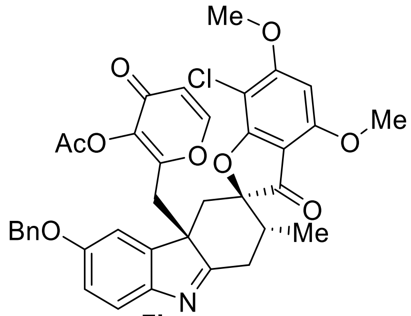

**7h**

Yellow pale solid (yield = 90%). **$^1$H NMR** (500 MHz, Chloroform-*d*) *δ* 7.47 (d, *J* = 8.4 Hz, 1H), 7.40 (m, 4H), 7.34 (m, 1H), 7.26 (s, 1H), 6.92 (dd, *J* = 8.5, 2.4 Hz, 1H), 6.88 (d, *J* = 2.4 Hz, 1H), 6.17 (d, *J* = 5.7 Hz, 1H), 6.15 (s, 1H), 5.05 (s, 2H), 4.04 (s, 3H), 4.04 (d, *J* = 14.8 Hz, 1H), 3.98 (s, 3H), 3.63 (dd, *J* = 13.1, 5.4 Hz, 1H), 3.24 (d, *J* = 14.7 Hz, 1H), 2.88 (d, *J* = 12.7 Hz, 1H), 2.47 (m, 1H), 2.33 (dd, *J* = 15.1, 2.0 Hz, 1H), 2.29 (s, 3H), 2.10 (d, *J* = 15.2 Hz, 1H), 1.00 (d, *J* = 7.3 Hz, 3H) ppm. **$^{13}$C NMR** (126 MHz, Chloroform-*d*) *δ* 195.4, 181.8, 171.5, 167.0 (d, *J* = 15.1 Hz), 164.5,

158.2, 157.7, 157.2, 154.0, 143.3, 139.4, 136.8, 128.8, 128.3, 127.7, 120.8, 116.7, 114.4, 110.6, 104.5, 97.8, 91.8, 89.6, 70.7, 57.2, 56.6, 56.2, 40.7, 39.4, 34.7, 33.8, 20.6, 13.5 ppm. **HRMS**-ESI (*m/z*): $[M + H]^+$ calculated for $C_{37}H_{33}ClNO_9$ $[M+H]^+$ = 670.1838 found 670.1839. $[\alpha]_D^{20}$ +40.5 (*c* 0.24, $CHCl_3$).

2-(((2*R*,2'*R*,4a'*S*)-7-chloro-4,6-dimethoxy-2'-methyl-3-oxo-6'-(trifluoromethoxy)-1',2'-dihydro-3H-spiro [benzofuran-2,3'-carbazol]-4a'(4'H)-yl)methyl)-4-oxo-4H-pyran-3-yl acetate (**7i**)

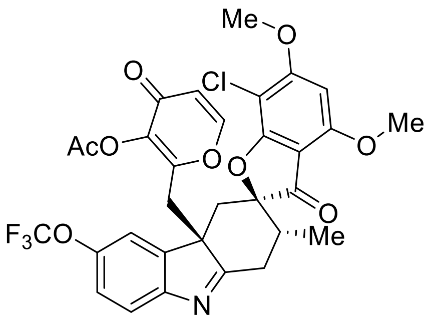


**7i**

Yellow pale solid (92%). **$^1$H NMR** (500 MHz, Chloroform-*d*) $\delta$ 7.56 (d, *J* = 8.5 Hz, 1H), 7.27 (d, *J* = 5.6 Hz, 1H), 7.21 (d, *J* = 8.5 Hz, 1H), 7.15 (s, 1H), 6.16 (m, 2H), 4.08 (d, *J* = 14.5 Hz, 1H), 4.05 (s, 3H), 3.99 (s, 3H), 3.66 (dd, *J* = 13.0, 5.4 Hz, 1H), 3.23 (d, *J* = 14.6 Hz, 1H), 2.85 (dd, *J* = 12.9, 1.9 Hz, 1H), 2.50 (dd, *J* = 7.2, 5.5 Hz, 1H), 2.38 (dd, *J* = 15.2, 1.8 Hz, 1H), 2.31 (s, 3H), 2.10 (d, *J* = 15.1 Hz, 1H), 0.99 (d, *J* = 7.3 Hz, 3H) ppm. **$^{13}$C NMR** (126 MHz, Chloroform-*d*) $\delta$ 195.0, 184.8, 171.2, 167.0, 166.7, 164.5, 158.1, 156.5, 153.8, 151.8, 147.1, 143.3, 139.3, 122.0, 121.2, 119.4, 116.6, 115.9, 104.3, 97.7, 91.3, 89.6, 57.1, 56.7, 56.4, 40.4, 39.0, 34.5, 33.9, 20.3, 13.3 ppm. **HRMS**-ESI (*m/z*): $[M + H]^+$ calculated for $C_{31}H_{26}ClF_3NO_9$ $[M+H]^+$ = 648.1243 found 648.1242. $[\alpha]_D^{20}$ −1.9 (*c* 0.21, $CHCl_3$).

2-(((2*R*,2'*R*,4a'*S*)-7-chloro-4,6-dimethoxy-2'-methyl-3-oxo-6'-(trifluoromethyl)-1',2'-dihydro-3H-spiro[benzofuran-2,3'-carbazol]-4a'(4'H)-yl)methyl)-4-oxo-4H-pyran-3-yl acetate (**7j**)

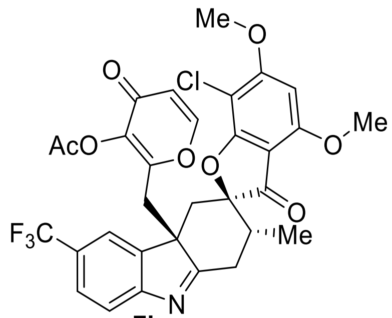

**7j**

Yellow pale solid (74%). **$^1$H NMR** (700 MHz, Chloroform-*d*) $\delta$ 7.64 (m, 2H), 7.54 (s, 1H), 7.30 (d, *J* = 5.7 Hz, 1H), 6.16 (m, 2H), 4.10 (d, *J* = 14.5 Hz, 1H), 4.05 (s, 3H), 3.99 (s, 3H), 3.71 (dd, *J* = 13.0, 5.4 Hz, 1H), 3.29 (d, *J* = 14.6 Hz, 1H), 2.89 (dd, *J* = 13.0, 2.1 Hz, 1H), 2.51 (dd, *J* = 7.3, 5.4 Hz, 1H), 2.43 (dd, *J* = 15.2, 1.9 Hz, 1H), 2.28 (s, 3H), 2.10 (d, *J* = 15.2 Hz, 1H), 0.98 (d, *J* = 7.3 Hz, 3H) ppm. **$^{13}$C NMR** (176 MHz, Chloroform-*d*) $\delta$ 194.8, 186.7, 171.1, 166.9, 166.7, 164.5, 158.1, 156.3, 156.1, 153.8, 142.3, 139.4, 126.6, 125.0, 123.4, 120.7, 119.6, 116.6, 104.3, 97.7, 91.2, 89.6, 57.0, 56.6, 56.4, 40.5, 39.0, 34.6, 34.1, 20.2, 13.3 ppm. **HRMS**-ESI (*m/z*): [M + H]$^+$ calculated for $C_{31}H_{26}ClF_3NO_8$ [M+H]$^+$ = 632.1294 found 632.1295. $[\alpha]_D^{20}$ +14.2 (*c* 0.24, $CHCl_3$).

2-(((2*R*,2'*R*,4a'*S*)-7-chloro-6'-fluoro-4,6-dimethoxy-2'-methyl-3-oxo-1',2'-dihydro-3H-spiro [benzofuran-2,3'-carbazol]-4a'(4'H)-yl)methyl)-4-oxo-4H-pyran-3-yl acetate (**7k**)

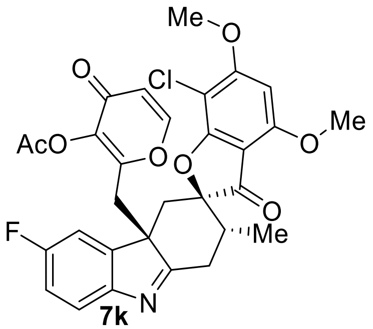

**7k**

Yellow pale solid (94%). **$^1$H NMR** (500 MHz, Chloroform-*d*) $\delta$ 7.50 (dd, *J* = 8.5, 4.5 Hz, 1H), 7.32 (d, *J* = 5.8 Hz, 1H), 7.04 (td, *J* = 8.8, 2.4 Hz, 1H), 6.97 (dd, *J* = 7.7, 2.5 Hz, 1H), 6.18 (d, *J* = 5.8 Hz, 1H), 6.16 (s, 1H), 4.07 (d, *J* = 14.6 Hz, 1H), 4.04 (s, 3H), 3.99 (s, 3H), 3.65 (dd, *J* = 13.0, 5.3 Hz, 1H), 3.23 (d, *J* = 14.6 Hz, 1H), 2.84 (d, *J* = 11.9 Hz, 1H), 2.48 (m, 1H), 2.36 (dd, *J* = 15.1, 1.8 Hz, 1H), 2.31 (s, 3H), 2.08 (d, *J* = 15.1 Hz, 1H), 0.99 (d, *J* = 7.3 Hz, 3H) ppm. **$^{13}$C NMR** (126 MHz, Chloroform-*d*) $\delta$ 195.0, 183.4, 171.3, 166.9, 166.7, 164.4, 162.2, 160.2, 158.1, 156.7, 149.3, 143.6, 139.3, 121.3, 116.6, 115.6, 110.3, 104.3, 97.7, 91.4, 89.5, 57.1, 56.5, 56.4, 40.4, 39.0,

34.5, 33.8, 20.4, 13.3 ppm. **HRMS**-ESI (*m/z*): $[M + H]^+$ calculated for $C_{30}H_{26}ClFNO_8$ $[M+H]^+$ = 582.1326 found 582.1323. $[\alpha]_D^{20}$ $-0.33$ (*c* 0.30, $CHCl_3$).

2-(((2*R*,2'*R*,4a'*S*)-6',7-dichloro-4,6-dimethoxy-2'-methyl-3-oxo-1',2'-dihydro-3H-spiro [benzofuran-2,3'-carbazol]-4a'(4'H)-yl)methyl)-4-oxo-4H-pyran-3-yl acetate (**7l**)

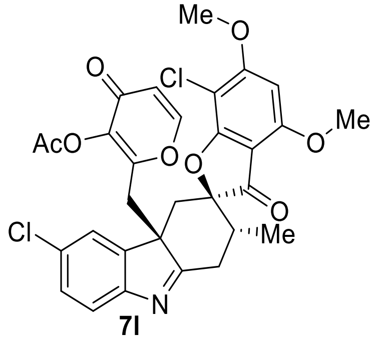

**7l**

Colourless solid (98%). **$^{1}$H NMR** (500 MHz, Chloroform-*d*) *δ* 7.46 (d, *J* = 8.4 Hz, 1H), 7.34 (d, *J* = 5.6 Hz, 1H), 7.31 (dd, *J* = 8.2, 2.0 Hz, 1H), 7.24 (d, *J* = 2.0 Hz, 1H), 6.18 (d, *J* = 5.6 Hz, 1H), 6.16 (s, 1H), 4.07 (d, *J* = 14.6 Hz, 1H), 4.04 (s, 3H), 3.98 (s, 3H), 3.65 (dd, *J* = 13.0, 5.4 Hz, 1H), 3.23 (d, *J* = 14.5 Hz, 1H), 2.82 (dd, *J* = 13.0, 1.8 Hz, 1H), 2.47 (dd, *J* = 7.1, 5.4 Hz, 1H), 2.35 (m, 1H), 2.31 (s, 3H), 2.08 (d, *J* = 15.1 Hz, 1H), 0.97 (d, *J* = 7.2 Hz, 3H) ppm. **$^{13}$C NMR** (126 MHz, Chloroform-*d*) *δ* 195.0, 183.8, 171.2, 166.9, 166.7, 164.4, 158.1, 156.6, 153.9, 152.1, 143.5, 139.3, 131.6, 129.1, 123.0, 121.4, 116.6, 104.3, 97.7, 91.4, 89.6, 57.1, 56.5, 56.4, 40.4, 38.9, 34.6, 33.9, 20.4, 13.3 ppm. **HRMS**-ESI (*m/z*): $[M + H]^+$ calculated for $C_{30}H_{26}Cl_2NO_8$ $[M+H]^+$ = 598.1030 found 598.1030. $[\alpha]_D^{20}$ $+$ 39.6 (*c* 0.21, $CHCl_3$).

2-(((2*R*,2'*R*,4a'*S*)-7-chloro-6'-iodo-4,6-dimethoxy-2'-methyl-3-oxo-1',2'-dihydro-3H-spiro [benzofuran-2,3'-carbazol]-4a'(4'H)-yl)methyl)-4-oxo-4H-pyran-3-yl acetate (**7m**)

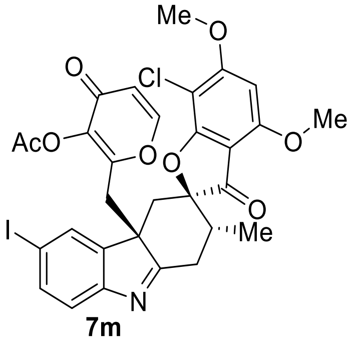

**7m**

Yellow pale solid (59%). **$^{1}$H NMR** (700 MHz, Chloroform-*d*) *δ* 7.66 (dd, *J* = 8.2, 1.6 Hz, 1H), 7.59 (d, *J* = 1.6 Hz, 1H), 7.34 (d, *J* = 5.7 Hz, 1H), 7.30 (d, *J* = 8.2 Hz, 1H), 6.18 (d, *J* = 5.8 Hz, 1H), 6.16 (m, 1H), 4.04 (m, 4H), 3.99 (s, 3H), 3.64 (dd, *J* = 13.1, 5.4 Hz, 1H), 3.21 (d, *J* = 14.5 Hz, 1H), 2.81 (dd, *J* = 13.0, 1.9 Hz, 1H), 2.47 (dd, *J* = 7.2, 5.4 Hz, 1H), 2.34 (dd, *J* = 15.1, 1.9 Hz, 1H), 2.34 (s, 3H), 2.08 (d, *J* = 15.2 Hz, 1H), 0.97 (d, *J* = 7.3 Hz, 3H) ppm. **$^{13}$C NMR** (176 MHz, Chloroform-*d*) *δ* 194.9, 183.6, 171.2, 166.9, 166.7, 164.4, 158.1, 156.6, 153.9, 153.3, 144.2, 139.3, 137.9, 131.8, 122.3, 116.6, 104.3, 97.7, 91.4, 90.5, 89.6, 57.0, 56.5, 56.4, 40.4, 38.9, 34.6, 33.9, 20.6, 13.3 ppm. **HRMS**-ESI (*m/z*): [M + H]$^+$ calculated for $C_{30}H_{26}IClNO_8$ [M+H]$^+$ = 690.0386 found 690.0388.

2-(((2*R*,2'*R*,4a'*S*)-6'-bromo-7-chloro-5',8'-difluoro-4,6-dimethoxy-2'-methyl-3-oxo-1',2'-dihydro-3H-spiro[benzofuran-2,3'-carbazol]-4a'(4'H)-yl)methyl)-4-oxo-4H-pyran-3-yl acetate (**7n**)

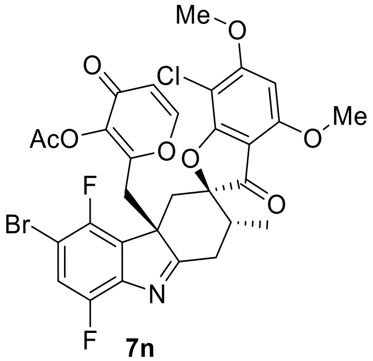


**7n**

Yellow pale solid (47%). **$^{1}$H NMR** (500 MHz, Chloroform-*d*) *δ* 7.46 (d, *J* = 5.8 Hz, 1H), 7.31 (dd, *J* = 8.2, 5.2 Hz, 1H), 6.23 (d, *J* = 5.6 Hz, 1H), 6.16 (s, 1H), 4.10 (d, *J* = 14.5 Hz, 1H), 4.05 (s, 3H), 3.99 (s, 3H), 3.67 (dd, *J* = 13.0, 5.1 Hz, 1H), 3.53 (d, *J* = 14.5 Hz, 1H), 2.85 (dd, *J* = 13.0, 2.1 Hz, 1H), 2.57 (d, *J* = 15.3 Hz, 1H), 2.50 (m, 1H), 2.32 (s, 3H), 2.23(m, 1H), 0.99 (d, *J* = 7.2 Hz, 3H) ppm. **$^{13}$C NMR** (126 MHz, Chloroform-*d*) *δ* 194.7, 184.9, 171.2, 167.0, 166.7, 164.5, 158.2, 156.2, 154.1, 150.4, 141.7, 139.5, 121.5, 121.3, 116.8, 104.3, 97.8, 90.9, 89.6, 89.3, 58.2, 57.1, 56.5, 40.4, 38.0, 34.0, 33.4, 29.7, 20.3, 13.4 ppm. **HRMS**-ESI (*m/z*): [M + H]$^+$ calculated for $C_{33}H_{24}BrClF_2NO_8$ [M+H]$^+$ = 678.0336 found 678.0338. $[\alpha]_D^{20}$ +61 (*c* 0.63, $CHCl_3$).

## Griseofulvin-Indolines (8)

2-(((2R,2'R,4a'S,9a'S)-7-chloro-4,6-dimethoxy-2'-methyl-3-oxo-1',2',9',9a'-tetrahydro-3H-spiro[benzofuran-2,3'-carbazol]-4a'(4'H)-yl)methyl)-4-oxo-4H-pyran-3-yl acetate (**8a**)

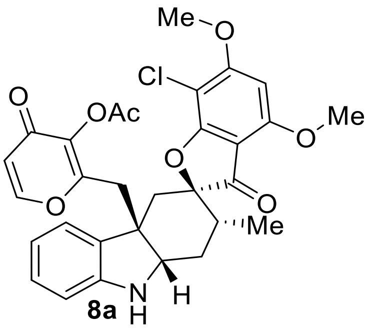


White amorph solid (22 mg, 50%). **$^1$H NMR** (400 MHz, Chloroform-*d*) *δ* 7.36 (d, *J* = 5.7 Hz, 1H), 6.99 (td, *J* = 7.6, 1.3 Hz, 1H), 6.87 (ddd, *J* = 7.5, 1.3, 0.6 Hz, 1H), 6.67 (t, *J* = 7.5 Hz, 1H), 6.59 (d, *J* = 7.9 Hz, 1H), 6.22 (d, *J* = 5.7 Hz, 1H), 6.03 (s, 1H), 3.94 (s, 3H), 3.88 (s, 3H), 3.83 (m, 1H), 3.56 (d, *J* = 14.6 Hz, 1H), 2.90 (d, *J* = 14.6 Hz, 1H), 2.30 (d, *J* = 15.2 Hz, 1H), 2.22 (s, 3H), 2.06 (d, *J* = 9.8 Hz, 1H), 1.98 (m, 1H), 1.85 (m, 2H), 1.02 (d, *J* = 7.0 Hz, 3H) ppm. **$^{13}$C NMR** (176 MHz, Chloroform-*d*) *δ* 196.6, 172.0, 167.5, 167.5, 164.1, 159.5, 158.0, 154.5, 140.6, 133.7, 129.3, 128.5, 126.0, 123.7, 116.7, 110.6, 105.1, 97.4, 92.8, 89.3, 61.5, 57.1, 56.5, 47.7, 38.3, 37.5, 34.5, 32.2, 20.5, 16.2 ppm. **HRMS**-ESI (*m/z*): [M + H]$^+$ calculated for $C_{30}H_{29}ClNO_8$ [M+H]$^+$ = 566.1576 found 566.1575. $[\alpha]_D^{20}$ $-11.3$ (*c* 0.15, $CHCl_3$).

2-(((2R,2'R,4a'S,9a'S)-7-chloro-4,6-dimethoxy-2',6'-dimethyl-3-oxo-1',2',9',9a'-tetrahydro-3H-spiro[benzofuran-2,3'-carbazol]-4a'(4'H)-yl)methyl)-4-oxo-4H-pyran-3-yl acetate (**8b**)

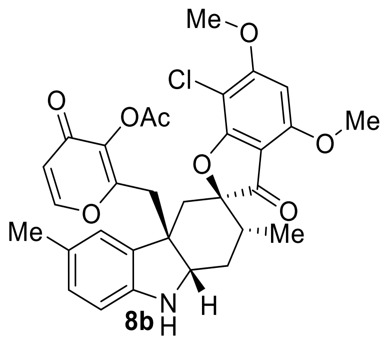

8b

White amorph solid (30 mg, 52%). **$^{1}$H NMR** (700 MHz, Methanol-$d_4$) $\delta$ 7.82 (d, $J$ = 5.6 Hz, 1H), 6.82 (d, $J$ = 7.9 Hz, 1H), 6.75 (s, 1H), 6.51 (d, $J$ = 7.6 Hz, 1H), 6.36 (m, 2H), 4.03 (s, 3H), 3.93 (s, 3H), 3.77 (t, $J$ = 7.0 Hz, 1H), 3.44 (d, $J$ = 14.4 Hz, 1H), 3.04 (d, $J$ = 14.4 Hz, 1H), 2.33 (d, $J$ = 15.2 Hz, 1H), 2.21 (s, 3H), 2.19 (s, 3H), 2.15 (m, 1H), 2.08 (d, $J$ = 15.1 Hz, 1H), 1.92 (t, $J$ = 7.1 Hz, 2H), 0.96 (d, $J$ = 7.0 Hz, 3H) ppm. **$^{13}$C NMR** (176 MHz, Methanol-$d_4$) $\delta$ 198.7, 174.5, 168.9 (d, $J$ = 11.4 Hz), 166.2, 162.5, 159.5, 157.4, 148.3, 141.1, 134.6, 129.7, 129.4, 128.9, 125.3, 116.9, 111.2, 105.9, 97.7, 94.1, 91.0, 63.5, 57.7, 56.8, 49.6, 39.3, 35.9, 33.6, 21.1, 20.3, 16.1 ppm. **HRMS**-ESI ($m/z$): [M + H]$^+$ calculated for $C_{31}H_{31}ClNO_8$ [M+H]$^+$ = 580.1733 found 580.1729. $[\alpha]_D^{20}$ +12.7 ($c$ 0.1, $CHCl_3$).

2-(((2R,2'R,4a'S,9a'S)-7-chloro-6'-isopropyl-4,6-dimethoxy-2'-methyl-3-oxo-1',2',9',9a'-tetrahydro-3H-spiro[benzofuran-2,3'-carbazol]-4a'(4'H)-yl)methyl)-4-oxo-4H-pyran-3-yl acetate (**8d**)

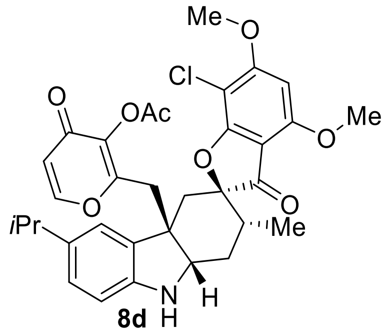

8d

White amorph solid (18 mg, 21%). **$^{1}$H NMR** (700 MHz, Methanol-$d_4$) $\delta$ 7.79 (d, $J$ = 5.5 Hz, 1H), 6.87 (d, $J$ = 7.9 Hz, 1H), 6.77 (s, 1H), 6.55 (d, $J$ = 5.6 Hz, 1H), 6.36 (s, 1H), 6.34 (d, $J$ = 5.6 Hz, 1H), 4.03 (s, 3H), 3.92 (s, 3H), 3.77 (t, $J$ = 6.9 Hz, 1H), 3.42 (d, $J$ = 14.3 Hz, 1H), 3.06 (d, $J$ = 14.3 Hz, 1H), 2.74 (p, $J$ = 6.8 Hz, 1H), 2.34 (d, $J$ = 15.0 Hz, 1H), 2.19 (s, 3H), 2.15 (q, $J$ = 7.1 Hz, 1H), 2.11 (d, $J$ = 15.0 Hz, 1H), 1.94 (t, $J$ = 6.9 Hz, 2H), 1.14 (d, $J$ = 6.8 Hz, 6H), 0.97 (d, $J$ = 7.0 Hz,

3H) ppm. $^{13}$**C NMR** (176 MHz, Methanol-$d_4$) $\delta$ 198.6, 174.5, 169.0, 168.7, 166.1, 162.5, 159.6, 157.4, 148.5, 141.1, 140.6, 134.2, 127.2, 122.6, 116.9, 111.1, 105.9, 97.8, 94.1, 91.0, 63.9, 57.7, 56.8, 49.7, 39.2, 39.0, 35.9, 35.0, 33.7, 24.9, 24.9, 20.3, 16.1 ppm. **HRMS**-ESI (*m/z*): $[M + H]^+$ calculated for $C_{33}H_{35}ClNO_8$ $[M+H]^+$ = 608.2046 found 608.2043. $[\alpha]_D^{20}$ +5.9 (*c* 0.17, $CHCl_3$).

2-(((2R,2'R,4a'S,9a'S)-7-chloro-4,6-dimethoxy-2'-methyl-3-oxo-6'-(trifluoromethoxy)-1',2',9',9a'-tetrahydro-3H-spiro[benzofuran-2,3'-carbazol]-4a'(4'H)-yl)methyl)-4-oxo-4H-pyran-3-yl acetate (**8i**)

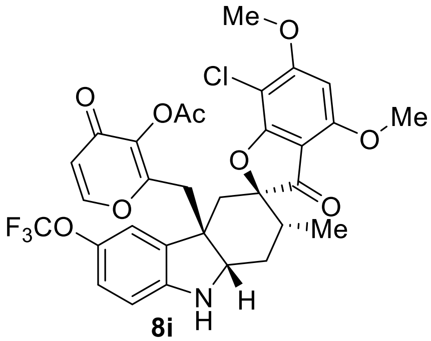


White amorph solid (12 mg, 30%). $^{1}$**H NMR** (700 MHz, Methanol-$d_4$) $\delta$ 7.80 (d, $J$ = 5.7 Hz, 1H), 6.90 (d, $J$ = 8.8 Hz, 1H), 6.88 (s, 1H), 6.58 (d, $J$ = 8.4 Hz, 1H), 6.36 (m, 2H), 4.03 (s, 3H), 3.92 (s, 3H), 3.81 (t, $J$ = 6.7 Hz, 1H), 3.48 (d, $J$ = 14.4 Hz, 1H), 3.02 (d, $J$ = 14.5 Hz, 1H), 2.37 (d, $J$ = 15.1 Hz, 1H), 2.23 (s, 3H), 2.13 (m, 2H), 1.96 (m, 2H), 0.97 (d, $J$ = 7.0 Hz, 3H) ppm. $^{13}$**C NMR** (176 MHz, Methanol-$d_4$) $\delta$ 198.5, 174.4, 168.9, 168.8, 166.2, 161.9, 159.6, 157.3, 149.9, 142.4, 141.2, 135.4, 122.5, 118.9, 117.0, 110.6, 105.9, 97.7, 93.6, 91.0, 63.9, 57.7, 56.8, 49.8, 38.9, 38.5, 35.9, 33.2, 20.3, 16.0 ppm. **HRMS**-ESI (*m/z*): $[M + H]^+$ calculated for $C_{31}H_{28}ClF_3NO_9$ $[M+H]^+$ = 650.1399 found 650.1397. $[\alpha]_D^{20}$ +5.0 (*c* 0.12, $CHCl_3$).

2-(((2R,2'R,4a'S,9a'S)-7-chloro-4,6-dimethoxy-2'-methyl-3-oxo-6'-(trifluoromethyl)-1',2',9',9a'-tetrahydro-3H-spiro[benzofuran-2,3'-carbazol]-4a'(4'H)-yl)methyl)-4-oxo-4H-pyran-3-yl acetate (**8j**)

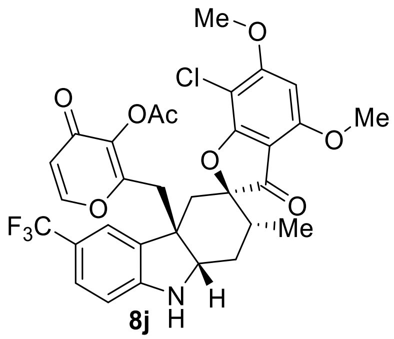

**8j**

White amorph solid (22 mg, 31%). **$^{1}$H NMR** (700 MHz, Methanol-$d_4$) $\delta$ 7.81 (d, $J$ = 5.5 Hz, 1H), 7.26 (d, $J$ = 8.2 Hz, 1H), 7.23 (s, 1H), 6.60 (d, $J$ = 8.2 Hz, 1H), 6.36 (m, 2H), 4.03 (s, 3H), 3.92 (s, 3H), 3.89 (m, 1H), 3.46 (d, $J$ = 14.5 Hz, 1H), 3.09 (d, $J$ = 14.5 Hz, 1H), 2.36 (d, $J$ = 15.1 Hz, 1H), 2.23 (s, 3H), 2.14 (m, 2H), 2.01 (d, $J$ = 6.0 Hz, 1H), 1.94 (dt, $J$ = 17.6, 11.4 Hz, 2H), 0.97 (d, $J$ = 6.9 Hz, 3H) ppm. **$^{13}$C NMR** (176 MHz, Methanol-$d_4$) $\delta$ 196.4, 172.2, 166.8, 166.7, 164.1, 159.8, 157.5, 155.2, 139.0, 132.2, 125.0, 120.0, 118.2, 114.9, 107.2, 103.7, 95.6, 91.5, 88.9, 61.2, 55.6, 54.7, 47.1, 36.9, 36.8, 33.6, 31.3, 25.8, 18.2, 13.9 ppm. **HRMS**-ESI (*m/z*): $[M + H]^+$ calculated for $C_{31}H_{28}ClF_3NO_8$ $[M+H]^+$ = 634.1450 found 634.1448. $[\alpha]_D^{20}$ +1.3 (*c* 0.15, $CHCl_3$).

2-(((2R,2'R,4a'S,9a'S)-6',7-dichloro-4,6-dimethoxy-2'-methyl-3-oxo-1',2',9',9a'-tetrahydro-3H-spiro[benzofuran-2,3'-carbazol]-4a'(4'H)-yl)methyl)-4-oxo-4H-pyran-3-yl acetate (**8l**)

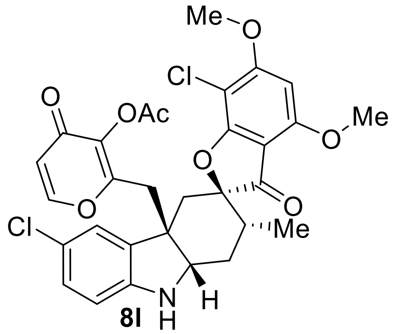

**8l**

White amorph solid (5 mg, 7%). **$^{1}$H NMR** (700 MHz, Methanol-$d_4$) $\delta$ 7.87 (d, $J$ = 5.6 Hz, 1H), 6.96 (m, 2H), 6.53 (d, $J$ = 8.7 Hz, 1H), 6.38 (d, $J$ = 5.7 Hz, 1H), 6.37 (s, 1H), 4.03 (s, 3H), 3.93 (s, 3H), 3.80 (t, $J$ = 6.9 Hz, 1H), 3.43 (d, $J$ = 14.4 Hz, 1H), 3.03 (d, $J$ = 14.5 Hz, 1H), 2.36 (d, $J$ = 15.1 Hz, 1H), 2.24 (s, 3H), 2.15 (m, 2H), 1.93 (p, $J$ = 9.5 Hz, 2H), 0.95 (d, $J$ = 6.9 Hz, 3H) ppm. **$^{13}$C NMR** (176 MHz, Methanol-$d_4$) $\delta$ 198.6, 174.5, 168.9, 168.8, 166.2, 162.0, 159.6, 157.5, 149.6, 141.2, 136.1, 129.0, 125.0, 123.6, 117.0, 111.6, 105.9, 97.7, 93.7, 91.0, 63.5, 57.7, 56.8, 49.8, 39.3,

38.8, 35.9, 33.4, 20.4, 16.0 ppm. **HRMS**-ESI (*m/z*): $[M + H]^+$ calculated for $C_{30}H_{28}Cl_2NO_8$ $[M+H]^+$ = 600.1186 found 600.1185.

2-(((2R,2'R,4a'S,9a'S)-7-chloro-6'-iodo-4,6-dimethoxy-2'-methyl-3-oxo-1',2',9',9a'-tetrahydro-3H-spiro[benzofuran-2,3'-carbazol]-4a'(4'H)-yl)methyl)-4-oxo-4H-pyran-3-yl acetate (**8m**)

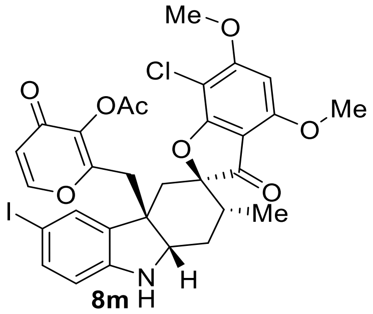


White amorph solid (19 mg, 25%). **$^1$H NMR** (700 MHz, Methanol-$d_4$) $\delta$ 7.87 (m, 1H), 7.27 (d, $J$ = 8.3 Hz, 1H), 7.24 (s, 1H), 6.39 (m, 3H), 4.03 (s, 3H), 3.94 (s, 3H), 3.79 (t, $J$ = 6.8 Hz, 1H), 3.42 (d, $J$ = 14.5 Hz, 1H), 3.02 (d, $J$ = 14.4 Hz, 1H), 2.34 (d, $J$ = 15.1 Hz, 1H), 2.25 (s, 3H), 2.15 (m, 1H), 2.09 (d, $J$ = 15.1 Hz, 1H), 1.93 (t, $J$ = 6.8 Hz, 2H), 0.96 (d, $J$ = 6.9 Hz, 3H) ppm. **$^{13}$C NMR** (176 MHz, Methanol-$d_4$) $\delta$ 198.6, 174.4, 169.0, 168.8, 166.2, 162.0, 159.6, 157.4, 150.7, 141.2, 138.0, 137.1, 133.7, 117.0, 112.8, 105.9, 97.8, 93.8, 91.1, 78.8, 63.3, 57.7, 56.9, 49.7, 39.3, 38.9, 35.8, 33.4, 20.5, 16.0 ppm. **HRMS**-ESI (*m/z*): $[M + H]^+$ calculated for $C_{30}H_{28}ClINO_8$ $[M+H]^+$ = 692.0543 found 692.0541. $[\alpha]_D^{20}$ +54.5 (*c* 0.044 $CHCl_3$).

## Synthesis of GF-THPI-β (9), GF-THPI-γ (10) and GF-Chromanones (11)

The compounds of compound classes **9**, **10**, and **11** were synthesized according to those previously published and the spectral data agree with those.[4,5,7]

## Structural Determination of 8j

Methylene hydrogens (2.87 ppm / 3.61 ppm) and methyne hydrogens (2.04 ppm and 3.86 ppm) could be assigned by 1H, 13C, COSY, HSQC, and HMBC NMR analysis. NOESY NMR showed correlation between 3.86 ppm and 2.04 ppm as well as 3.86 ppm with both 2.87 ppm and 3.61 ppm. These observations led to the conclusion that the newly formed stereocenter after imine reduction has the (*S*) configuration. The (*S*) configuration was analogously assigned to similar indoline products (**8**).

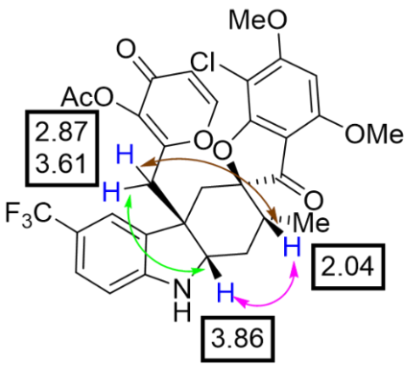


**8j**

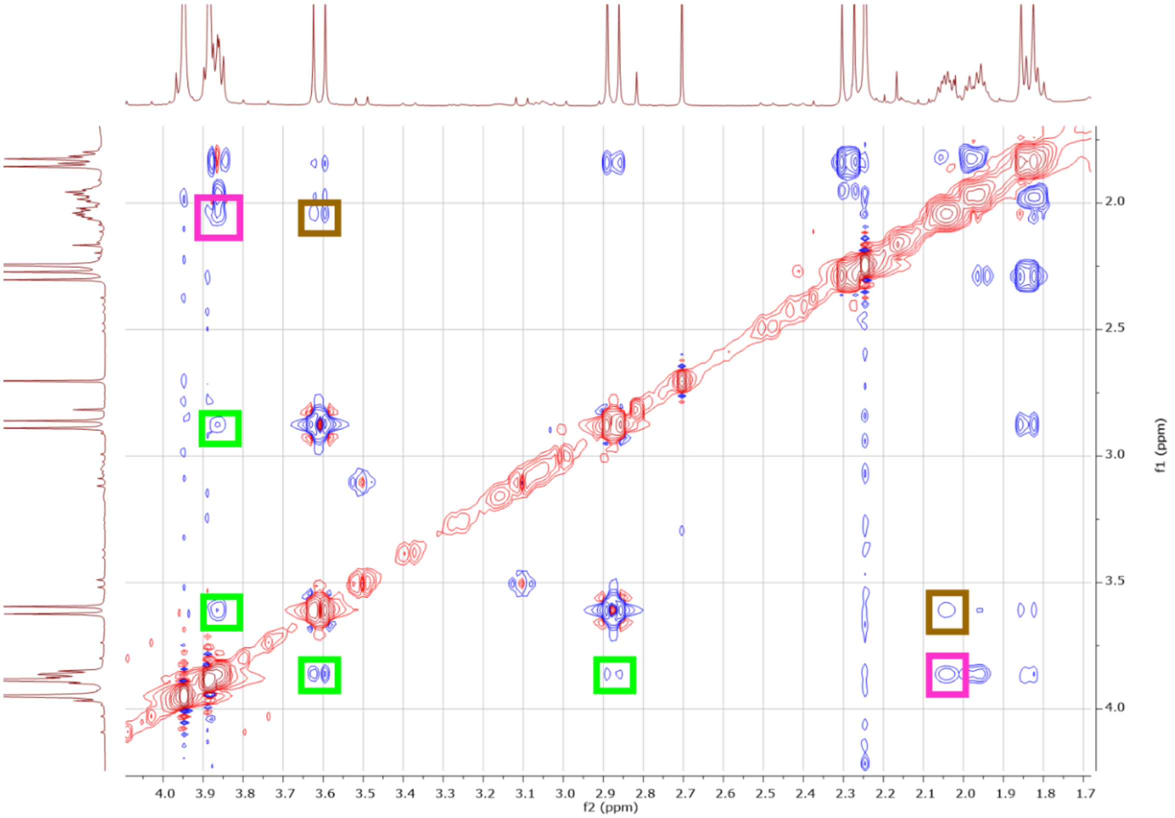

# X-Ray Crystallography Data

## X-ray analysis of compound 3b

Crystals of compound **3b** suitable for x-ray analysis were obtained by vapor diffusion experiments from THF and *iso*-hexane at -30 °C.

The crystal structure of compound **3b** was determined using the *Bruker D8 Venture* four-circle diffractometer equipped with a *PHOTON II* CPAD detector by *Bruker AXS GmbH*. The X-ray radiation was generated by the *IµS/IµS* microfocus source Mo (λ = 0.71073 Å) from *Incoatec GmbH* equipped with HELIOS mirror optics and a single-hole collimator by *Bruker AXS GmbH*. The selected single crystal of **3b** was covered with an inert oil (perfluoropolyalkyl ether) and mounted on the *MicroMount* from *MiTeGen*. The APEX 3 Suite (v.2018.7-2) software integrated with SAINT (integration) and SADABS (adsorption correction) programs by *Bruker AXS GmbH* were used for data collection. The processing and finalization of the crystal structure were performed using the Olex2 program.[8] The crystal structures were solved by the ShelXT[9] structure solution program using the Intrinsic Phasing option, which were further refined by the ShelXL[10] refinement package using Least Squares minimization. The non-hydrogen atoms were anisotropically refined. The C-bound H atoms were placed in geometrically calculated positions, and a fixed isotropic displacement parameter was assigned to each atom according to the riding-model: C–H = 0.95–1.00 Å with $U_{iso}$(H) = –1.2$U_{eq}$($CH_2$, CH) and $U_{iso}$(H) = –1.5$U_{eq}$($CH_3$)for other hydrogen atoms. The N-bound hydrogen atom was placed free. The crystallographic data for the structure of **3b** has been published as supplementary publication number 2417169 (**3b**) in the Cambridge Crystallographic Data Centre. A copy of these data can be obtained for free by applying to CCDC, 12 Union Road, Cambridge CB2 IEZ, UK, fax: 144-(0)1223-336033 or e-mail: deposit@ccdc.cam.ac.uk.

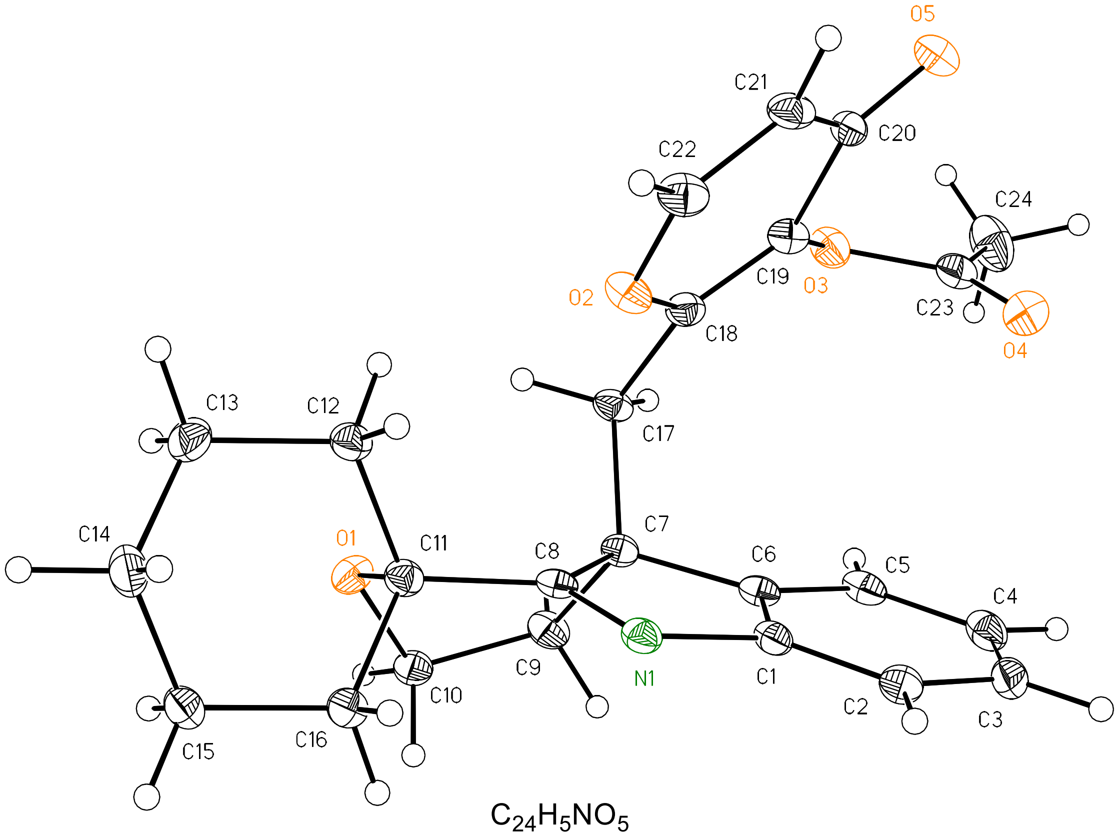


$C_{24}H_5NO_5$
**3b**

Figure S 5: Ortep plot of the molecular structure in the crystal of compound **3b**.[11] The displacement ellipsoids are drawn at 50% probability level. Numbering scheme of hydrogen atoms are omitted for clarity.

Table S 2**:** Crystallographic data of compound **3b**.

| Compound | **3b** |
|---|---|
| Empirical formula | $C_{24}H_{25}NO_5$ |
| Formula weight | 407.45 |
| Temperature/K | 100.0 |
| Crystal system | monoclinic |
| Space group | $P2_1/c$ |
| a/Å | 13.8184(7) |
| b/Å | 18.8796(8) |
| c/Å | 7.6626(4) |
| α/° | 90 |
| β/° | 98.071(2) |
| γ/° | 90 |
| Volume/Å$^3$ | 1979.26(17) |
| Z | 4 |
| $\rho_{calc}$g/cm$^3$ | 1.367 |
| μ/mm$^{-1}$ | 0.096 |
| F(000) | 864.0 |
| Crystal size/mm$^3$ | 0.439 × 0.132 × 0.054 |
| Radiation | MoKα (λ = 0.71073) |
| 2Θ range for data collection/° | 4.314 to 54.254 |
| Index ranges | $-17 \le h \le 17$, $0 \le k \le 24$, $-9 \le l \le 0$ |
| Reflections collected | 4339 |
| Independent reflections | 4339 [$R_{int}$ = 0.0365, $R_{sigma}$ = 0.0438] |
| Data/restraints/parameters | 4339/0/272 |
| Goodness-of-fit on $F^2$ | 1.074 |
| Final R indexes [I>=2σ (I)] | $R_1$ = 0.0610, w$R_2$ = 0.1250 |
| Final R indexes [all data] | $R_1$ = 0.0831, $wR_2$ = 0.1331 |
| Largest diff. peak/hole / e Å$^{-3}$ | 0.36/–0.30 |
| | |

## X-ray analysis of compound 7k

Crystals of compound **7k** suitable for x-ray analysis were obtained by vapor diffusion experiments from THF and *n*-pentane at 4 °C. The newly generated stereocenter was determined to have the (*S*) configuration and was assumed to be analogous for similar products (**7**).

The crystal structure of compound **7k** was determined using the *Bruker D8 Venture* four-circle diffractometer equipped with a *PHOTON II* CPAD detector by *Bruker AXS GmbH*. The X-ray radiation was generated by the *IμS/IμS* microfocus source Mo ($\lambda$ = 0.71073 Å) from *Incoatec GmbH* equipped with HELIOS mirror optics and a single-hole collimator by *Bruker AXS GmbH*. The selected single crystal of **7k** was covered with an inert oil (perfluoropolyalkyl ether) and mounted on the *MicroMount* from *MiTeGen*. The APEX 3 Suite (v.2018.7-2) software integrated with SAINT (integration) and SADABS (adsorption correction) programs by *Bruker AXS GmbH* were used for data collection. The processing and finalization of the crystal structure were performed using the Olex2 program.[8] The crystal structures were solved by the ShelXT[9] structure solution program using the Intrinsic Phasing option, which were further refined by the ShelXL[10] refinement package using Least Squares minimization. The non-hydrogen atoms were anisotropically refined. The C-bound H atoms were placed in geometrically calculated positions, and a fixed isotropic displacement parameter was assigned to each atom according to the riding-model: C–H = 0.95–1.00 Å with $U_{iso}$(H) = –1.2$U_{eq}$($CH_2$, CH) and $U_{iso}$(H) = –1.5$U_{eq}$($CH_3$)for other hydrogen atoms. The N-bound hydrogen atom was placed free. The crystallographic data for the structure of **7k** has been published as supplementary publication number 2417170 (**7k**) in the Cambridge Crystallographic Data Centre. A copy of these data can be obtained for free by applying to CCDC, 12 Union Road, Cambridge CB2 IEZ, UK, fax: 144-(0)1223-336033 or e-mail: deposit@ccdc.cam.ac.uk.

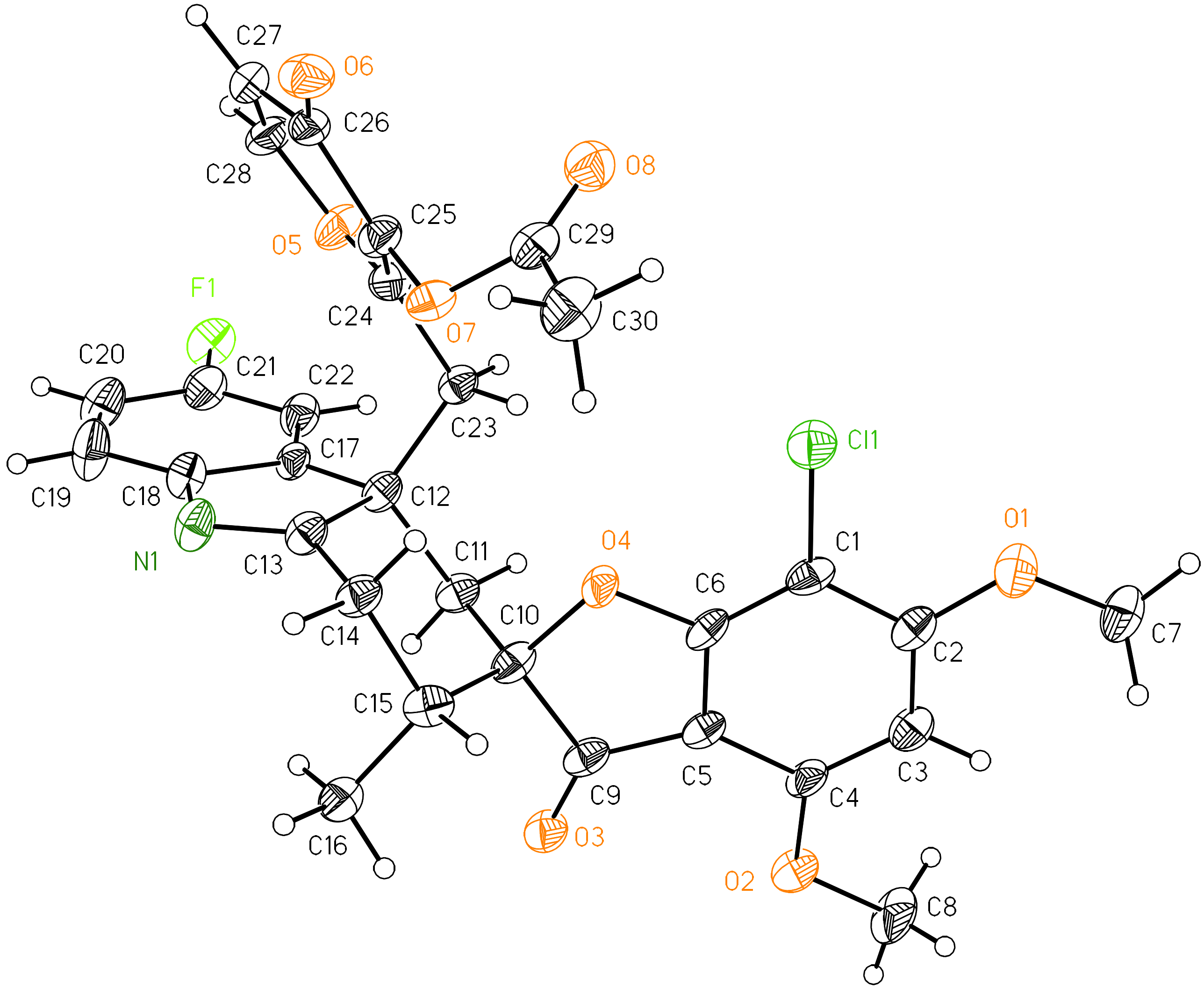


$C_{30}H_{25}ClFNO_8$
**7k**

Figure S 6: Ortep plot of the molecular structure in the crystal of compound **7k**.[11] The displacement ellipsoids are drawn at 50% probability level. Numbering scheme of hydrogen atoms are omitted for clarity.

Table S 3: Crystallographic data of compound **7k**.

| Compound | **8k** |
|---|---|
| Empirical formula | $C_{30}H_{25}ClFNO_8$ |
| Formula weight | 581.96 |
| Temperature/K | 100.0 |
| Crystal system | orthorhombic |
| Space group | $P2_12_12$ |
| a/Å | 25.688(8) |
| b/Å | 10.879(3) |
| c/Å | 11.142(4) |
| α/° | 90 |
| β/° | 90 |
| γ/° | 90 |
| Volume/Å$^3$ | 3113.7(17) |
| Z | 4 |
| $\rho_{calc}$g/cm$^3$ | 1.241 |
| μ/mm$^{-1}$ | 0.176 |
| F(000) | 1208.0 |
| Crystal size/mm$^3$ | 0.160 × 0.117 × 0.086 |
| Radiation | MoKα (λ = 0.71073) |
| 2Θ range for data collection/° | 4.84 to 55.928 |
| Index ranges | –33 ≤ h ≤ 33,<br>–14 ≤ k ≤ 14,<br>–14 ≤ l ≤ 14 |
| Reflections collected | 27147 |
| Independent reflections | 7433 [$R_{int}$ = 0.0943, $R_{sigma}$ = 0.0971] |
| Data/restraints/parameters | 7433/0/374 |
| Goodness-of-fit on F$^2$ | 0.987 |
| Final R indexes [I>=2σ (I)] | $R_1$ = 0.0635,<br>w$R_2$ = 0.1505 |
| Final R indexes [all data] | $R_1$ = 0.0992,<br>$wR_2$ = 0.1666 |
| Largest diff. peak/hole / e Å$^{-3}$ | 0.30/–0.28 |
| Flack parameter | 0.02(7) |

# References


[1] M. A. Bray, S. Singh, H. Han, C. T. Davis, B. Borgeson, C. Hartland, M. Kost-Alimova, S. M. Gustafsdottir, C. C. Gibson, A. E. Carpenter, *Nat. Protoc.* **2016**, *11*, 1757.

[2] M. H. Woehrmann, W. M. Bray, J. K. Durbin, S. C. Nisam, A. K. Michael, E. Glassey, J. M. Stuart, R. S. Lokey, *Mol. BioSyst.* **2013**, *9*, 2604.

[3] A. Pahl, J. Liu, S. Patil, S. Rezaei Adariani, B. Schölermann, J. Warmers, J. Bonowski, S. Koska, Y. Akbulut, C. Seitz, S. Sievers, S. Ziegler, H. Waldmann, *J. Med. Chem.* **2024**, *67*, 8862–8876.

[4] M. Grigalunas, A. Burhop, S. Zinken, A. Pahl, J.-M. Gally, N. Wild, Y. Mantel, S. Sievers, D. J. Foley, R. Scheel, et al., *Nat. Commun.* **2021**, *12*, 1883.

[5] A. Burhop, S. Bag, M. Grigalunas, S. Woitalla, P. Bodenbinder, L. Brieger, C. Strohmann, A. Pahl, S. Sievers, H. Waldmann, *Adv. Sci.* **2021**, *8*, 2102042.

[6] C. S. Yeung, R. E. Ziegler, J. A. Porco and E. N. Jacobsen, *J. Am. Chem. Soc.*, **2014**, *136*, 13614–13617.

[7] M. Grigalunas, S. Patil, A. Krzyzanowski, A. Pahl, J. Flegel, B. Schölermann, J. Xie, S. Sievers, S. Ziegler, H. Waldmann, *Chem. Eur. J.* **2022**, *28*, e202202164.

[8] O. V. Dolomanov, L. J. Bourhis, R. J. Gildea, J. A. K. Howard, H. Puschmann, *J. Appl. Cryst.* **2009**, *42*, 339.

[9] G. M. Sheldrick, *Acta Cryst.* **2015**, *A71*, 3.

[10] G. M. Sheldrick, *Acta Cryst.* **2008**, *A64*, 112.

[11] L. J. Farrugia, *J. Appl. Cryst.* **1997**, *30*, 565.

# NMR Spectra

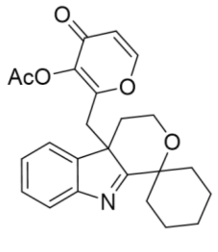


**3b**

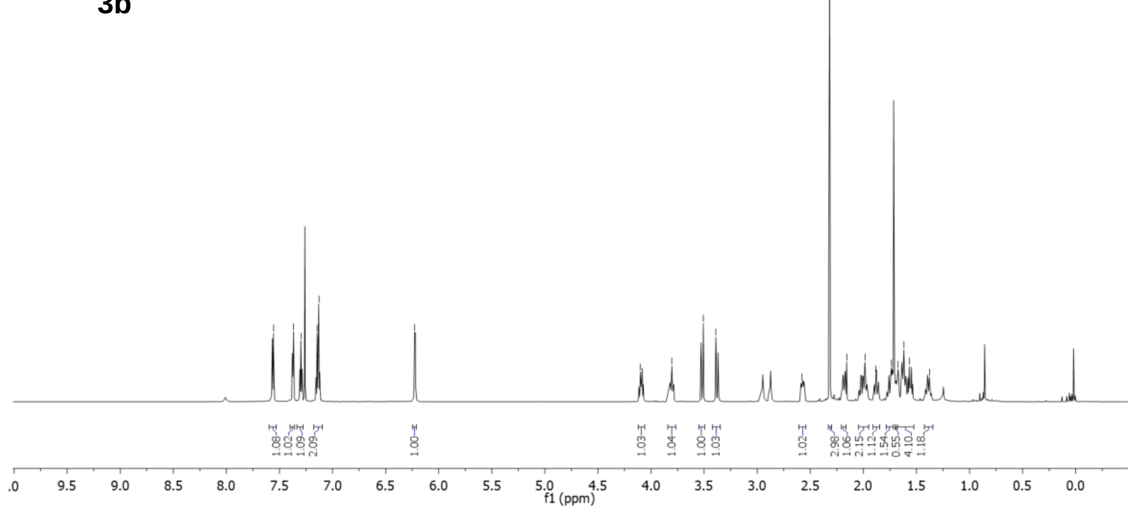


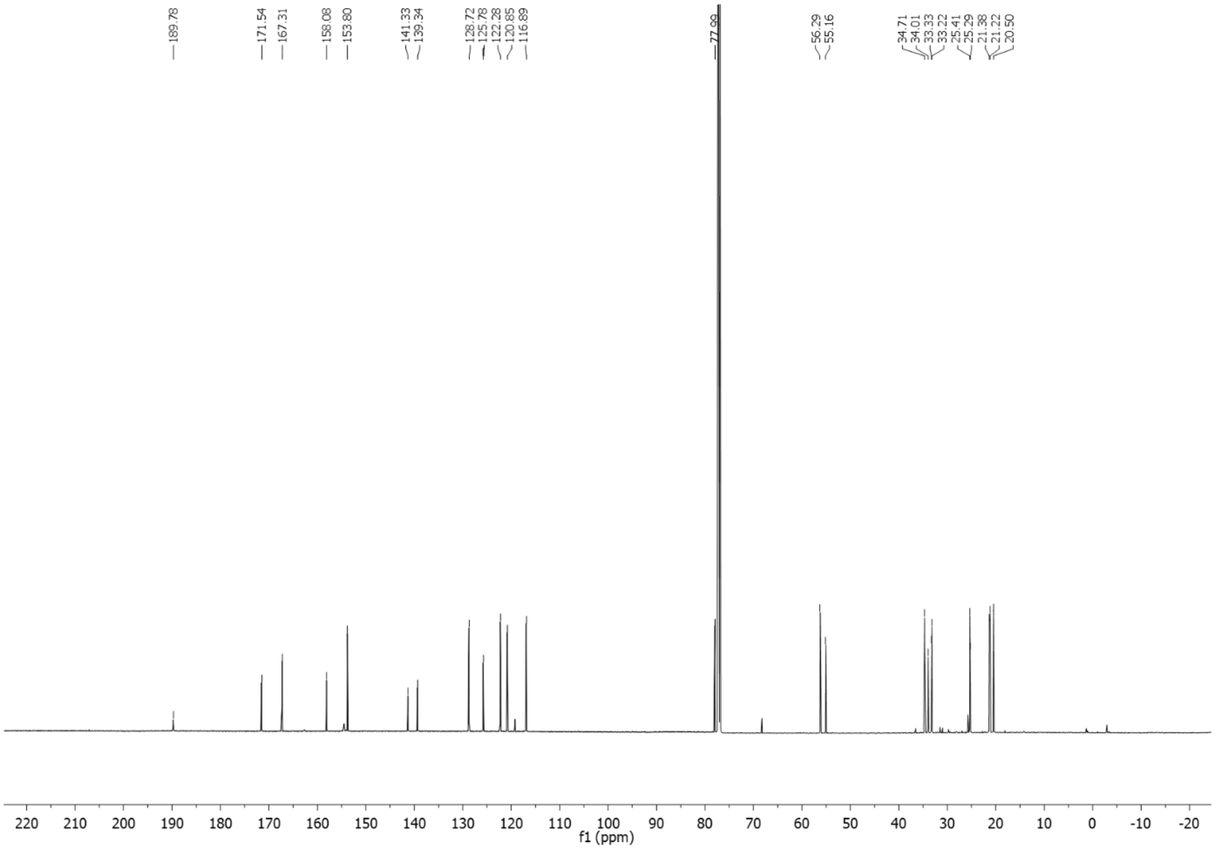

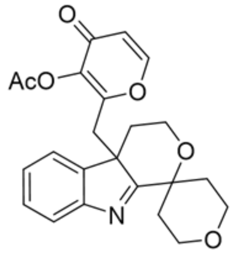
O
AcO
O
O
N
O

**3c**

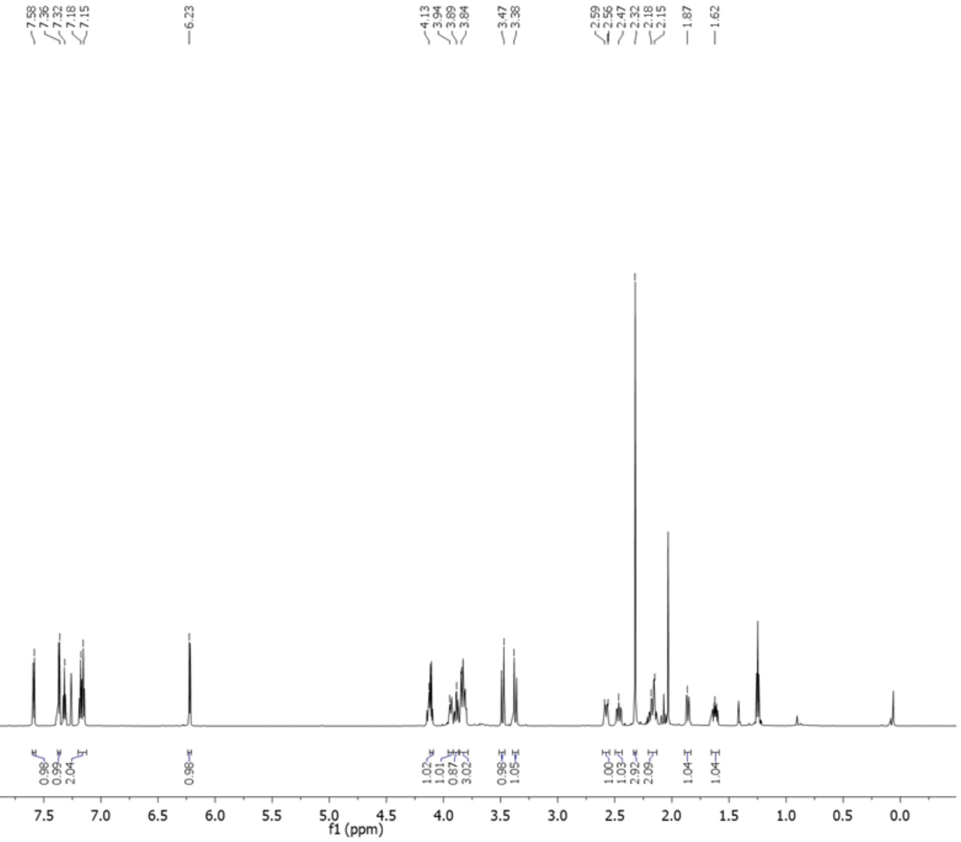
7.58
7.36
7.32
7.18
7.15
6.23
4.13
3.94
3.89
3.84
3.47
3.38
2.59
2.56
2.47
2.32
2.18
2.15
1.87
1.62
0.98
0.99
2.04
0.98
1.02
1.01
0.87
3.02
0.98
1.05
1.00
1.03
2.92
2.09
1.04
1.04
f1 (ppm)

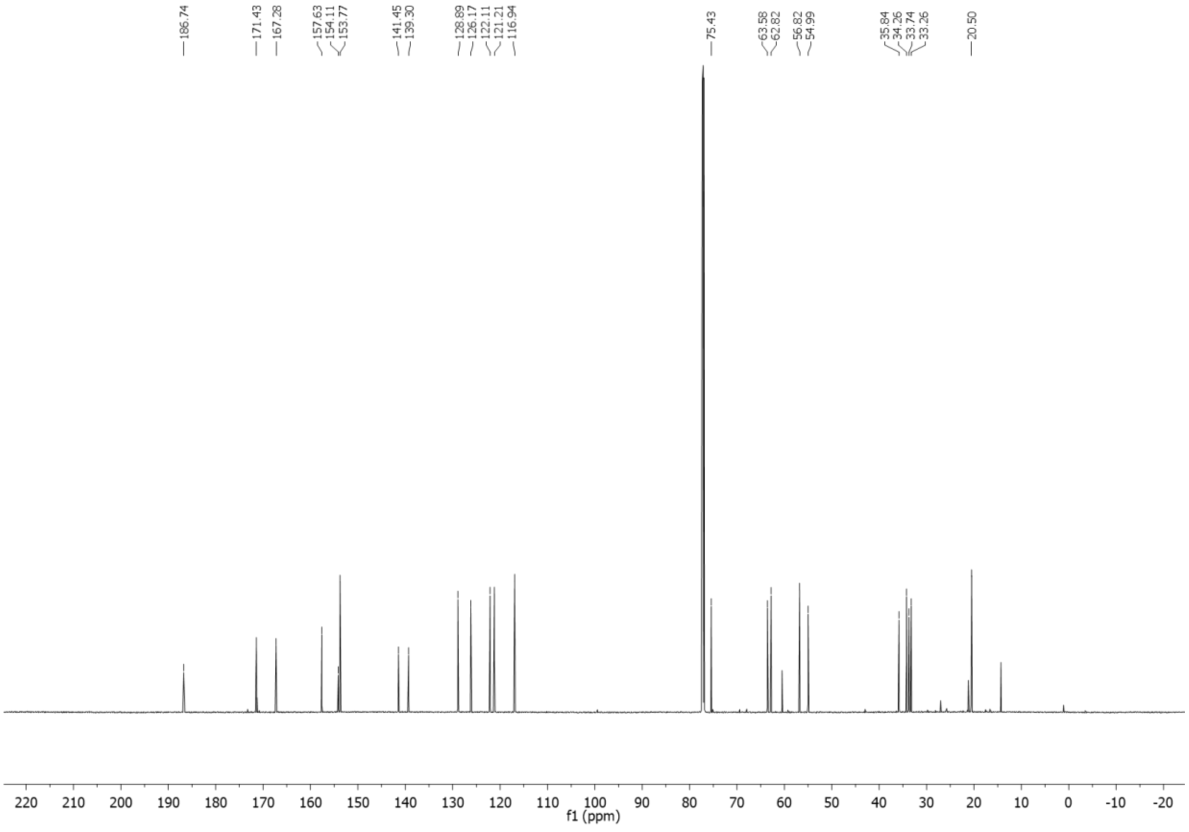
186.74
171.43
167.28
157.63
154.11
153.77
141.45
139.30
128.89
126.17
122.11
121.21
116.94
75.43
63.58
62.82
56.82
54.99
35.84
34.26
33.74
33.26
20.50
f1 (ppm)

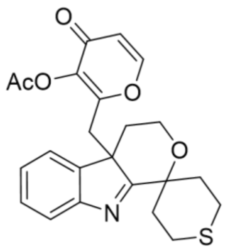
O
AcO
O
O
N
S

**3d**

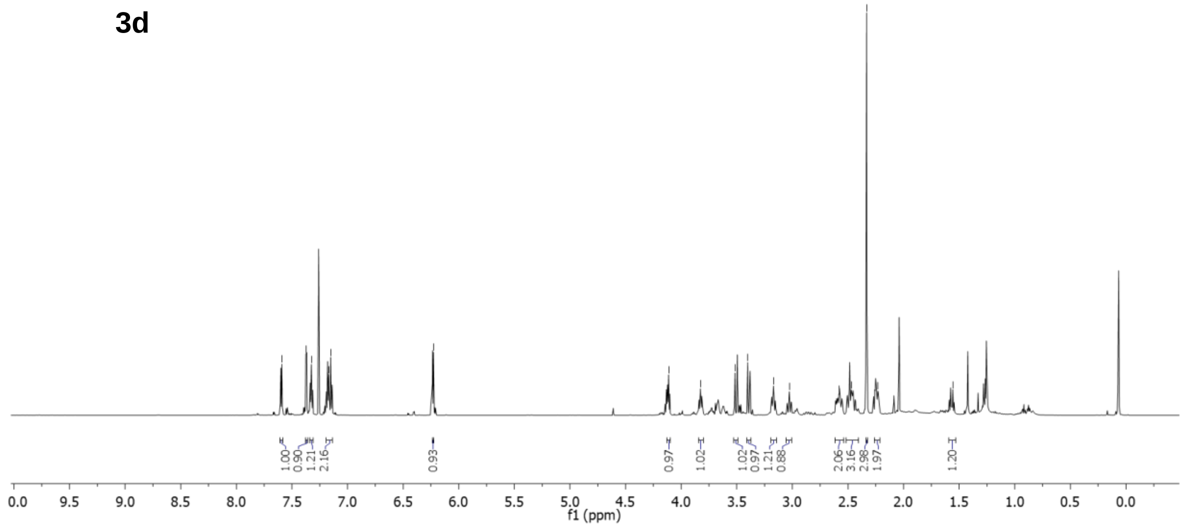
7.59
7.38
7.33
7.17
7.15
6.23
4.11
3.83
3.52
3.40
3.17
3.03
2.58
2.47
2.33
2.23
1.56
1.00
0.90
1.21
2.16
0.93
0.97
1.02
1.02
0.97
1.21
0.88
2.06
3.16
2.98
1.97
1.20
0.0 9.5 9.0 8.5 8.0 7.5 7.0 6.5 6.0 5.5 5.0 4.5 4.0 3.5 3.0 2.5 2.0 1.5 1.0 0.5 0.0
f1 (ppm)

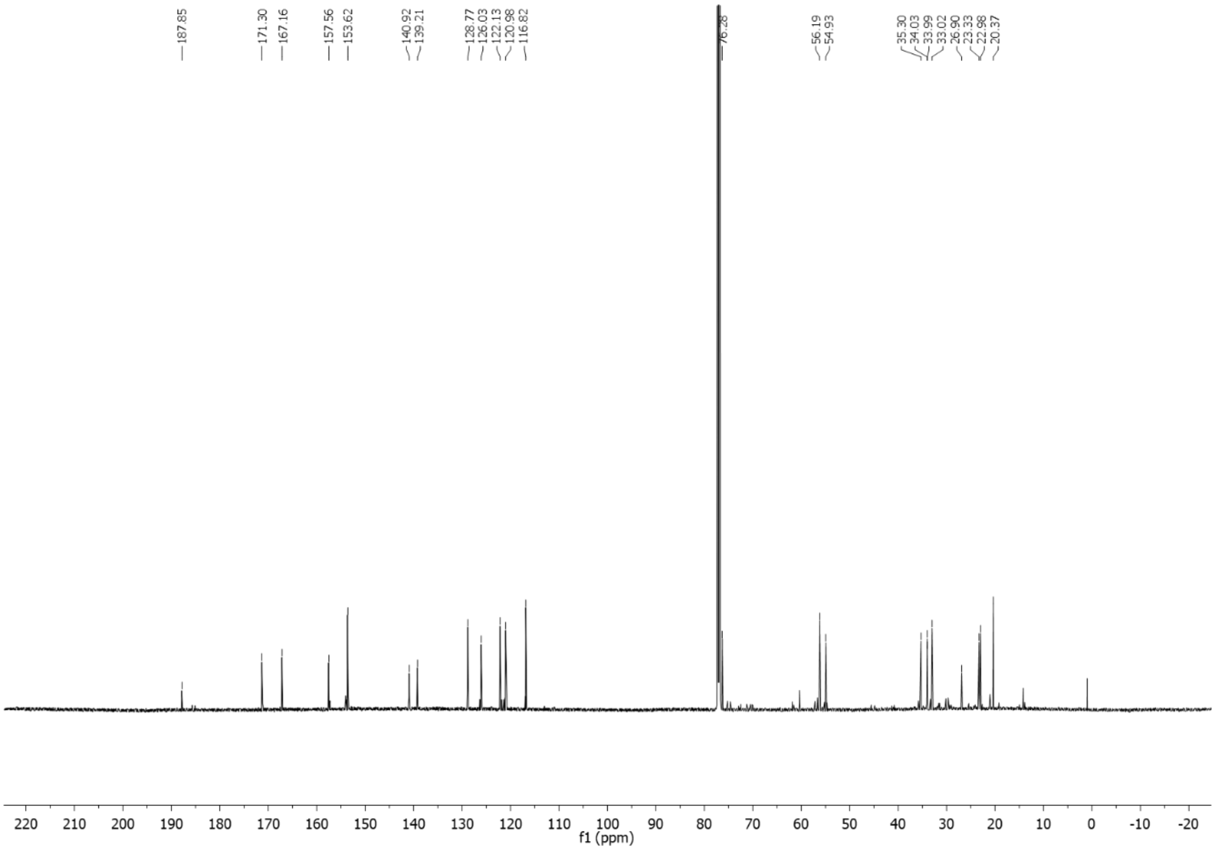
187.85
171.30
167.16
157.56
153.62
140.92
139.21
128.77
126.03
122.13
120.98
116.82
76.28
56.19
54.93
35.30
34.03
33.99
33.02
26.90
23.33
22.98
20.37
220 210 200 190 180 170 160 150 140 130 120 110 100 90 80 70 60 50 40 30 20 10 0 -10 -20
f1 (ppm)

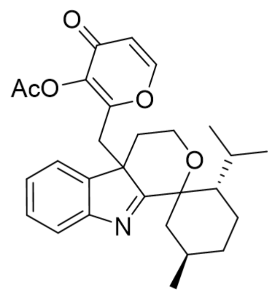
O
AcO
O
O
N

**3e**

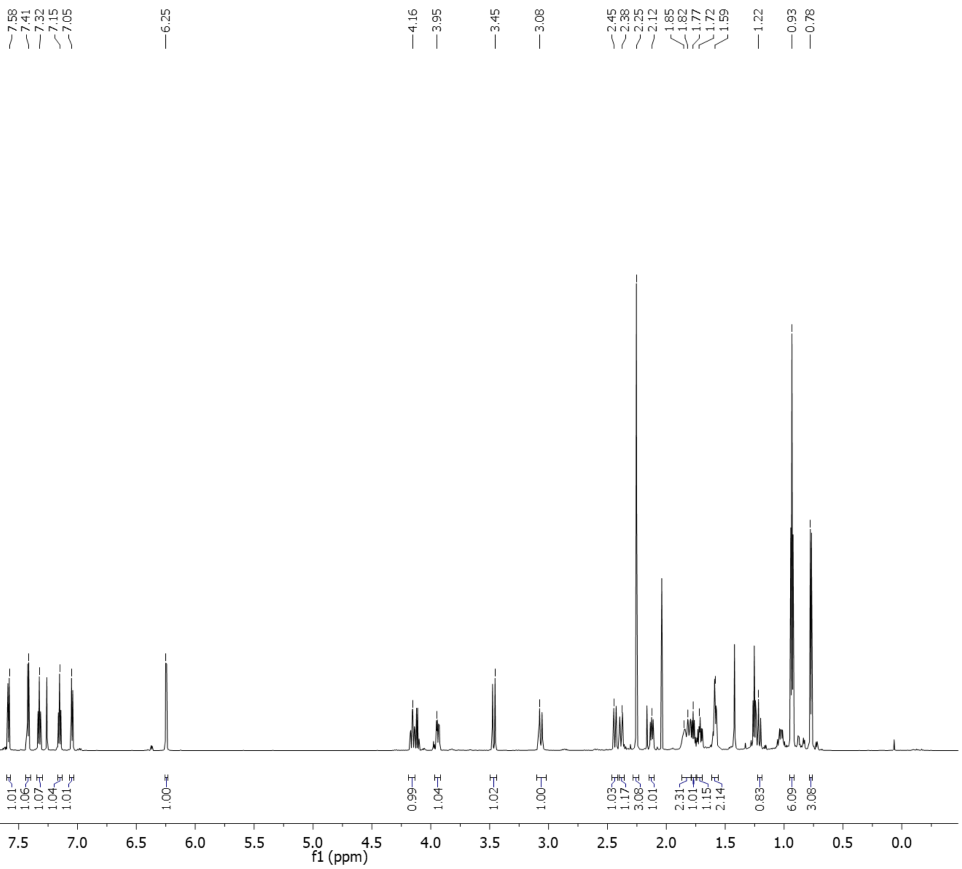
7.58
7.41
7.32
7.15
7.05
6.25
4.16
3.95
3.45
3.08
2.45
2.38
2.25
2.12
1.85
1.82
1.77
1.72
1.59
1.22
0.93
0.78
1.01
1.06
1.07
1.04
1.01
1.00
0.99
1.04
1.02
1.00
1.03
1.17
3.08
1.01
2.31
1.01
1.15
2.14
0.83
6.09
3.08
f1 (ppm)

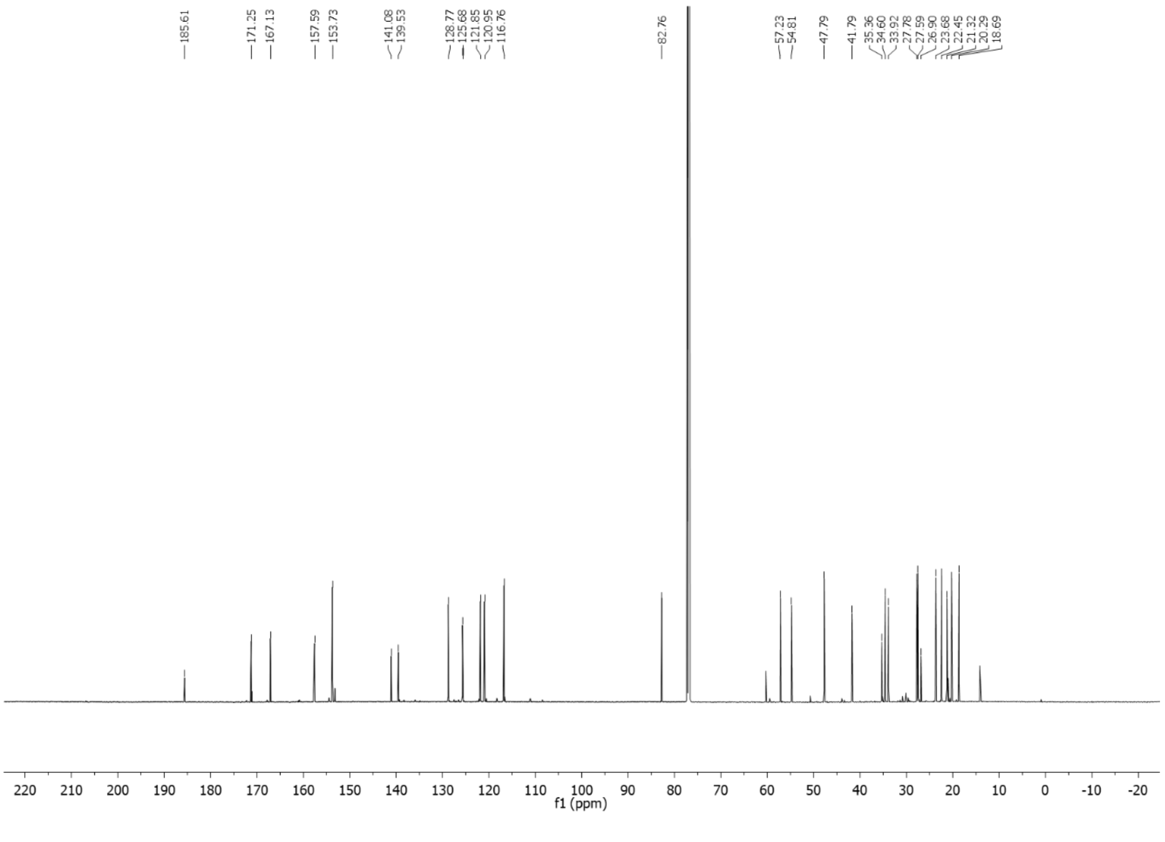
185.61
171.25
167.13
157.59
153.73
141.08
139.53
128.77
125.68
121.85
120.95
116.76
82.76
57.23
54.81
47.79
41.79
35.36
34.60
33.92
27.78
27.59
26.90
23.68
22.45
21.32
20.29
18.69
f1 (ppm)

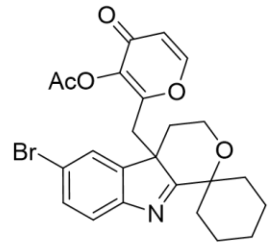

**3f**

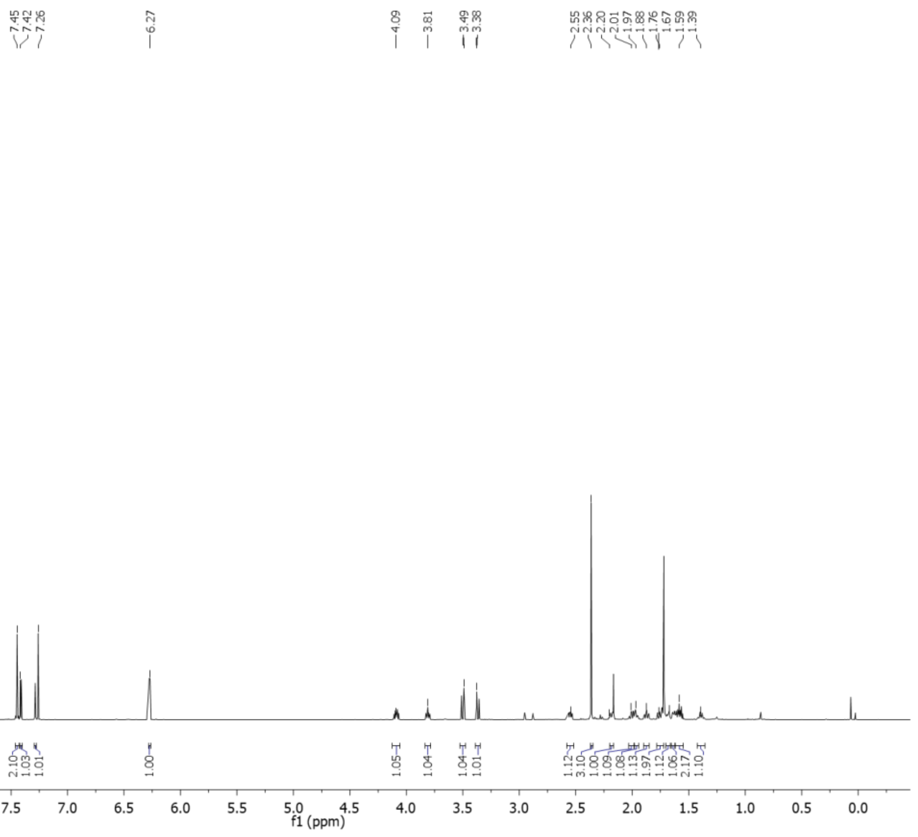

7.45
7.42
7.26
6.27
4.09
3.81
3.49
3.38
2.55
2.36
2.20
2.01
1.97
1.88
1.76
1.67
1.59
1.39
2.10
1.03
1.01
1.00
1.05
1.04
1.04
1.01
1.12
3.10
1.00
1.09
1.08
1.13
1.97
1.12
1.06
2.17
1.10
f1 (ppm)


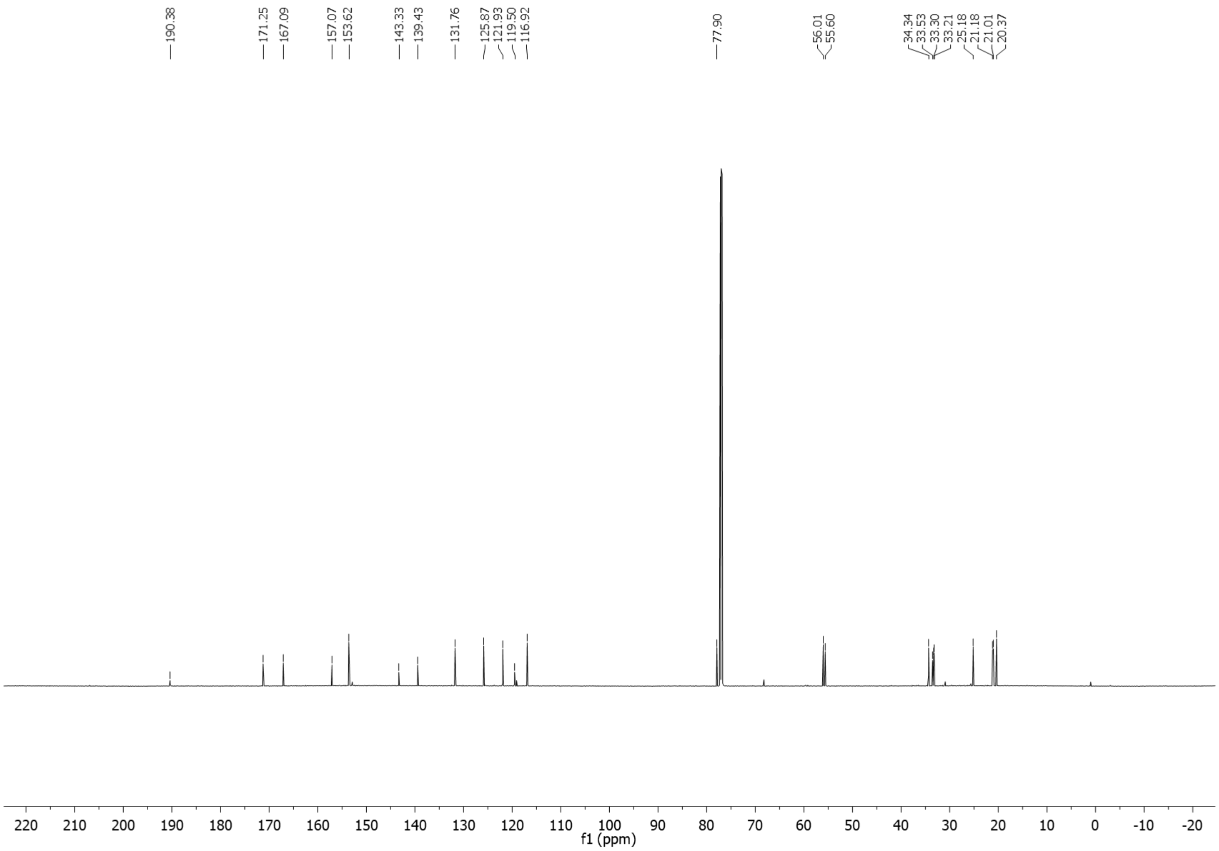

190.38
171.25
167.09
157.07
153.62
143.33
139.43
131.76
125.87
121.93
119.50
116.92
77.90
56.01
55.60
34.34
33.53
33.30
33.21
25.18
21.18
21.01
20.37
f1 (ppm)

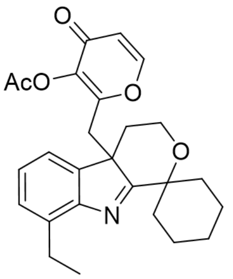
O
AcO
O
O
N

**3g**

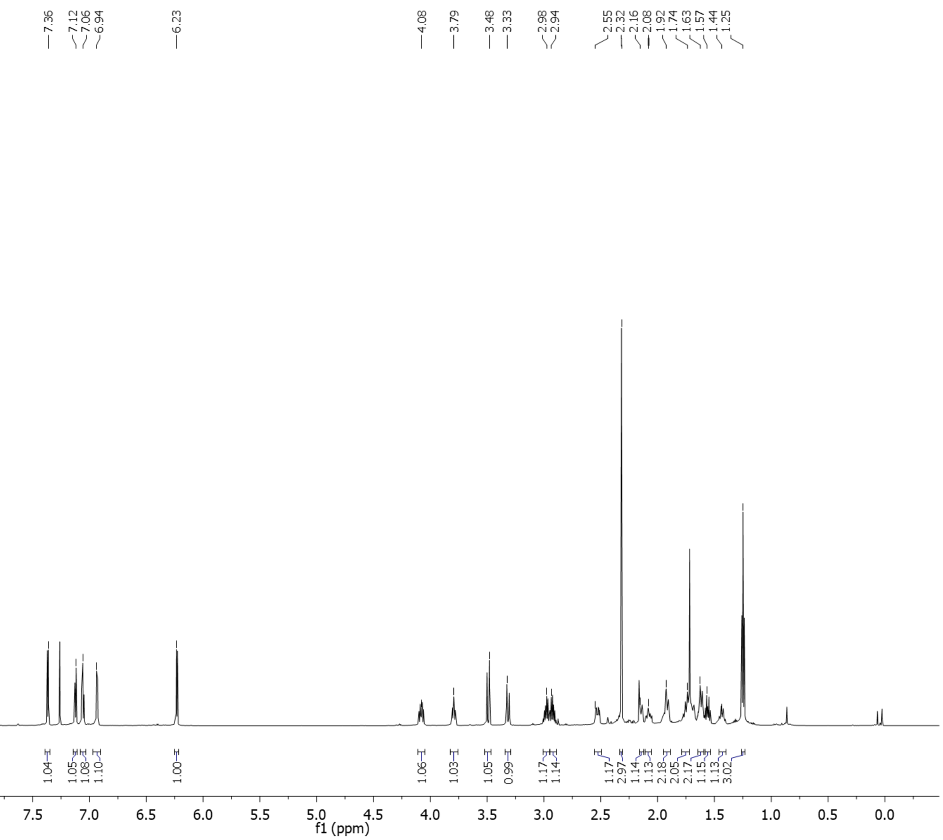
7.36
7.12
7.06
6.94
6.23
4.08
3.79
3.48
3.33
2.98
2.94
2.55
2.32
2.16
2.08
1.92
1.74
1.63
1.57
1.44
1.25
1.04
1.05
1.08
1.10
1.00
1.06
1.03
1.05
0.99
1.17
1.14
1.17
2.97
1.14
1.13
2.18
2.05
2.17
1.15
1.15
1.13
3.02
f1 (ppm)

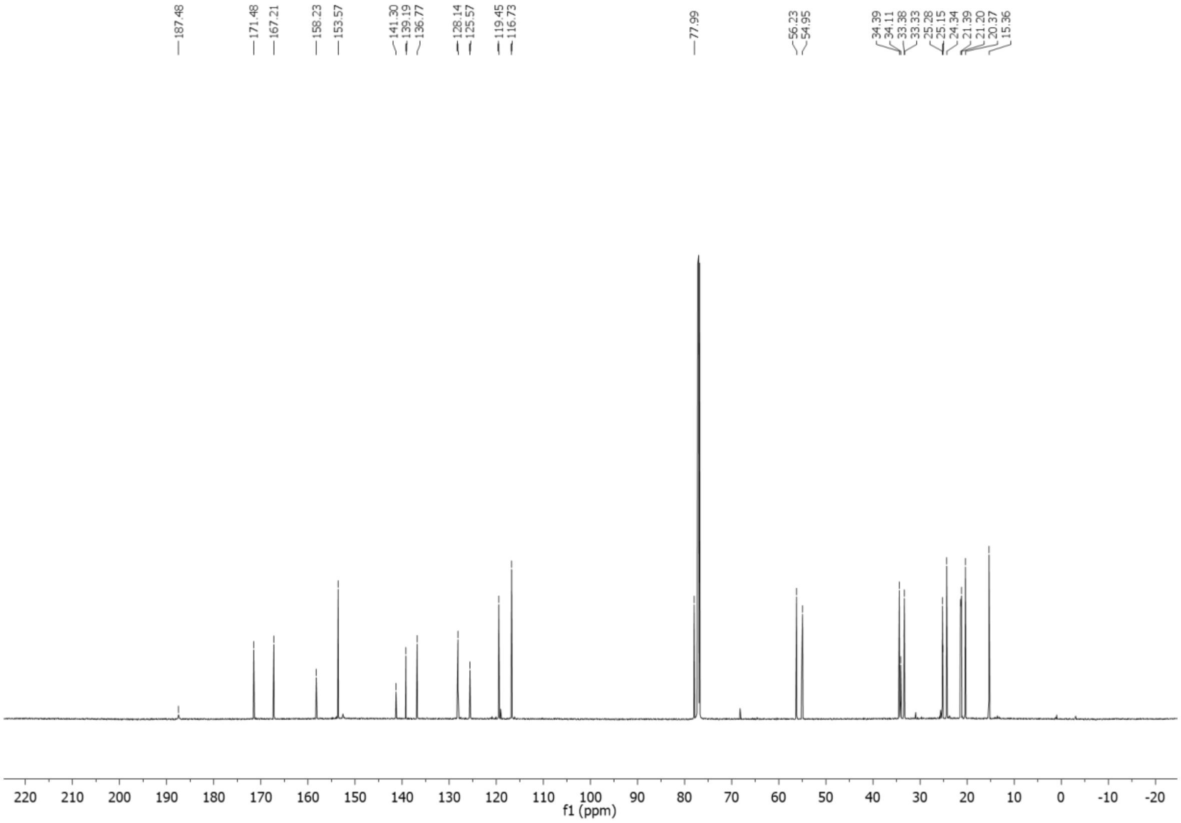
187.48
171.48
167.21
158.23
153.57
141.30
139.19
136.77
128.14
125.57
119.45
116.73
77.99
56.23
54.95
34.39
34.11
33.38
33.33
25.28
25.15
24.34
21.39
21.20
20.37
15.36
f1 (ppm)

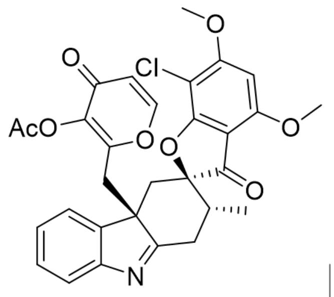
AcO
O
Cl
O
O
O
O
O
N

**7a**

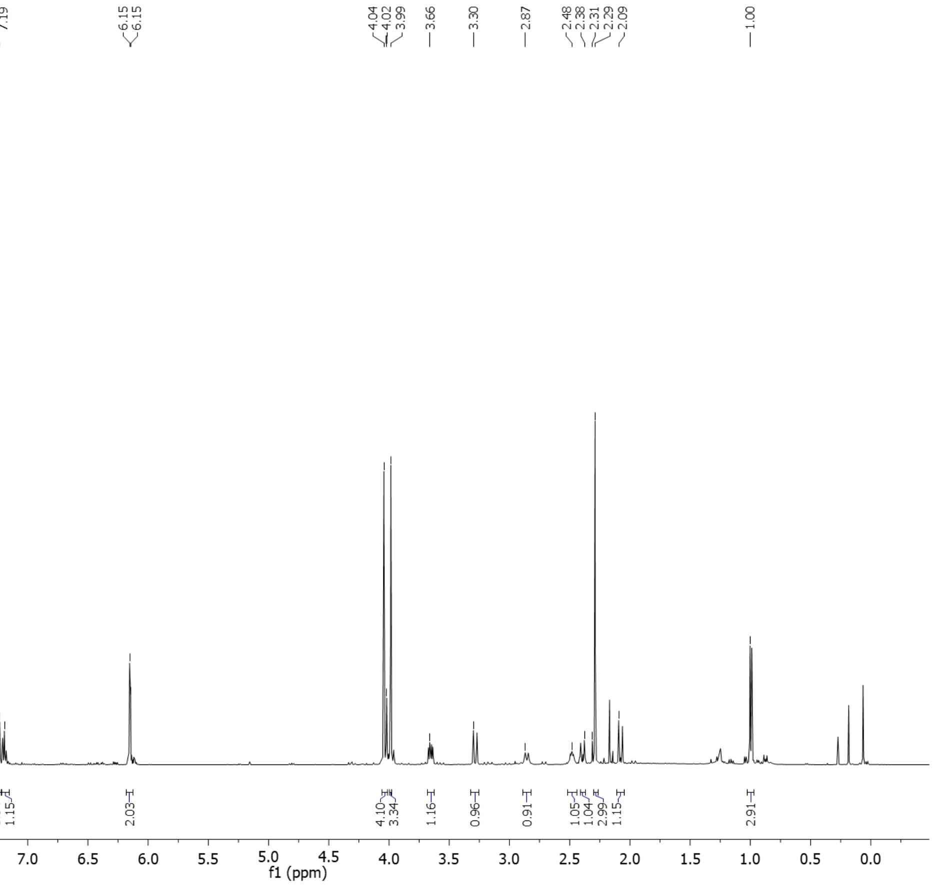
7.55
7.33
7.23
7.19
6.15
6.15
4.04
4.02
3.99
3.66
3.30
2.87
2.48
2.38
2.31
2.29
2.09
1.00
1.00
1.04
1.00
1.04
1.15
2.03
4.10
3.34
1.16
0.96
0.91
1.05
1.04
2.99
1.15
2.91
9.5 9.0 8.5 8.0 7.5 7.0 6.5 6.0 5.5 5.0 4.5 4.0 3.5 3.0 2.5 2.0 1.5 1.0 0.5 0.0
f1 (ppm)

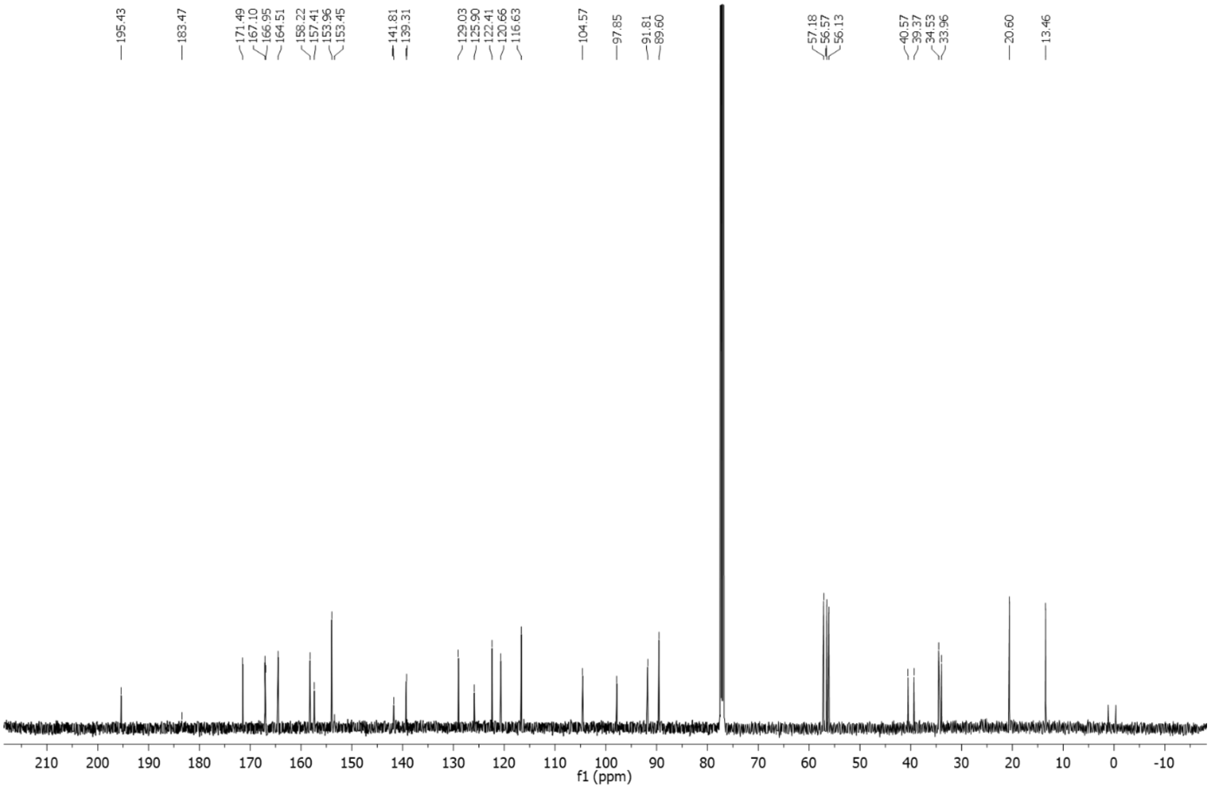
195.43
183.47
171.49
167.10
166.95
164.51
158.22
157.41
153.96
153.45
141.81
139.31
129.03
125.90
122.41
120.66
116.63
104.57
97.85
91.81
89.60
57.18
56.57
56.13
40.57
39.37
34.53
33.96
20.60
13.46
210 200 190 180 170 160 150 140 130 120 110 100 90 80 70 60 50 40 30 20 10 0 -10
f1 (ppm)

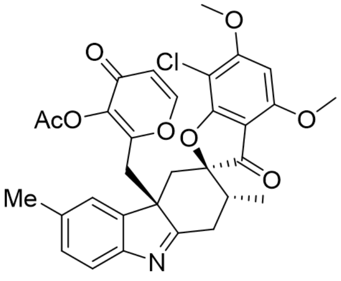
O
Cl
AcO
O
O
O
O
Me
N

**7b**

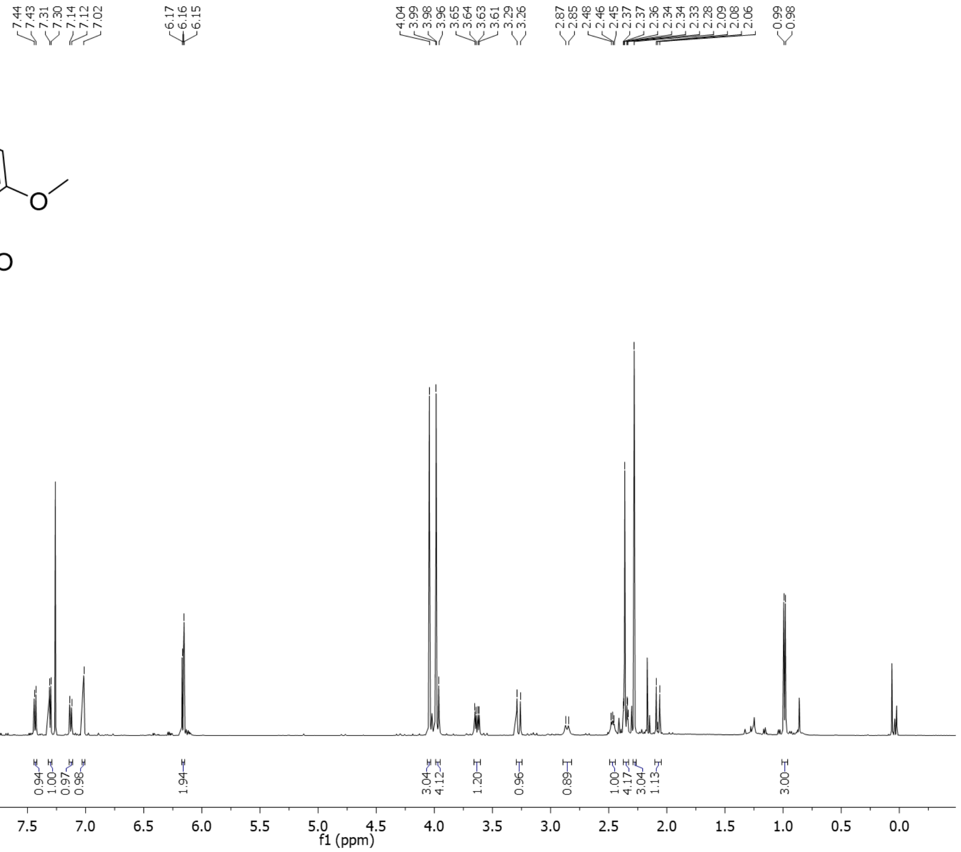
7.44
7.43
7.31
7.30
7.14
7.12
7.02
6.17
6.16
6.15
4.04
3.99
3.98
3.96
3.65
3.64
3.63
3.61
3.29
3.26
2.87
2.85
2.48
2.46
2.45
2.37
2.37
2.36
2.34
2.34
2.33
2.28
2.09
2.08
2.06
0.99
0.98
0.94
1.00
0.97
0.98
1.94
3.04
4.12
1.20
0.96
0.89
1.00
4.17
3.04
1.13
3.00
9.5 9.0 8.5 8.0 7.5 7.0 6.5 6.0 5.5 5.0 4.5 4.0 3.5 3.0 2.5 2.0 1.5 1.0 0.5 0.0
f1 (ppm)

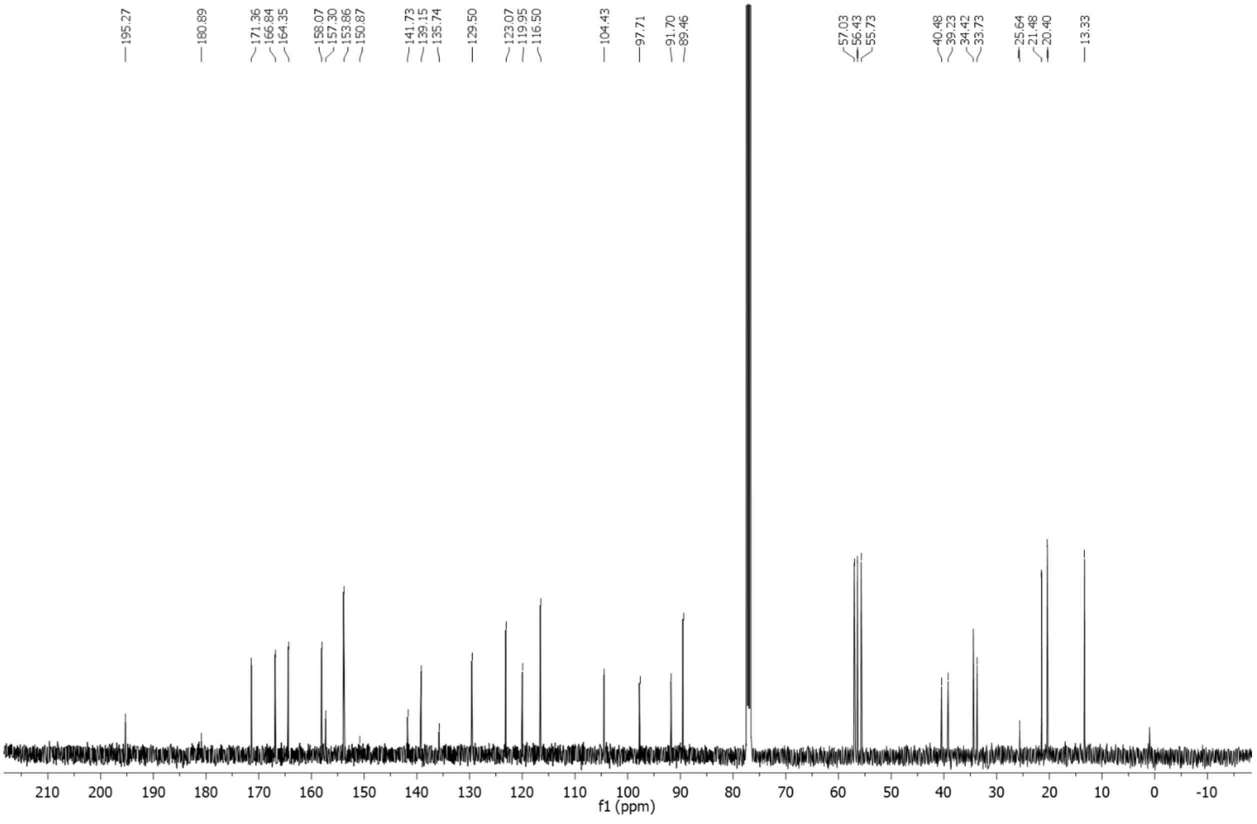
195.27
180.89
171.36
166.84
164.35
158.07
157.30
153.86
150.87
141.73
139.15
135.74
129.50
123.07
119.95
116.50
104.43
97.71
91.70
89.46
57.03
56.43
55.73
40.48
39.23
34.42
33.73
25.64
21.48
20.40
13.33
210 200 190 180 170 160 150 140 130 120 110 100 90 80 70 60 50 40 30 20 10 0 -10
f1 (ppm)

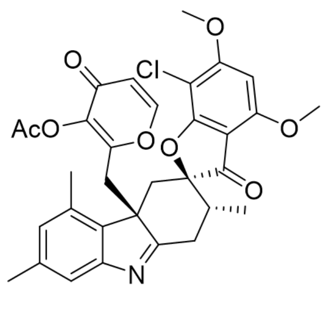
O
Cl
AcO
O
O
O
O
O
N

**7c**

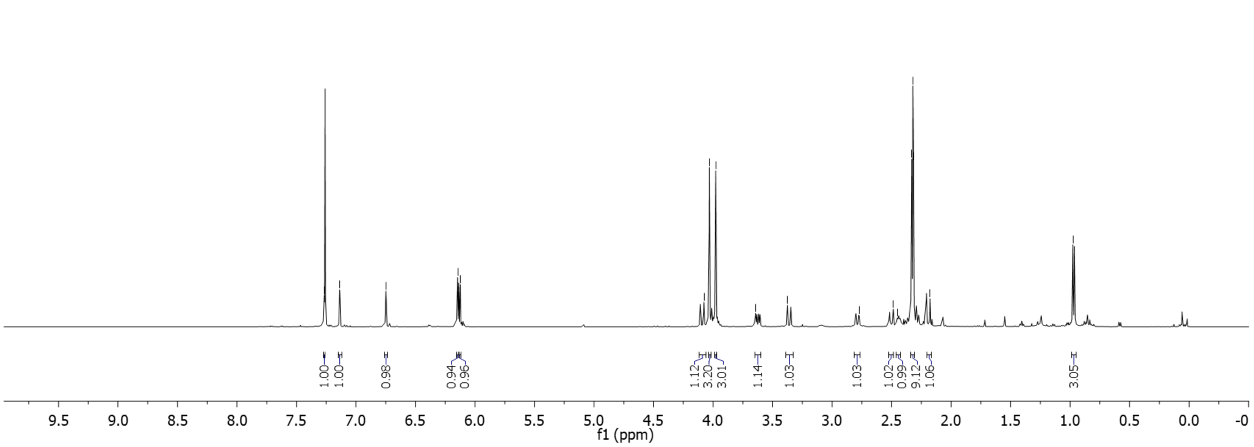
7.27
7.14
6.75
6.15
6.12
4.08
4.03
3.98
3.64
3.38
2.77
2.49
2.45
2.33
2.32
2.32
2.18
0.98
1.00
1.00
0.98
0.94
0.96
1.12
3.20
3.01
1.14
1.03
1.03
1.02
0.99
9.12
1.06
3.05
f1 (ppm)

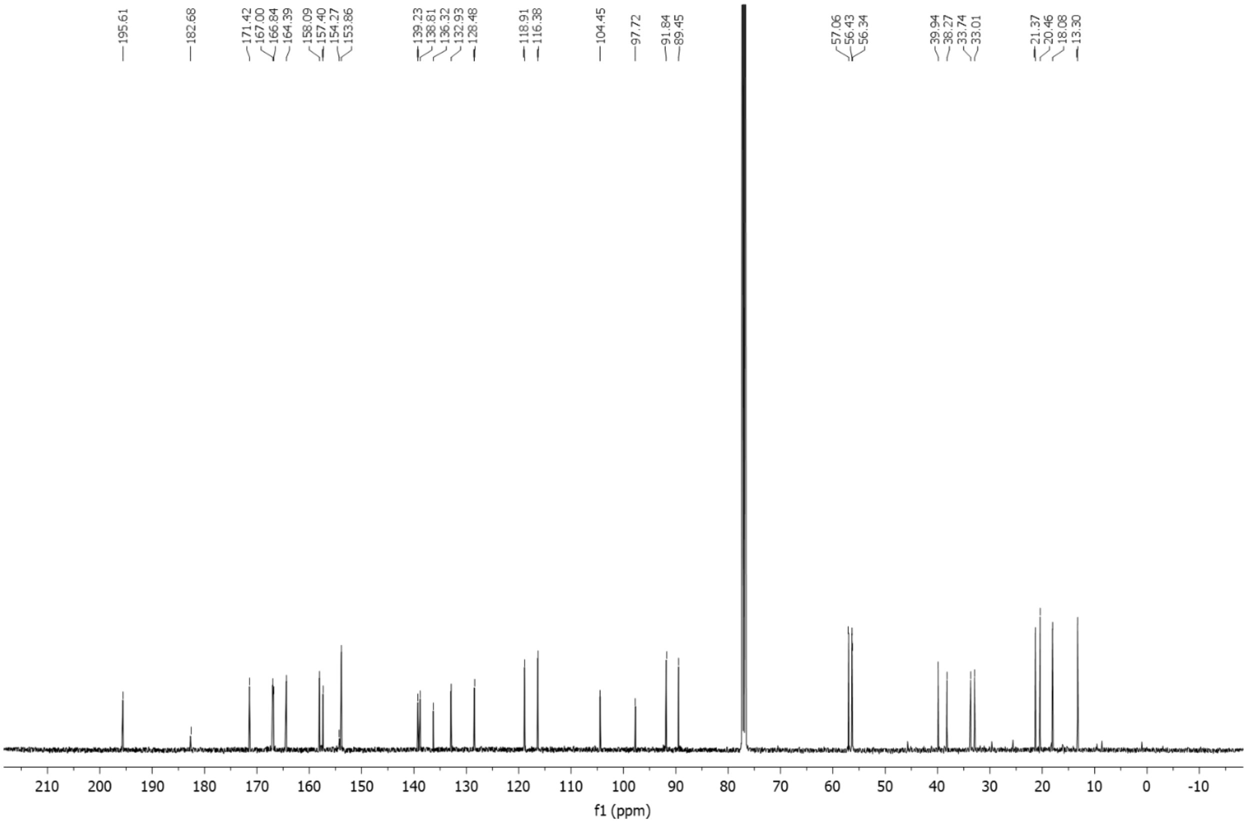
195.61
182.68
171.42
167.00
166.84
164.39
158.09
157.40
154.27
153.86
139.23
138.81
136.32
132.93
128.48
118.91
116.38
104.45
97.72
91.84
89.45
57.06
56.43
56.34
39.94
38.27
33.74
33.01
21.37
20.46
18.08
13.30
f1 (ppm)

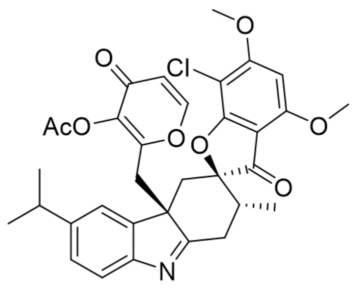
O
Cl
AcO
O
O
O
O
O
N

**7d**

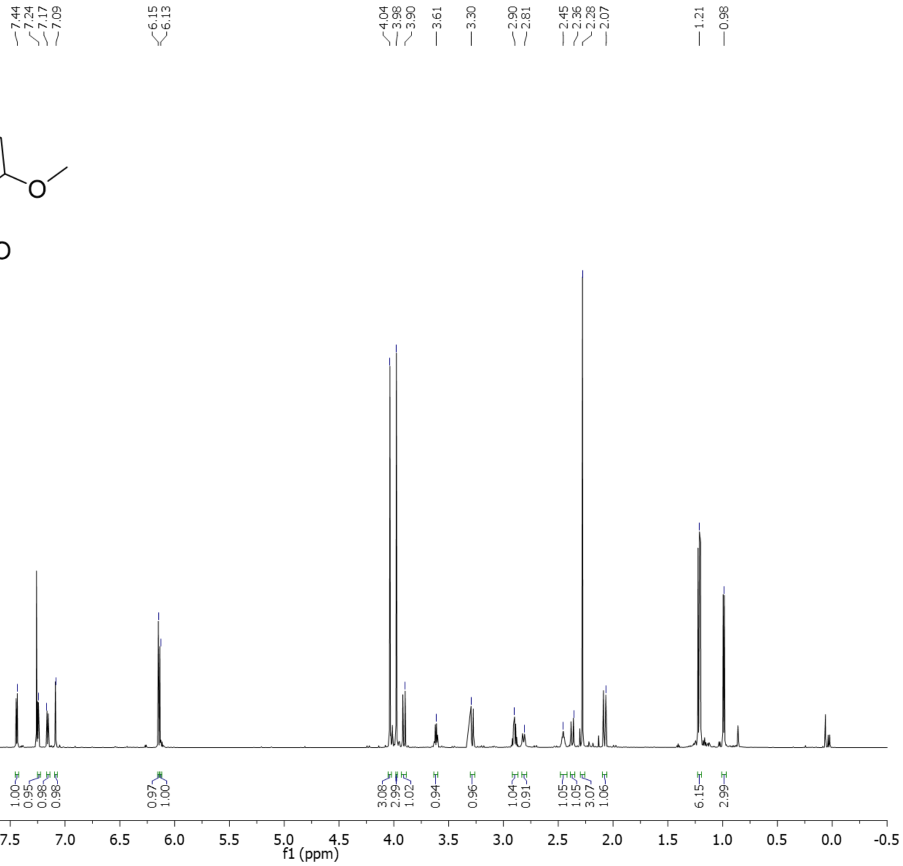
7.44
7.24
7.17
7.09
6.15
6.13
4.04
3.98
3.90
3.61
3.30
2.90
2.81
2.45
2.36
2.28
2.07
1.21
0.98
1.00
0.95
0.98
0.98
0.97
1.00
3.08
2.99
1.02
0.94
0.96
1.04
0.91
1.05
1.05
3.07
1.06
6.15
2.99
.0 9.5 9.0 8.5 8.0 7.5 7.0 6.5 6.0 5.5 5.0 4.5 4.0 3.5 3.0 2.5 2.0 1.5 1.0 0.5 0.0 -0.5
f1 (ppm)

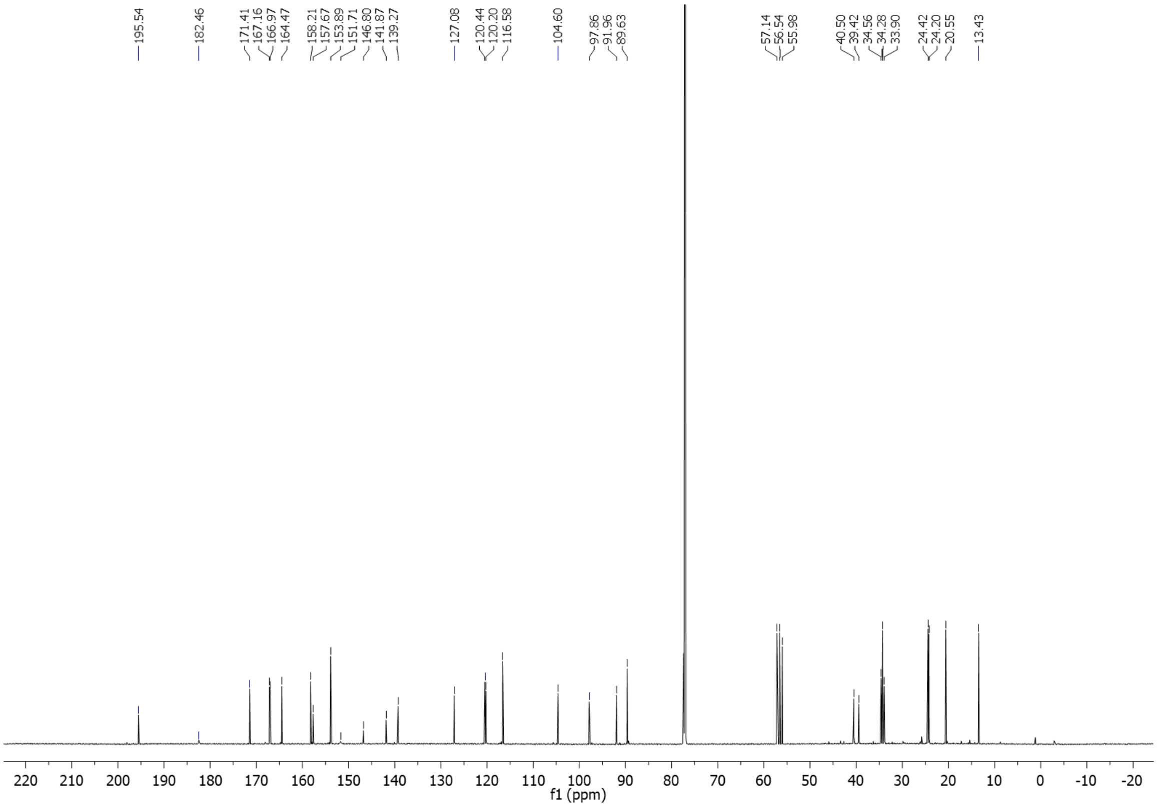
195.54
182.46
171.41
167.16
166.97
164.47
158.21
157.67
153.89
151.71
146.80
141.87
139.27
127.08
120.44
120.20
116.58
104.60
97.86
91.96
89.63
57.14
56.54
55.98
40.50
39.42
34.56
34.28
33.90
24.42
24.20
20.55
13.43
220 210 200 190 180 170 160 150 140 130 120 110 100 90 80 70 60 50 40 30 20 10 0 -10 -20
f1 (ppm)

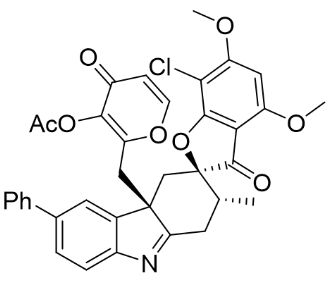

AcO
Cl
Ph
N


**7e**

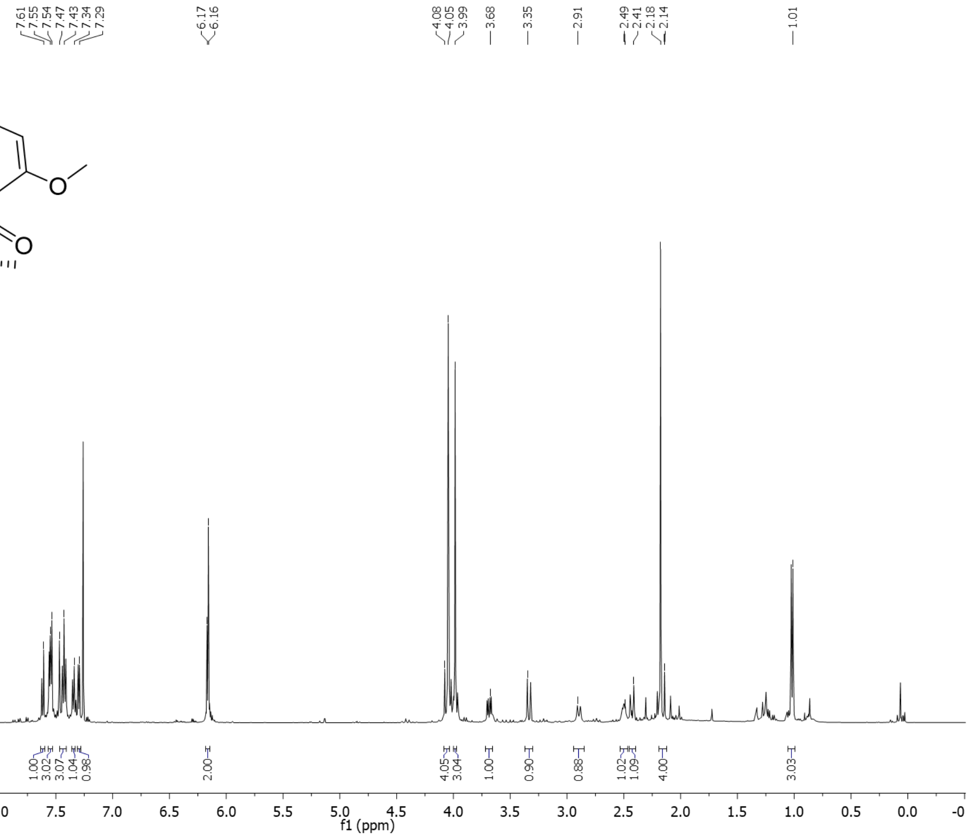

7.61
7.55
7.54
7.47
7.43
7.34
7.29
6.17
6.16
4.08
4.05
3.99
3.68
3.35
2.91
2.49
2.41
2.18
2.14
1.01
1.00
3.02
3.07
1.04
0.98
2.00
4.05
3.04
1.00
0.90
0.88
1.02
1.09
4.00
3.03
f1 (ppm)


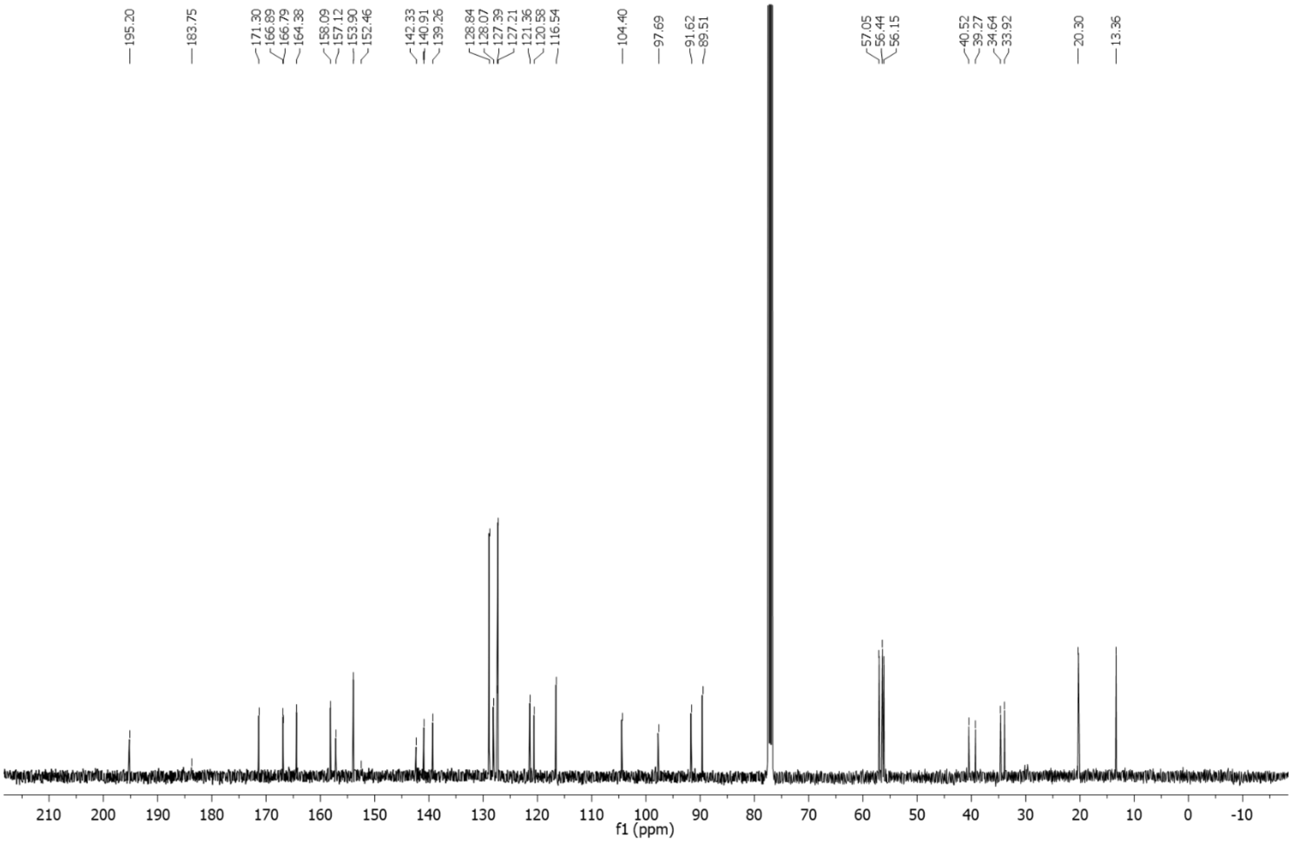

195.20
183.75
171.30
166.89
166.79
164.38
158.09
157.12
153.90
152.46
142.33
140.91
139.26
128.84
128.07
127.39
127.21
121.36
120.58
116.54
104.40
97.69
91.62
89.51
57.05
56.44
56.15
40.52
39.27
34.64
33.92
20.30
13.36
f1 (ppm)

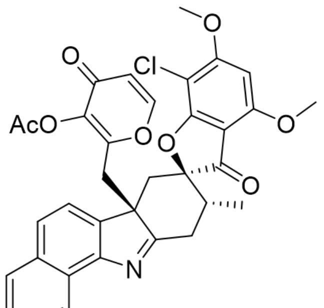
O
Cl
AcO
O
O
O
O
O
N

**7f**

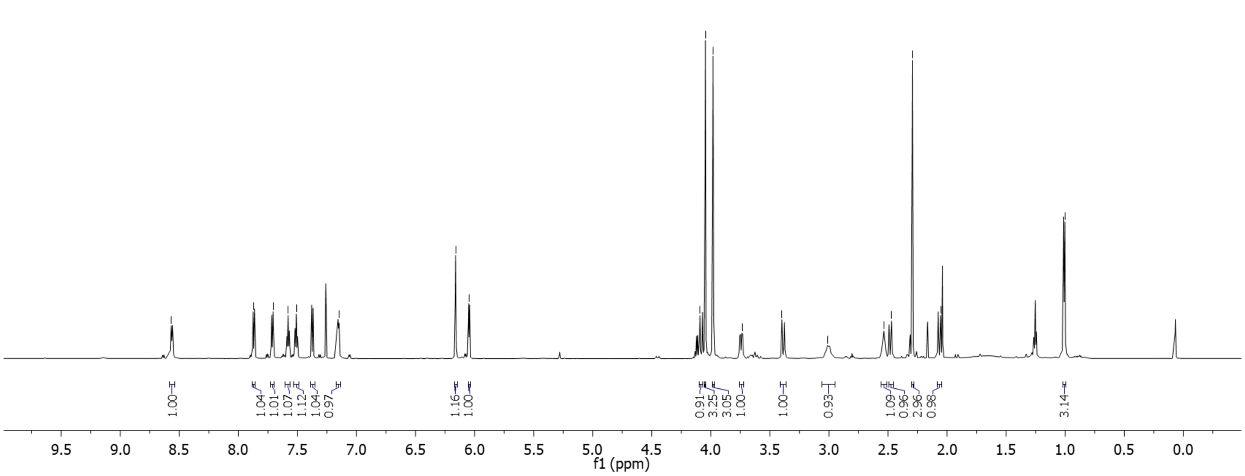
8.57
7.88
7.71
7.58
7.51
7.15
6.16
6.05
4.09
4.05
3.98
3.74
3.40
3.01
2.53
2.47
2.29
2.05
1.00
1.00
1.04
1.01
1.07
1.12
1.04
0.97
1.16
1.00
0.91
3.25
3.05
1.00
1.00
0.93
1.09
0.96
2.96
0.98
3.14
f1 (ppm)

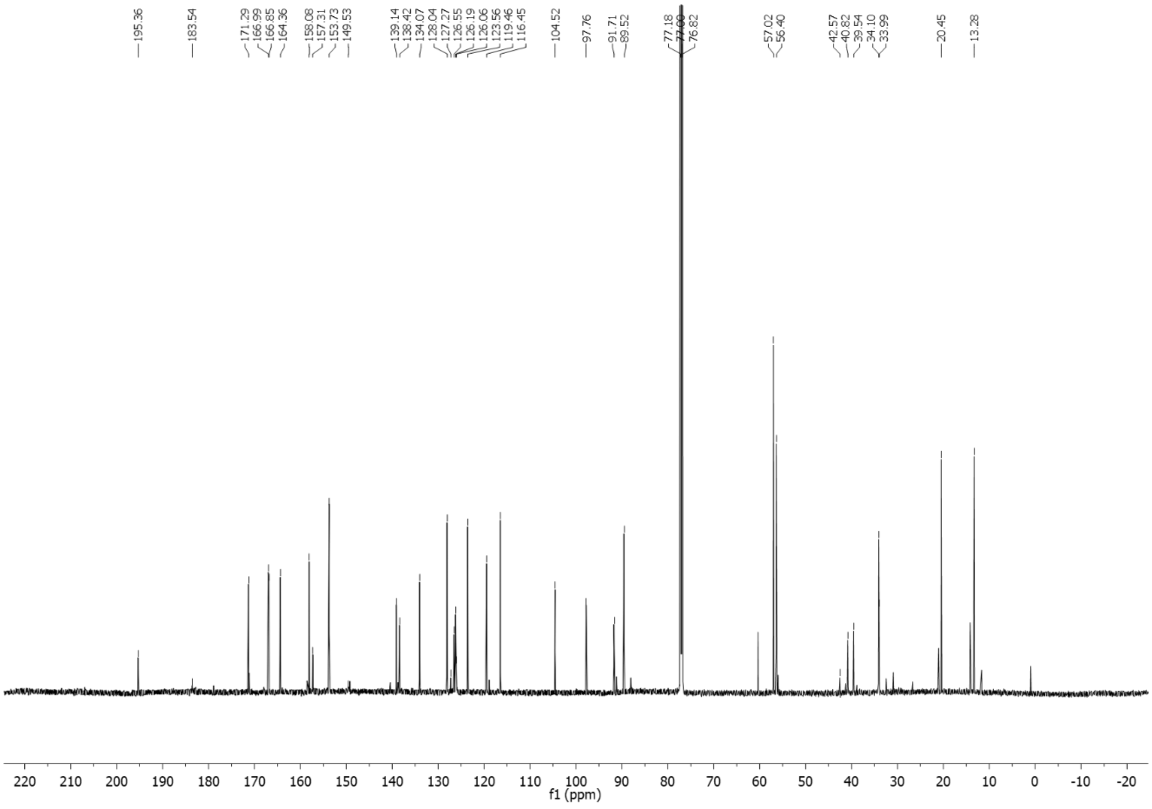
195.36
183.54
171.29
166.99
166.85
164.36
158.08
157.31
153.73
149.53
139.14
138.42
134.07
128.04
127.27
126.55
126.19
126.06
123.56
119.46
116.45
104.52
97.76
91.71
89.52
77.18
77.00
76.82
57.02
56.40
42.57
40.82
39.54
34.10
33.99
20.45
13.28
f1 (ppm)

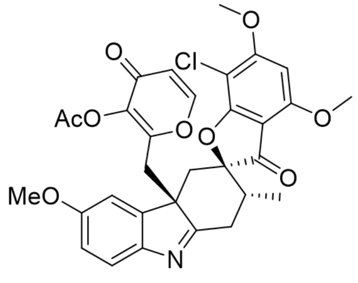
O
Cl
AcO
O
O
O
O
O
MeO
N

**7g**

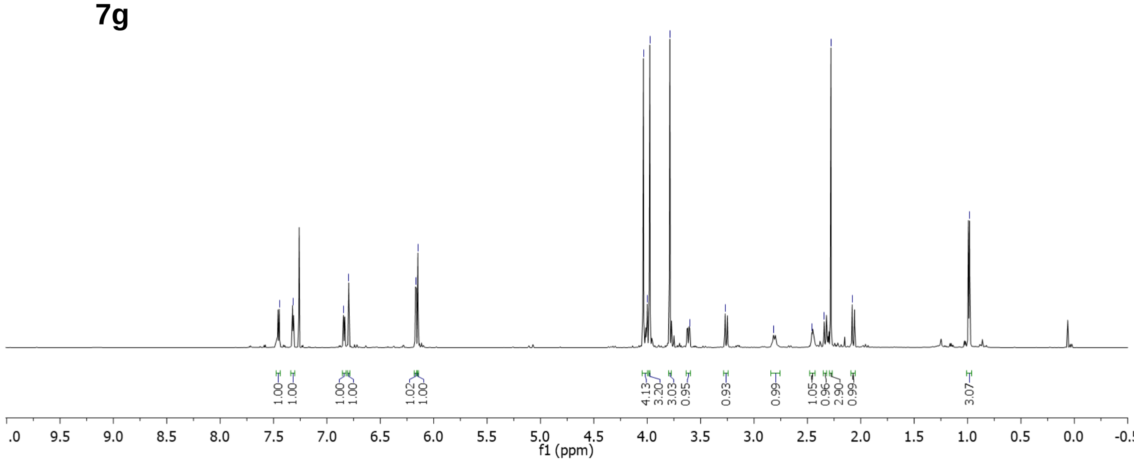
7.45
7.31
6.85
6.80
6.17
6.15
4.04
4.00
3.98
3.79
3.60
3.27
2.82
2.46
2.34
2.28
2.08
0.98
1.00
1.00
1.00
1.00
1.02
1.00
4.13
3.20
3.03
0.95
0.93
0.99
1.05
0.96
2.90
0.99
3.07
f1 (ppm)

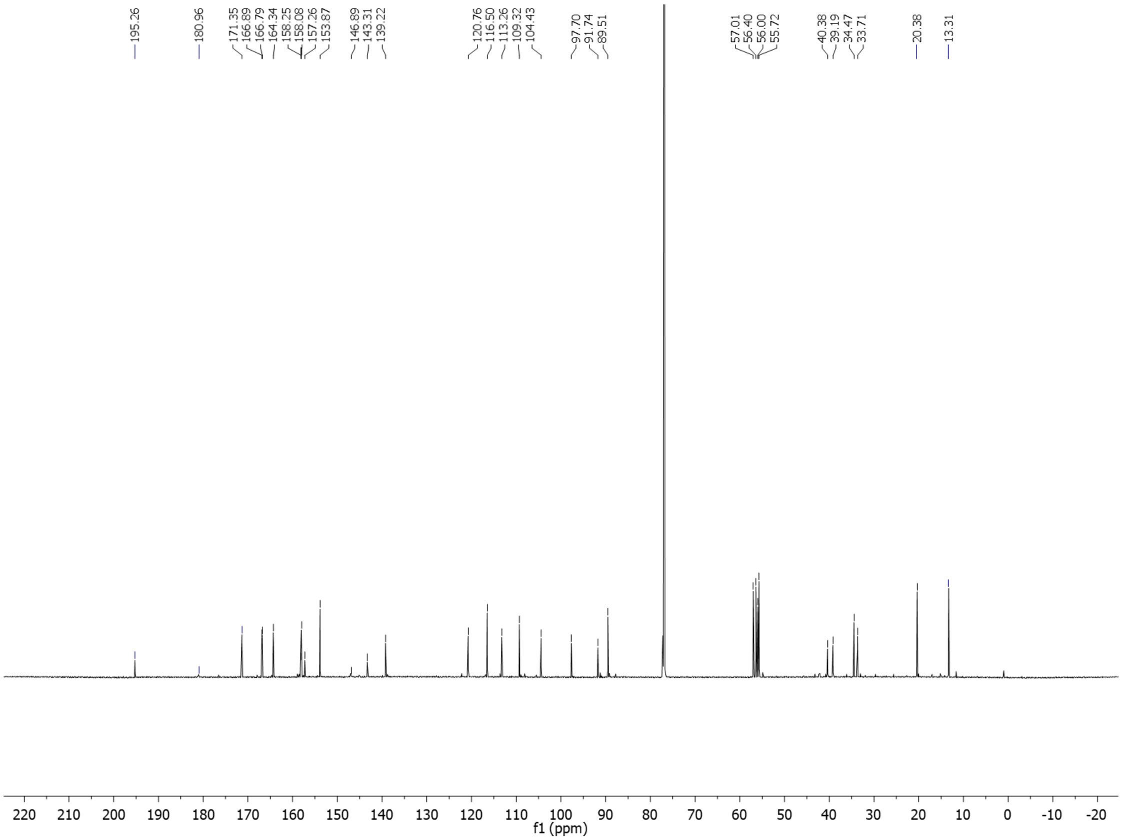
195.26
180.96
171.35
166.89
166.79
164.34
158.25
158.08
157.26
153.87
146.89
143.31
139.22
120.76
116.50
113.26
109.32
104.43
97.70
91.74
89.51
57.01
56.40
56.00
55.72
40.38
39.19
34.47
33.71
20.38
13.31
f1 (ppm)

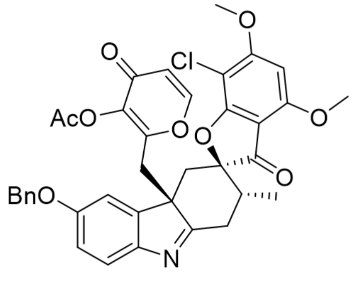
O
Cl
AcO
BnO
N
O
O
O
O

**7h**

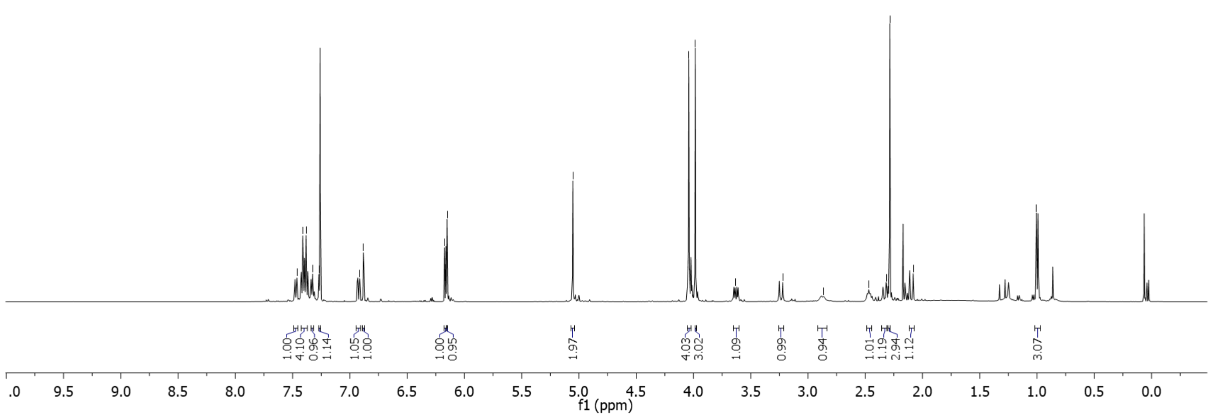
7.46
7.41
7.38
7.32
7.27
6.91
6.88
6.17
6.15
5.05
4.05
4.04
3.98
3.64
3.22
2.86
2.47
2.32
2.29
2.08
1.01
1.00
4.10
0.96
1.14
1.05
1.00
1.00
0.95
1.97
4.03
3.02
1.09
0.99
0.94
1.01
1.19
2.94
1.12
3.07
f1 (ppm)

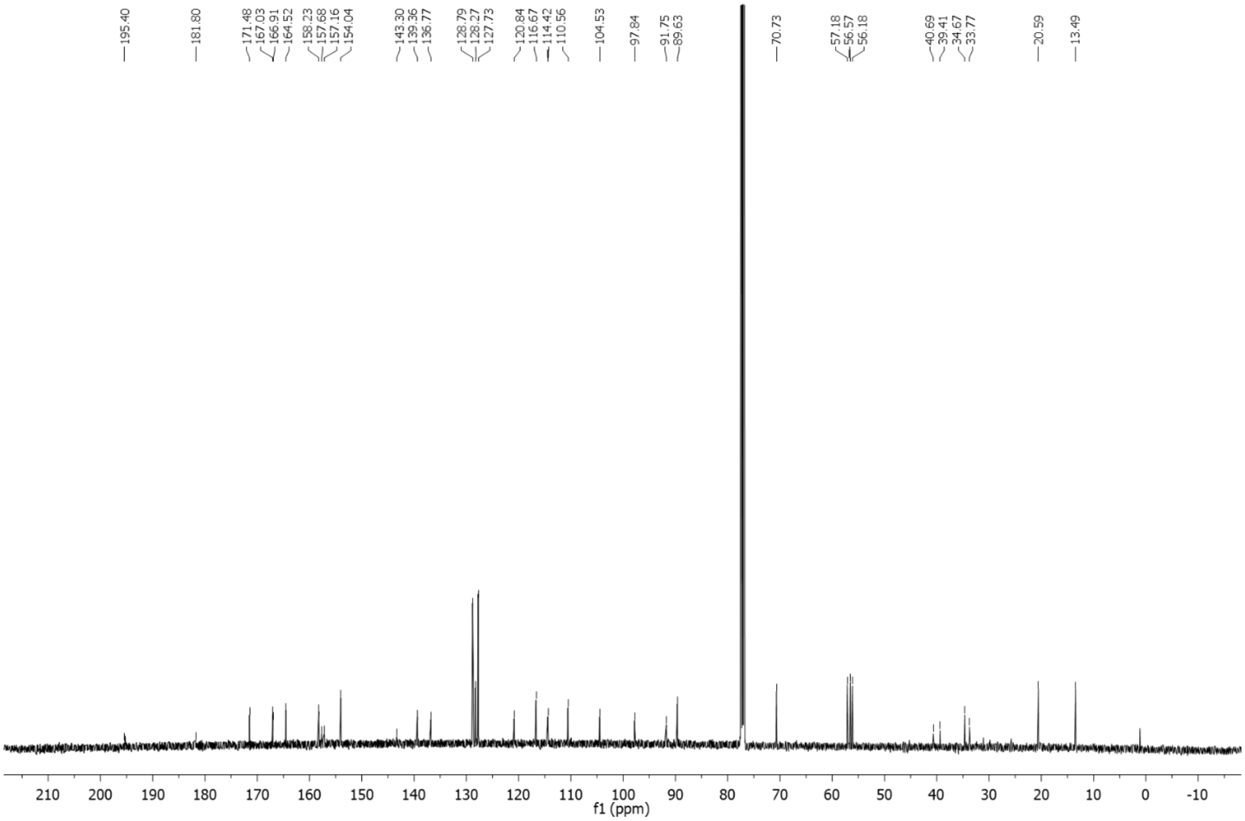
195.40
181.80
171.48
167.03
166.91
164.52
158.23
157.68
157.16
154.04
143.30
139.36
136.77
128.79
128.27
127.73
120.84
116.67
114.42
110.56
104.53
97.84
91.75
89.63
70.73
57.18
56.57
56.18
40.69
39.41
34.67
33.77
20.59
13.49
f1 (ppm)

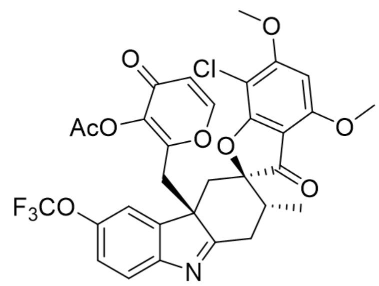
O
Cl
AcO
O
O
O
O
O
$F_3CO$
N

**7i**

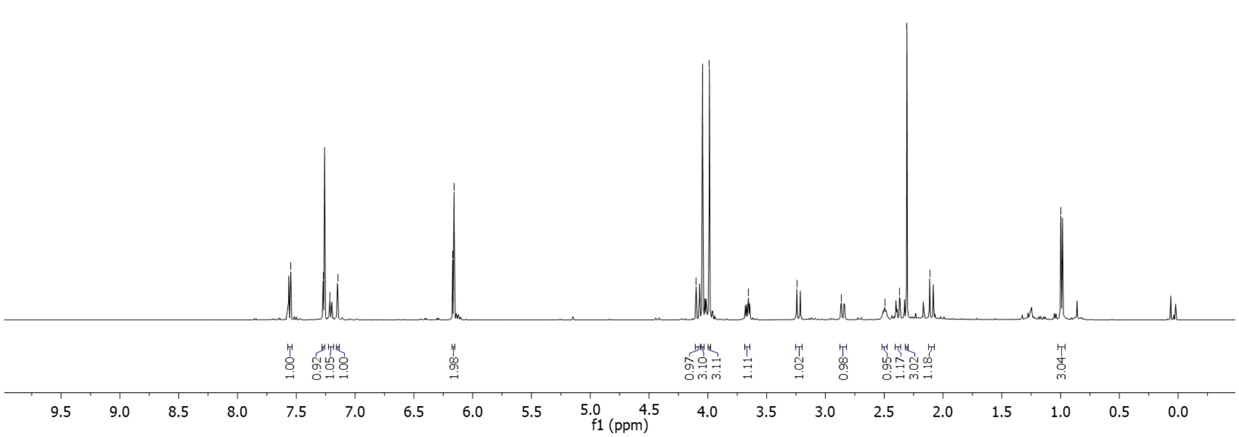
7.55
7.27
7.22
7.15
6.17
6.16
4.10
4.04
3.99
3.65
3.24
2.87
2.50
2.37
2.31
2.11
1.00
1.00
0.92
1.05
1.00
1.98
0.97
3.10
3.11
1.11
1.02
0.98
0.95
1.17
3.02
1.18
3.04
9.5 9.0 8.5 8.0 7.5 7.0 6.5 6.0 5.5 5.0 4.5 4.0 3.5 3.0 2.5 2.0 1.5 1.0 0.5 0.0
f1 (ppm)

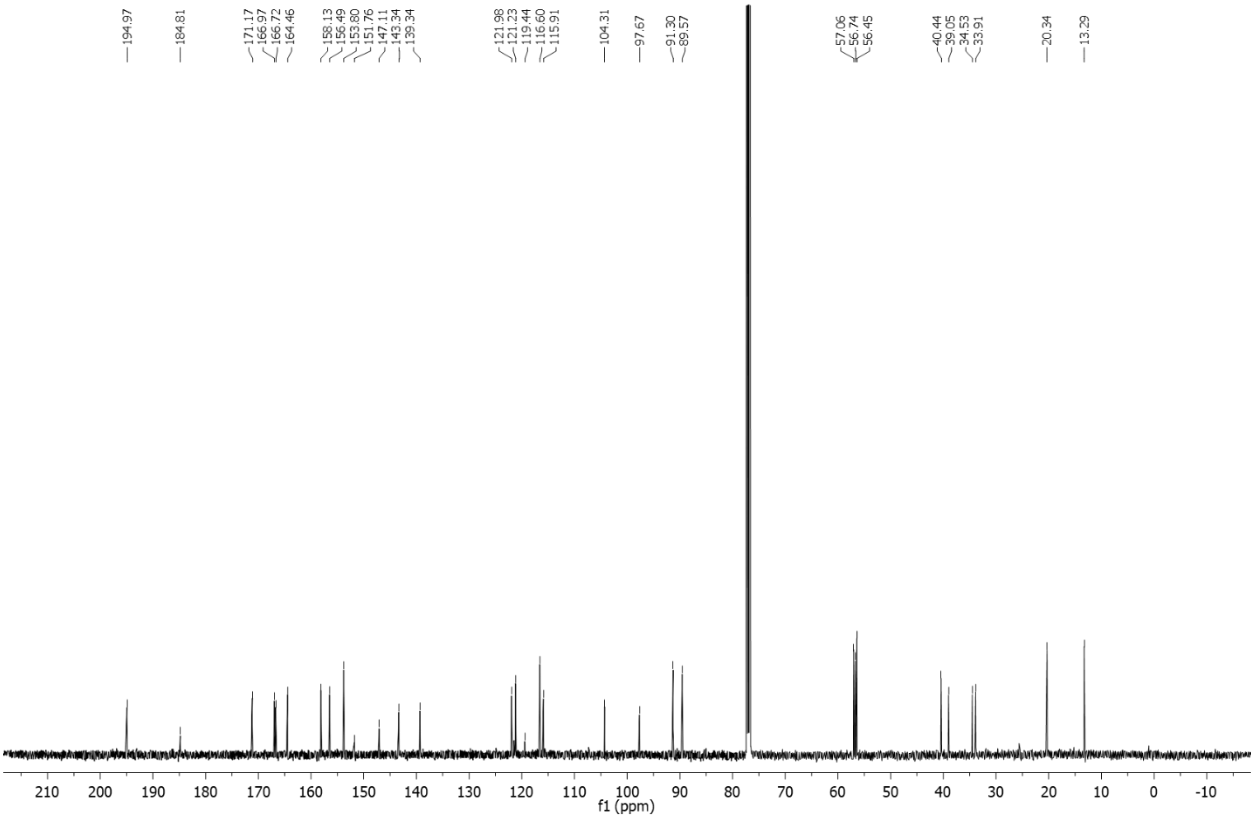
194.97
184.81
171.17
166.97
166.72
164.46
158.13
156.49
153.80
151.76
147.11
143.34
139.34
121.98
121.23
119.44
116.60
115.91
104.31
97.67
91.30
89.57
57.06
56.74
56.45
40.44
39.05
34.53
33.91
20.34
13.29
210 200 190 180 170 160 150 140 130 120 110 100 90 80 70 60 50 40 30 20 10 0 -10
f1 (ppm)

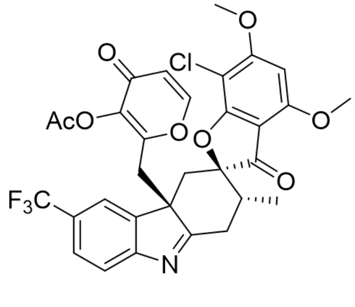

O
Cl
AcO
O
O
O
O
F3C
N


**7j**

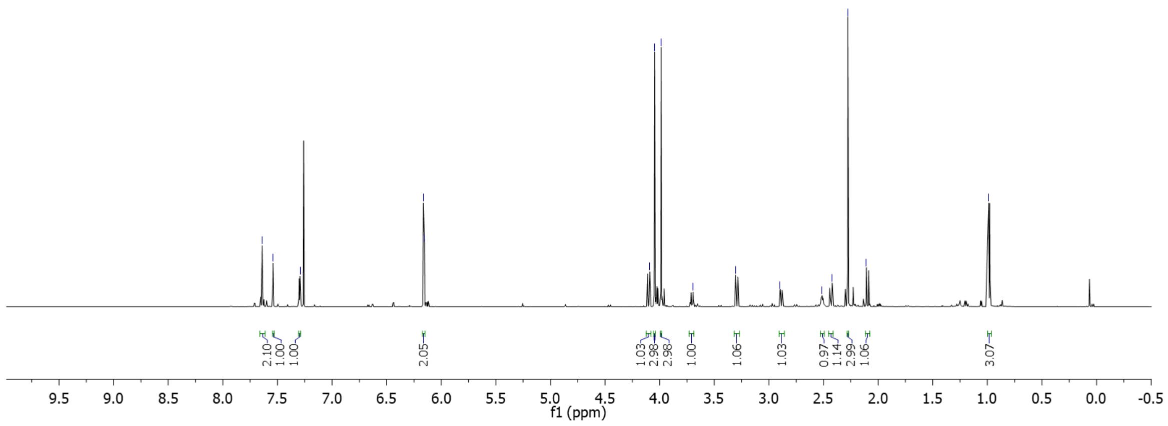

7.64
7.54
7.29
6.16
6.15
4.09
4.05
3.99
3.69
3.30
2.90
2.52
2.42
2.28
2.11
0.99
2.10
1.00
1.00
2.05
1.03
2.98
2.98
1.00
1.06
1.03
0.97
1.14
2.99
1.06
3.07
9.5 9.0 8.5 8.0 7.5 7.0 6.5 6.0 5.5 5.0 4.5 4.0 3.5 3.0 2.5 2.0 1.5 1.0 0.5 0.0 -0.5
f1 (ppm)


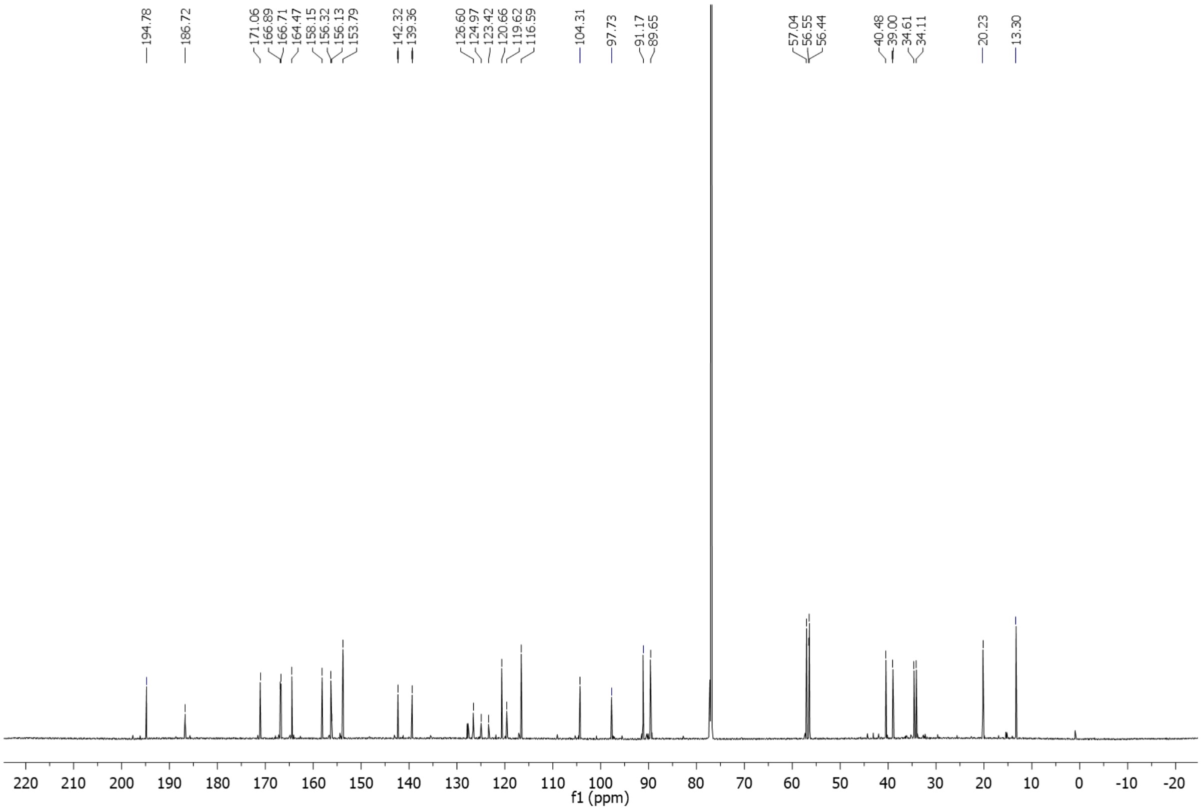

194.78
186.72
171.06
166.89
166.71
164.47
158.15
156.32
156.13
153.79
142.32
139.36
126.60
124.97
123.42
120.66
119.62
116.59
104.31
97.73
91.17
89.65
57.04
56.55
56.44
40.48
39.00
34.61
34.11
20.23
13.30
220 210 200 190 180 170 160 150 140 130 120 110 100 90 80 70 60 50 40 30 20 10 0 -10 -20
f1 (ppm)

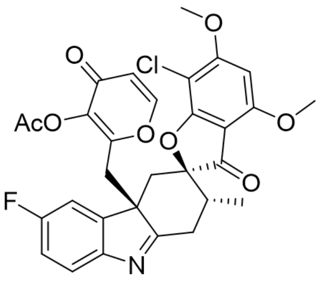
O
Cl
AcO
O
O
O
O
F
N

**7k**

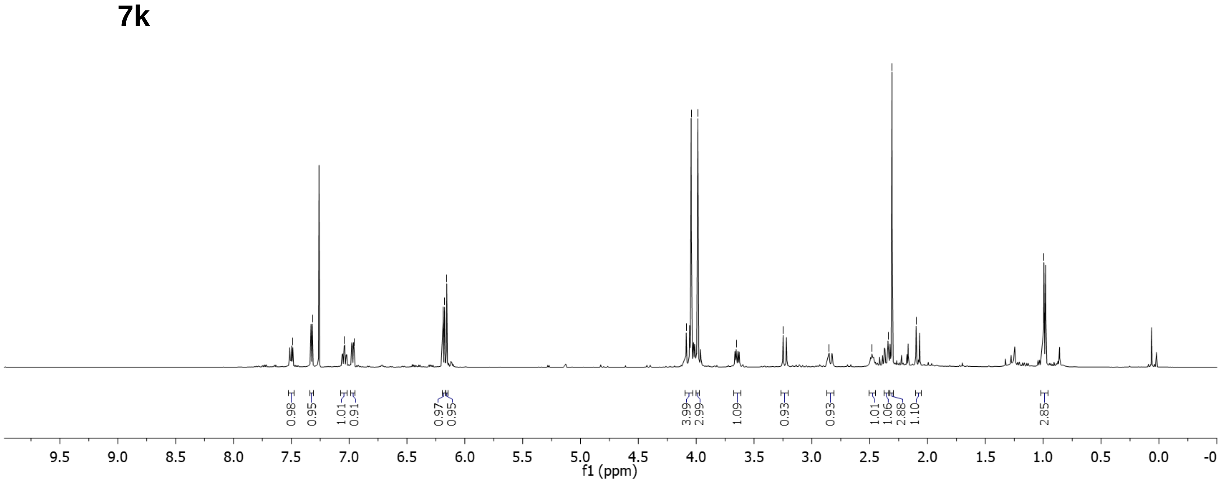
7.49
7.32
7.04
6.96
6.18
6.16
4.09
4.04
3.99
3.65
3.25
2.85
2.48
2.34
2.31
2.10
0.99
0.98
0.95
1.01
0.91
0.97
0.95
3.99
2.99
1.09
0.93
0.93
1.01
1.06
2.88
1.10
2.85
9.5 9.0 8.5 8.0 7.5 7.0 6.5 6.0 5.5 5.0 4.5 4.0 3.5 3.0 2.5 2.0 1.5 1.0 0.5 0.0 -0
f1 (ppm)

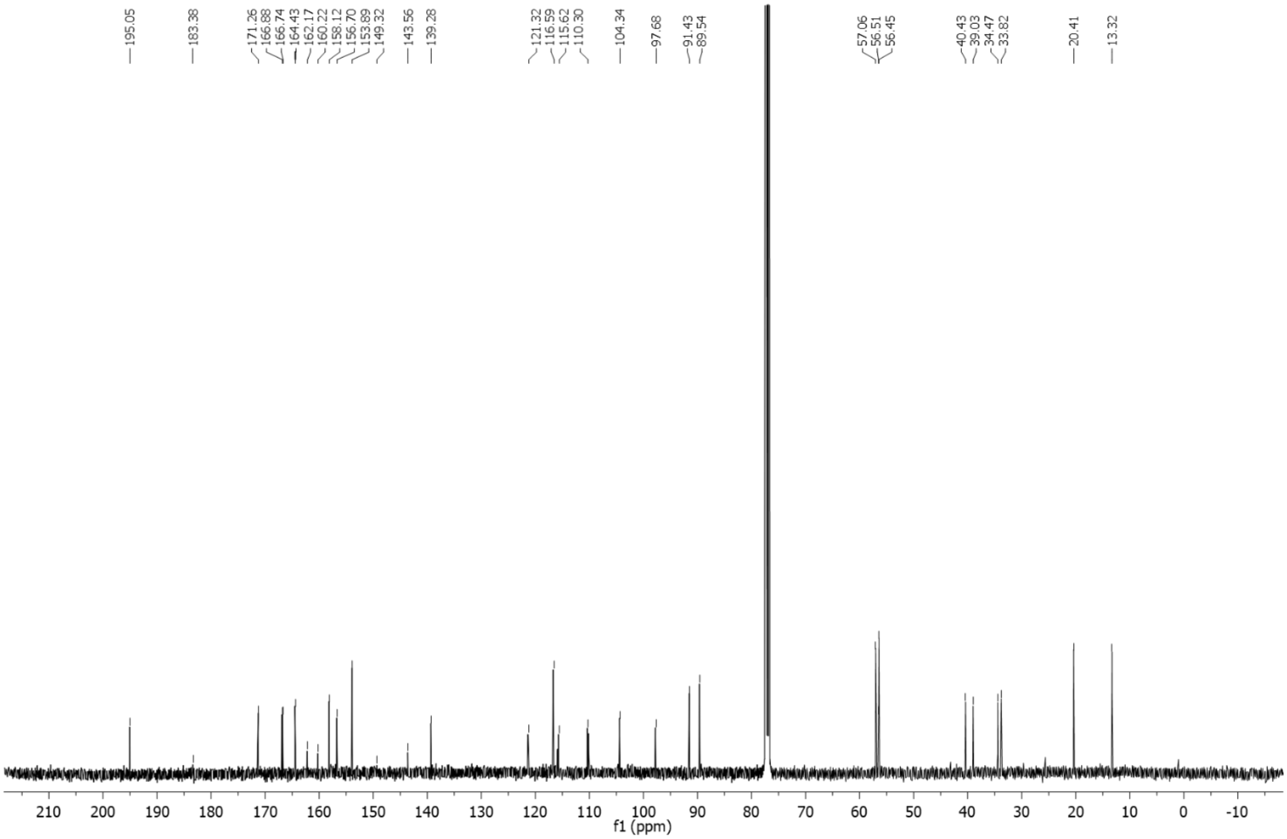
195.05
183.38
171.26
166.88
166.74
164.43
162.17
160.22
158.12
156.70
153.89
149.32
143.56
139.28
121.32
116.59
115.62
110.30
104.34
97.68
91.43
89.54
57.06
56.51
56.45
40.43
39.03
34.47
33.82
20.41
13.32
210 200 190 180 170 160 150 140 130 120 110 100 90 80 70 60 50 40 30 20 10 0 -10
f1 (ppm)

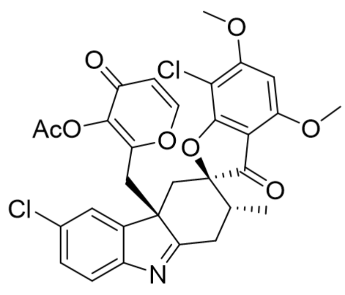
O
Cl
AcO
O
O
O
Cl
N
O
O
O

**7l**

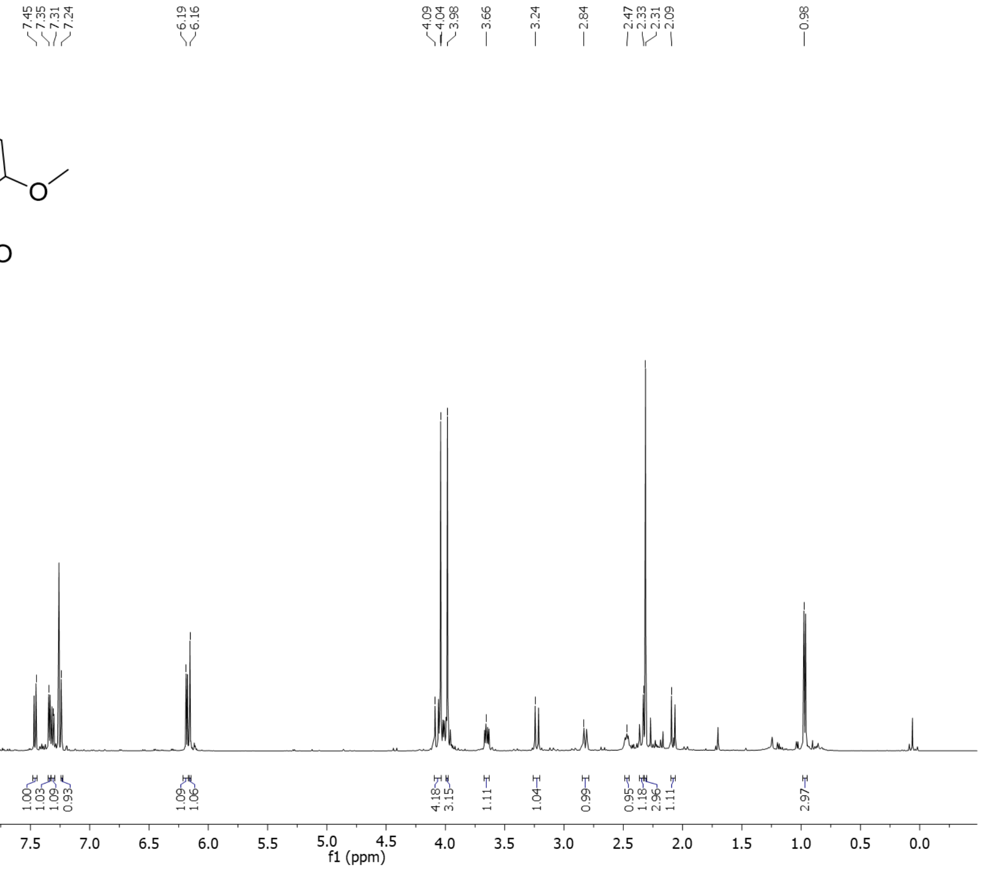
7.45
7.35
7.31
7.24
6.19
6.16
4.09
4.04
3.98
3.66
3.24
2.84
2.47
2.33
2.31
2.09
0.98
1.00
1.03
1.09
0.93
1.09
1.06
4.18
3.15
1.11
1.04
0.99
0.95
1.18
2.96
1.11
2.97
9.5
9.0
8.5
8.0
7.5
7.0
6.5
6.0
5.5
5.0
4.5
4.0
3.5
3.0
2.5
2.0
1.5
1.0
0.5
0.0
f1 (ppm)

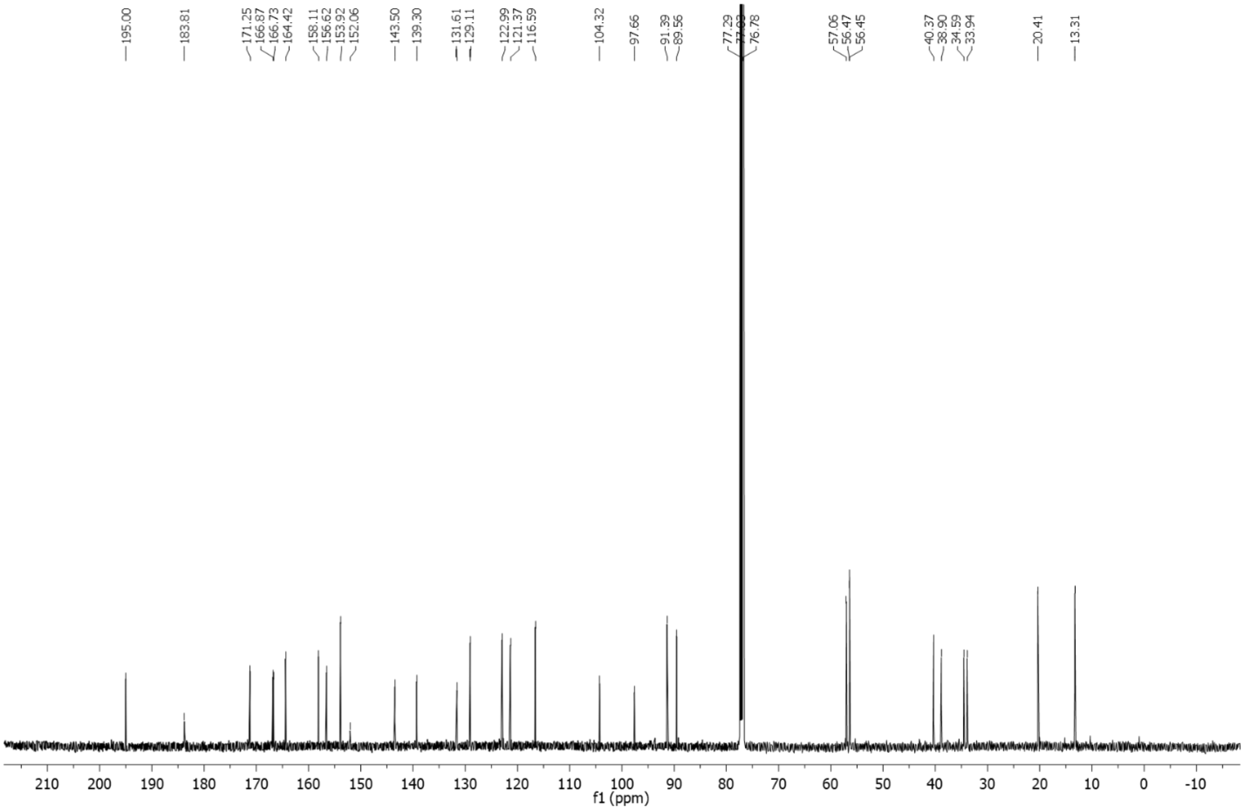
195.00
183.81
171.25
166.87
166.73
164.42
158.11
156.62
153.92
152.06
143.50
139.30
131.61
129.11
122.99
121.37
116.59
104.32
97.66
91.39
89.56
77.29
76.78
57.06
56.47
56.45
40.37
38.90
34.59
33.94
20.41
13.31
210
200
190
180
170
160
150
140
130
120
110
100
90
80
70
60
50
40
30
20
10
0
-10
f1 (ppm)

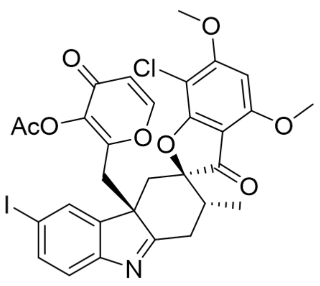

O
Cl
AcO
O
O
O
O
I
N


**7m**

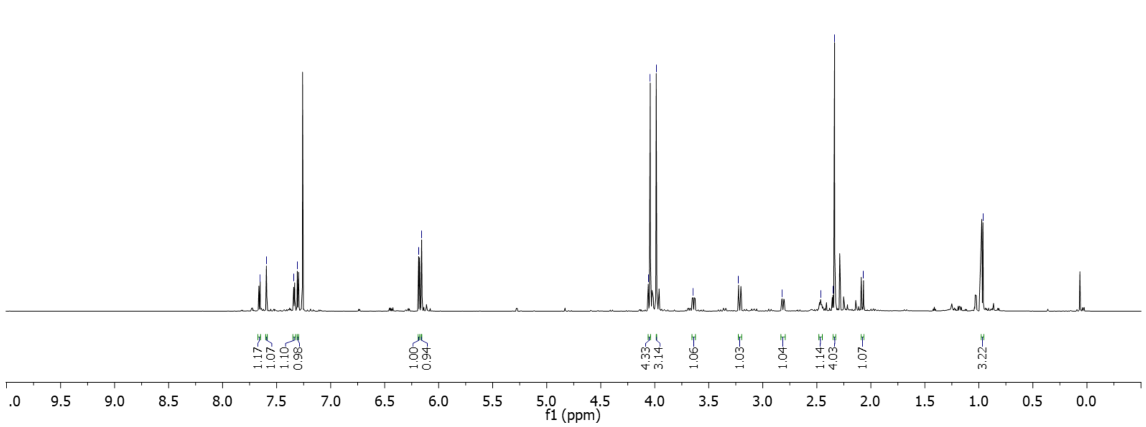

7.66
7.59
7.34
7.31
6.19
6.16
4.06
4.04
3.99
3.65
3.22
2.82
2.46
2.35
2.34
2.07
0.96
1.17
1.07
1.10
0.98
1.00
0.94
4.33
3.14
1.06
1.03
1.04
1.14
4.03
1.07
3.22
f1 (ppm)


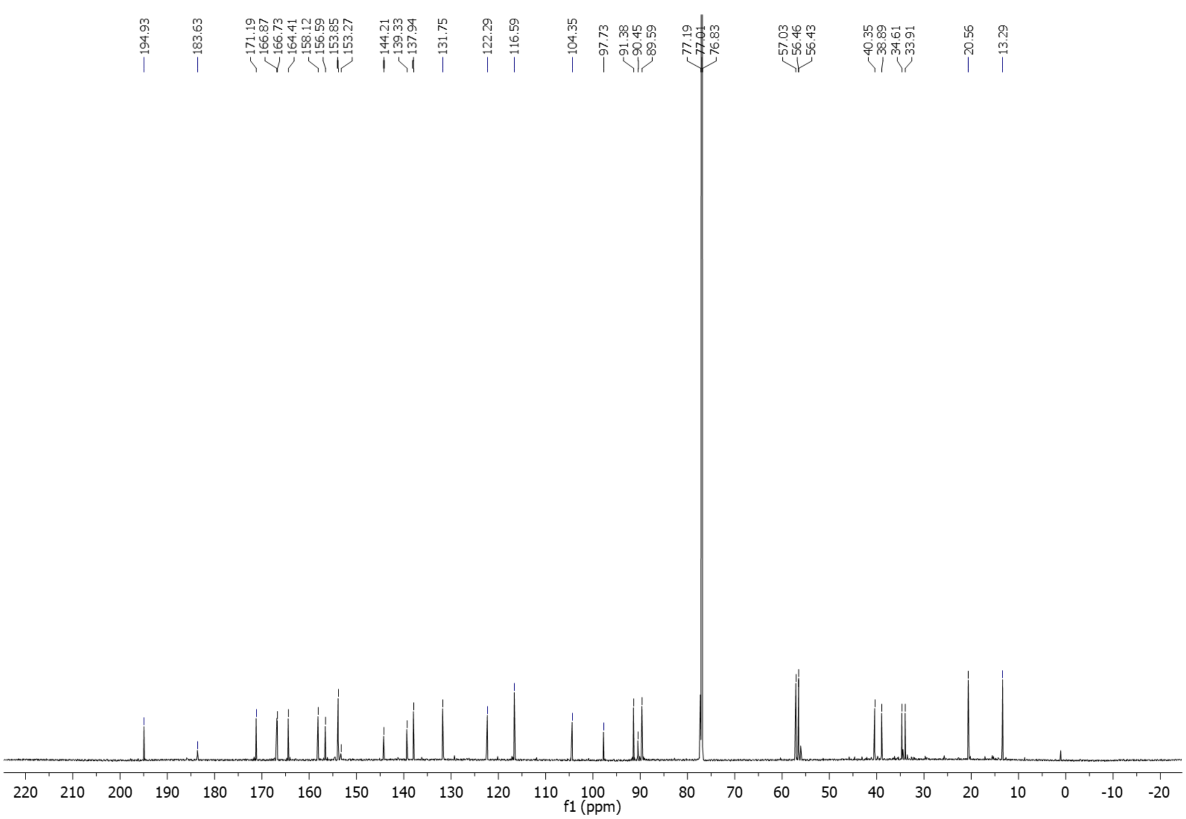

194.93
183.63
171.19
166.87
166.73
164.41
158.12
156.59
153.85
153.27
144.21
139.33
137.94
131.75
122.29
116.59
104.35
97.73
91.38
90.45
89.59
77.19
77.01
76.83
57.03
56.46
56.43
40.35
38.89
34.61
33.91
20.56
13.29
f1 (ppm)

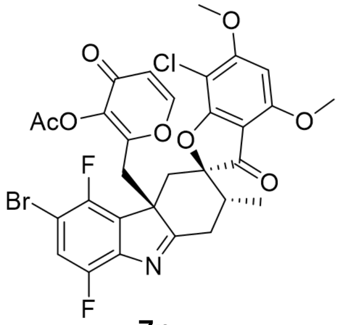
O
Cl
AcO
O
O
O
O
F
Br
N
F

**7n**

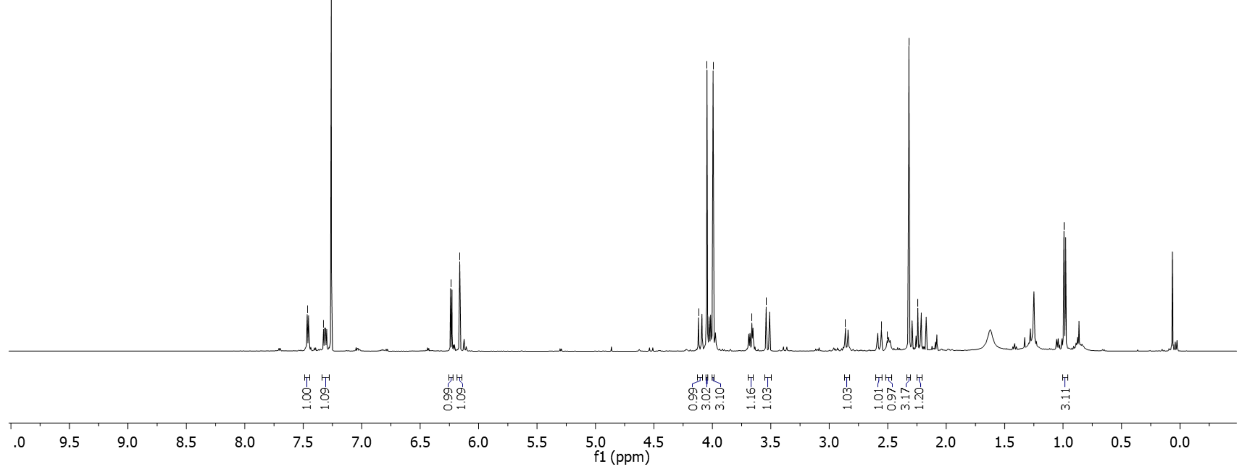
7.47
7.33
6.24
6.16
4.12
4.05
3.99
3.66
3.54
2.87
2.56
2.50
2.32
2.24
0.99
1.00
1.09
0.99
1.09
0.99
3.02
3.10
1.16
1.03
1.03
1.01
0.97
3.17
1.20
3.11
f1 (ppm)

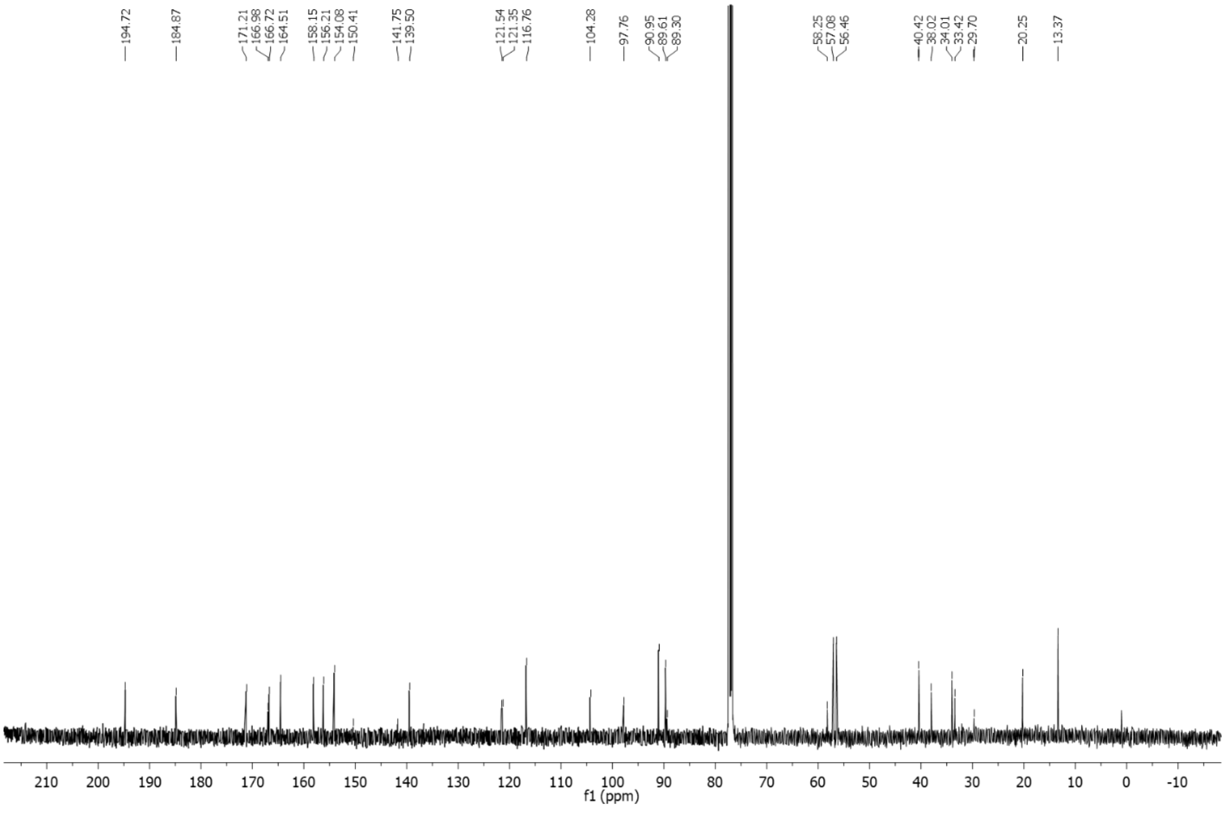
194.72
184.87
171.21
166.98
166.72
164.51
158.15
156.21
154.08
150.41
141.75
139.50
121.54
121.35
116.76
104.28
97.76
90.95
89.61
89.30
58.25
57.08
56.46
40.42
38.02
34.01
33.42
29.70
20.25
13.37
f1 (ppm)

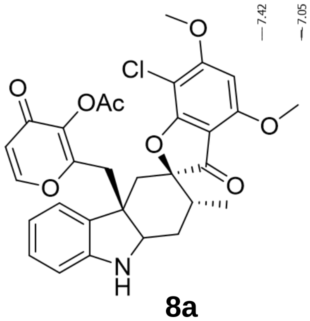
Cl
O
OAc
N
H

**8a**

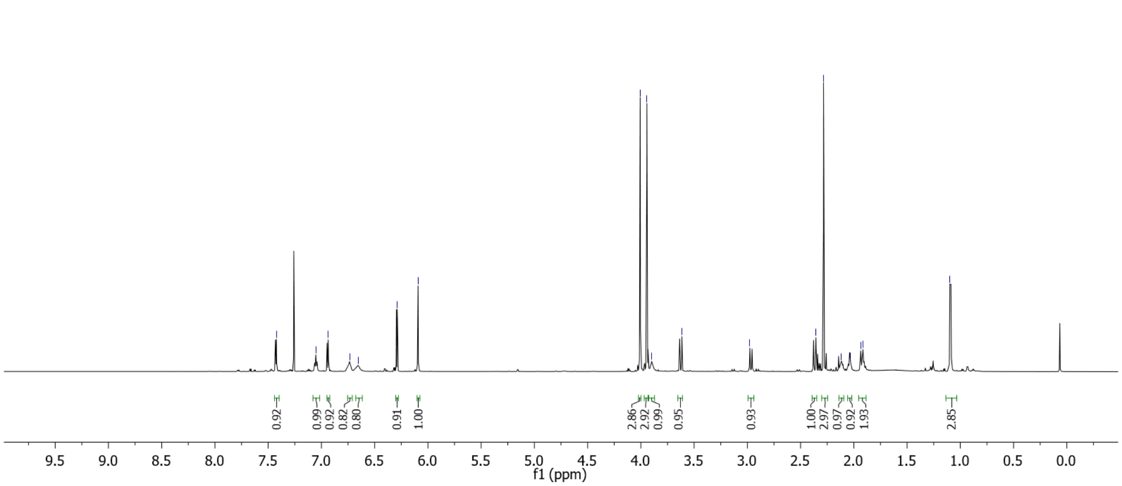
7.42
7.05
6.94
6.74
6.66
6.29
6.09
4.01
3.94
3.90
3.61
2.98
2.36
2.28
2.12
2.03
1.94
1.91
1.10
0.92
0.99
0.92
0.82
0.80
0.91
1.00
2.86
2.92
0.99
0.95
0.93
1.00
2.97
0.97
0.92
1.93
2.85
f1 (ppm)

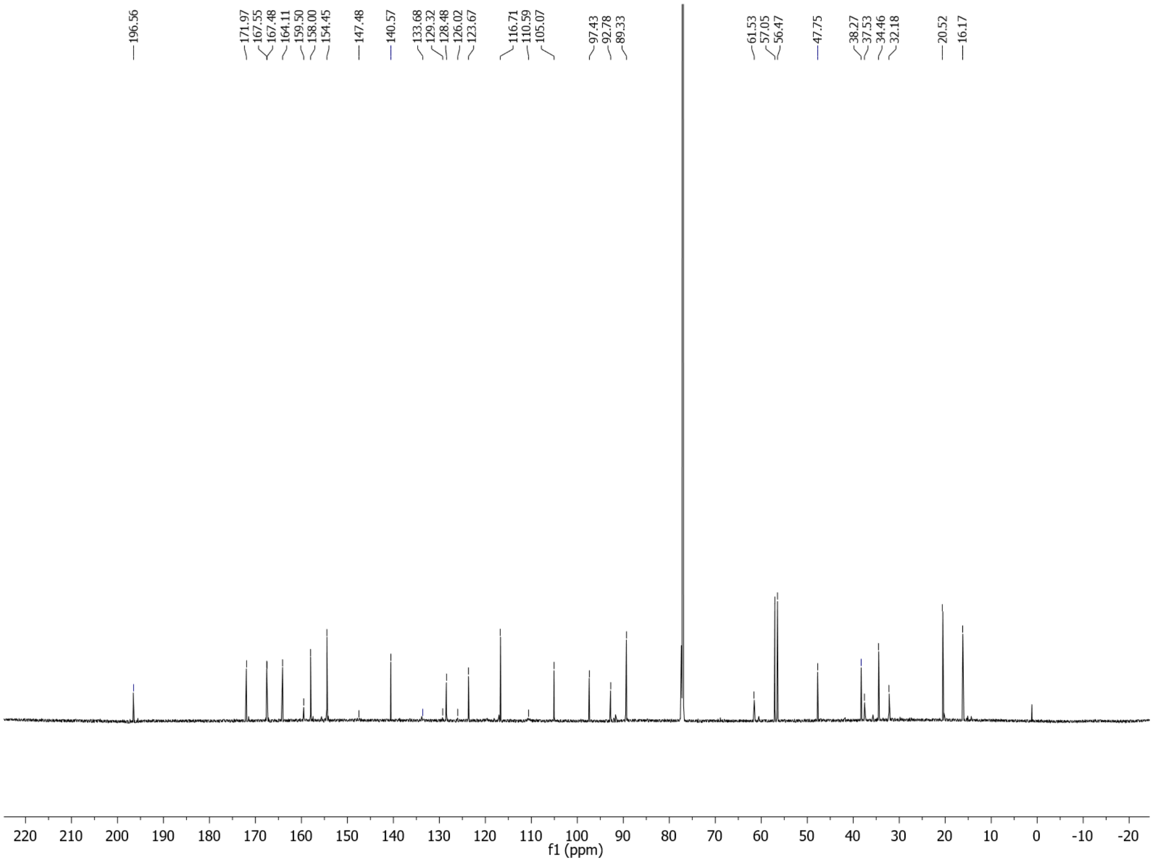
196.56
171.97
167.55
167.48
164.11
159.50
158.00
154.45
147.48
140.57
133.68
129.32
128.48
126.02
123.67
116.71
110.59
105.07
97.43
92.78
89.33
61.53
57.05
56.47
47.75
38.27
37.53
34.46
32.18
20.52
16.17
f1 (ppm)

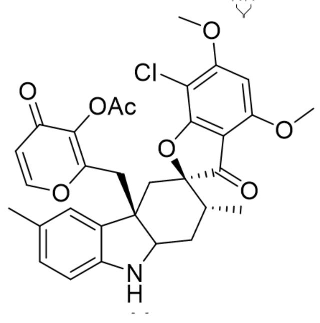

OAc
Cl
N
H


**8b**

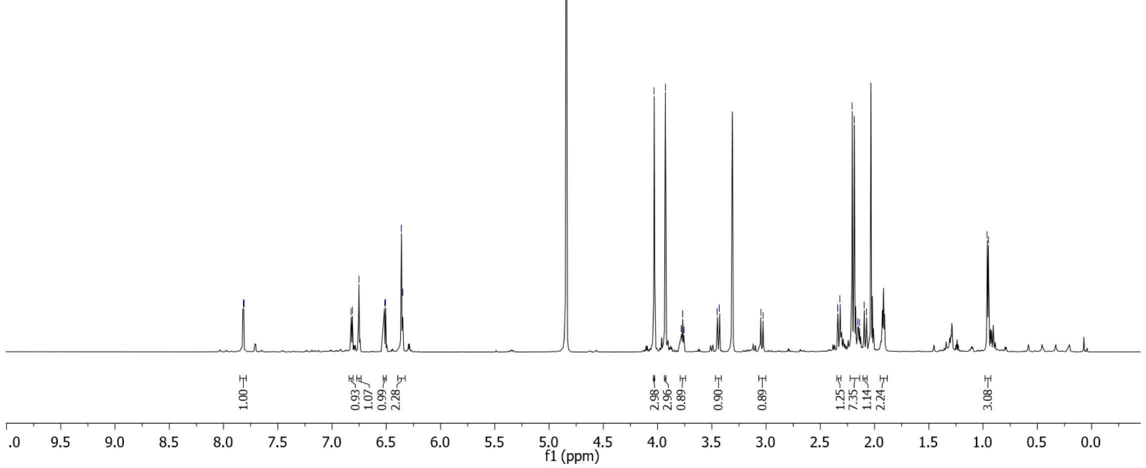

7.82
7.81
6.82
6.81
6.75
6.52
6.51
6.36
6.35
4.03
3.93
3.78
3.77
3.76
3.45
3.43
3.05
3.03
2.34
2.32
2.21
2.19
2.16
2.14
2.09
2.07
0.96
0.95
1.00
0.93
1.07
0.99
2.28
2.98
2.96
0.89
0.90
0.89
1.25
7.35
1.14
2.24
3.08
f1 (ppm)


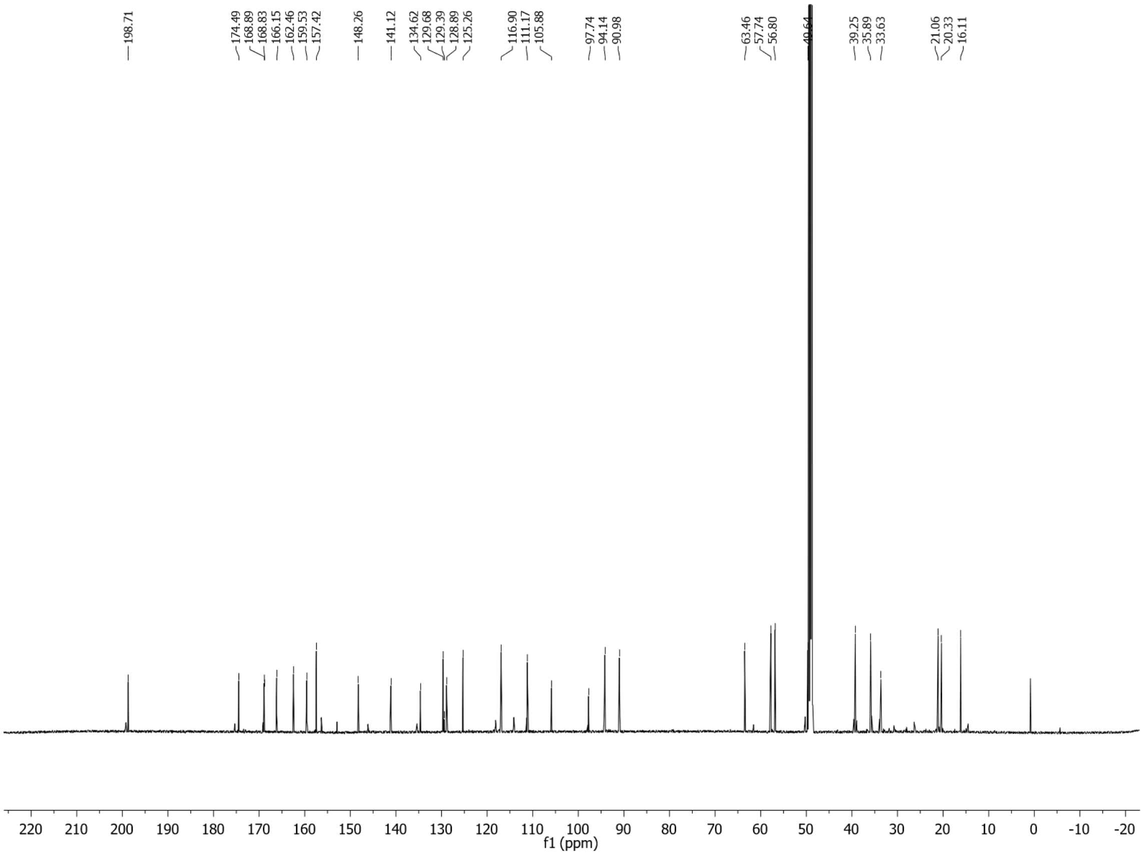

198.71
174.49
168.89
168.83
166.15
162.46
159.53
157.42
148.26
141.12
134.62
129.68
129.39
128.89
125.26
116.90
111.17
105.88
97.74
94.14
90.98
63.46
57.74
56.80
39.25
35.89
33.63
21.06
20.33
16.11
f1 (ppm)

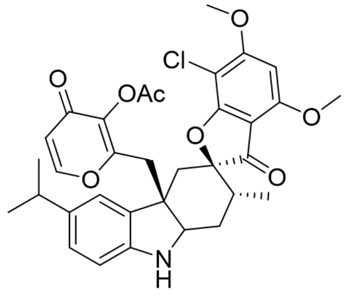
O
OAc
Cl
O
O
O
O
O
N
H

**8d**

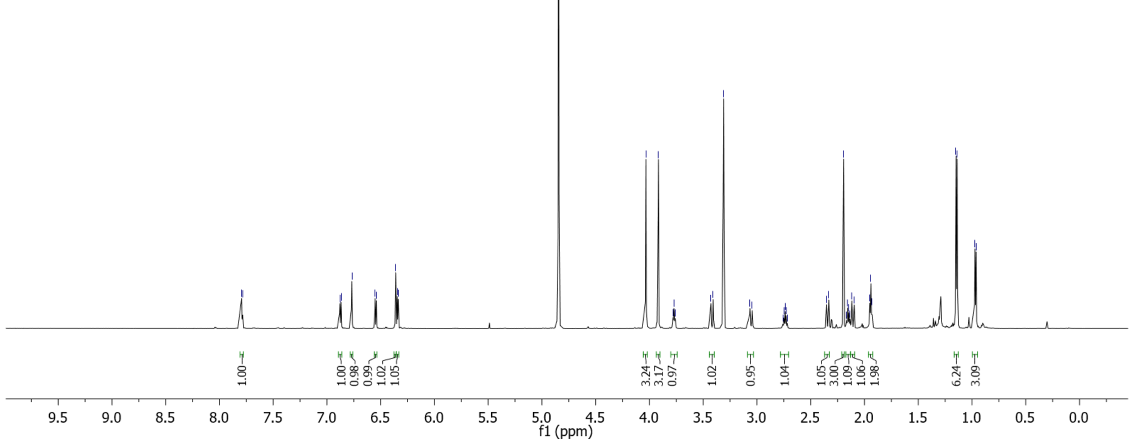
7.79
7.79
6.88
6.87
6.77
6.55
6.54
6.36
6.35
6.34
4.03
3.92
3.78
3.77
3.76
3.43
3.41
3.31
3.06
3.04
2.76
2.75
2.74
2.73
2.72
2.35
2.33
2.19
2.17
2.16
2.15
2.13
2.12
2.10
1.95
1.94
1.93
1.15
1.14
0.97
0.96
1.00
1.00
0.98
0.99
1.02
1.05
3.24
3.17
0.97
1.02
0.95
1.04
1.05
3.00
1.09
1.06
1.98
6.24
3.09
9.5
9.0
8.5
8.0
7.5
7.0
6.5
6.0
5.5
5.0
4.5
4.0
3.5
3.0
2.5
2.0
1.5
1.0
0.5
0.0
f1 (ppm)

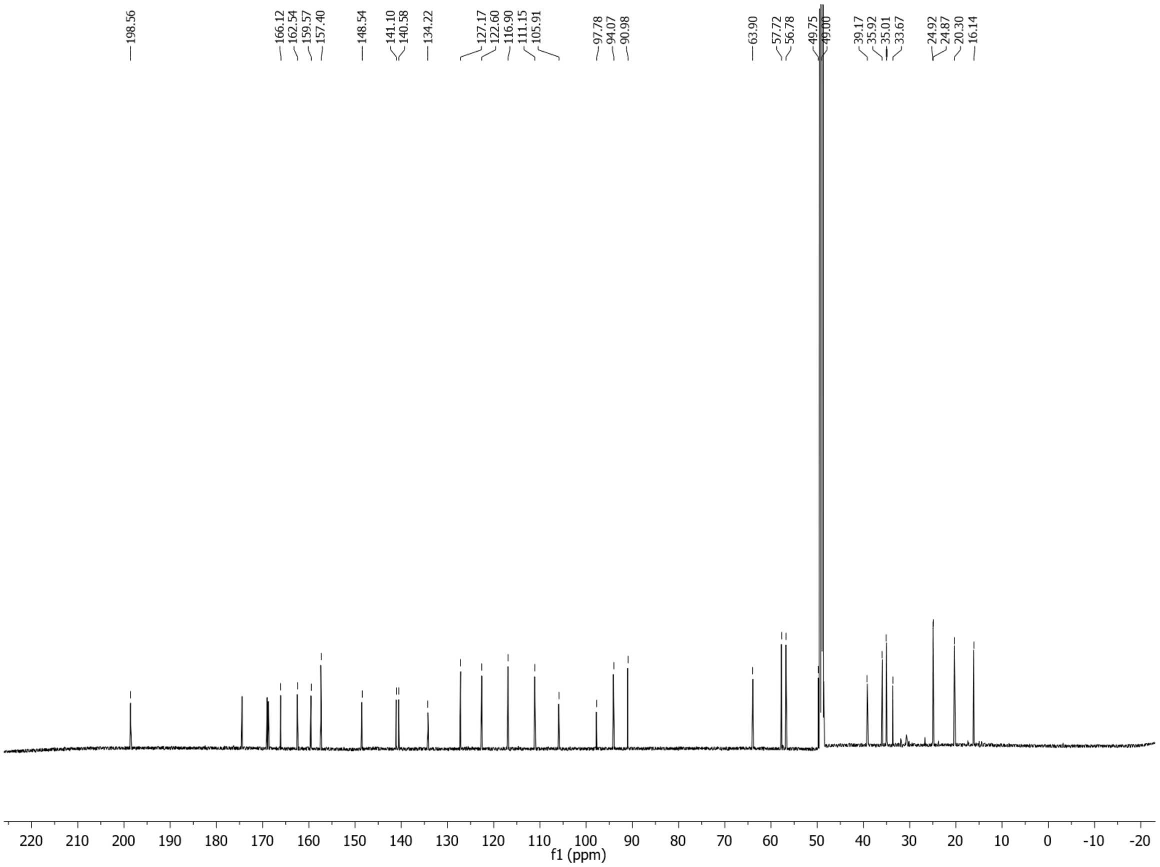
198.56
166.12
162.54
159.57
157.40
148.54
141.10
140.58
134.22
127.17
122.60
116.90
111.15
105.91
97.78
94.07
90.98
63.90
57.72
56.78
49.75
49.00
39.17
35.92
35.01
33.67
24.92
24.87
20.30
16.14
220
210
200
190
180
170
160
150
140
130
120
110
100
90
80
70
60
50
40
30
20
10
0
-10
-20
f1 (ppm)

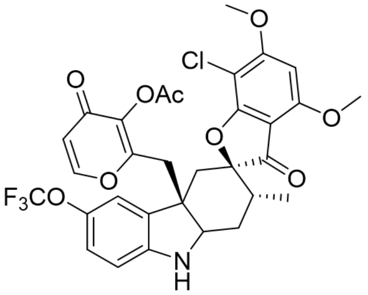


**8i**

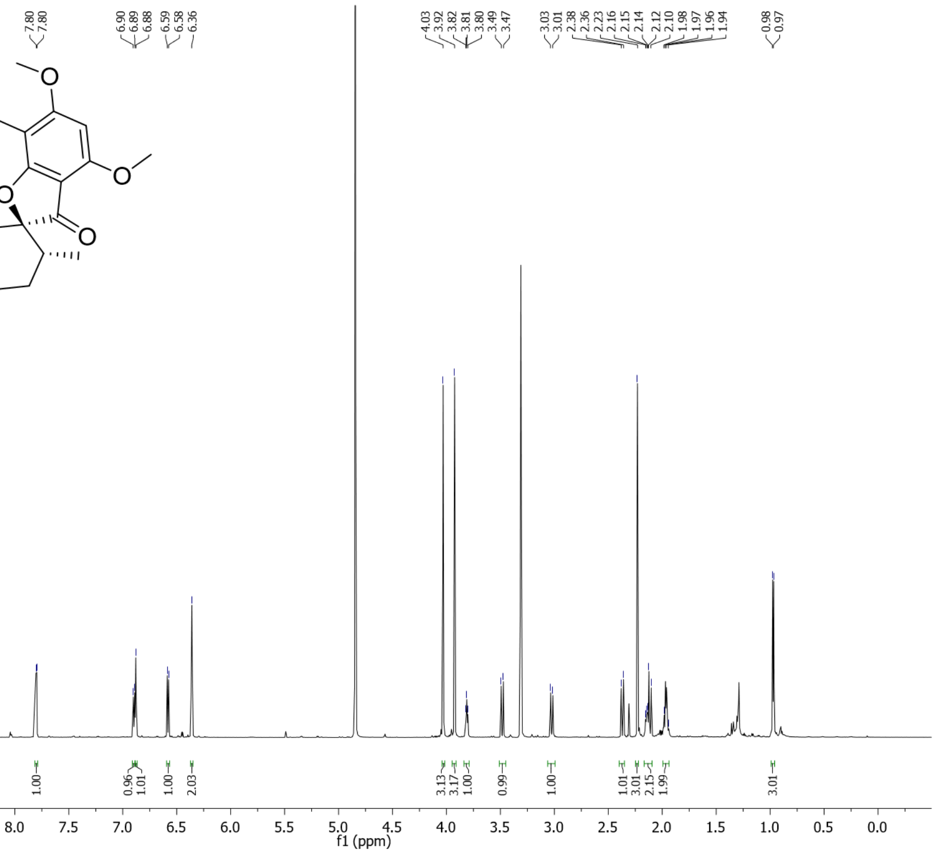

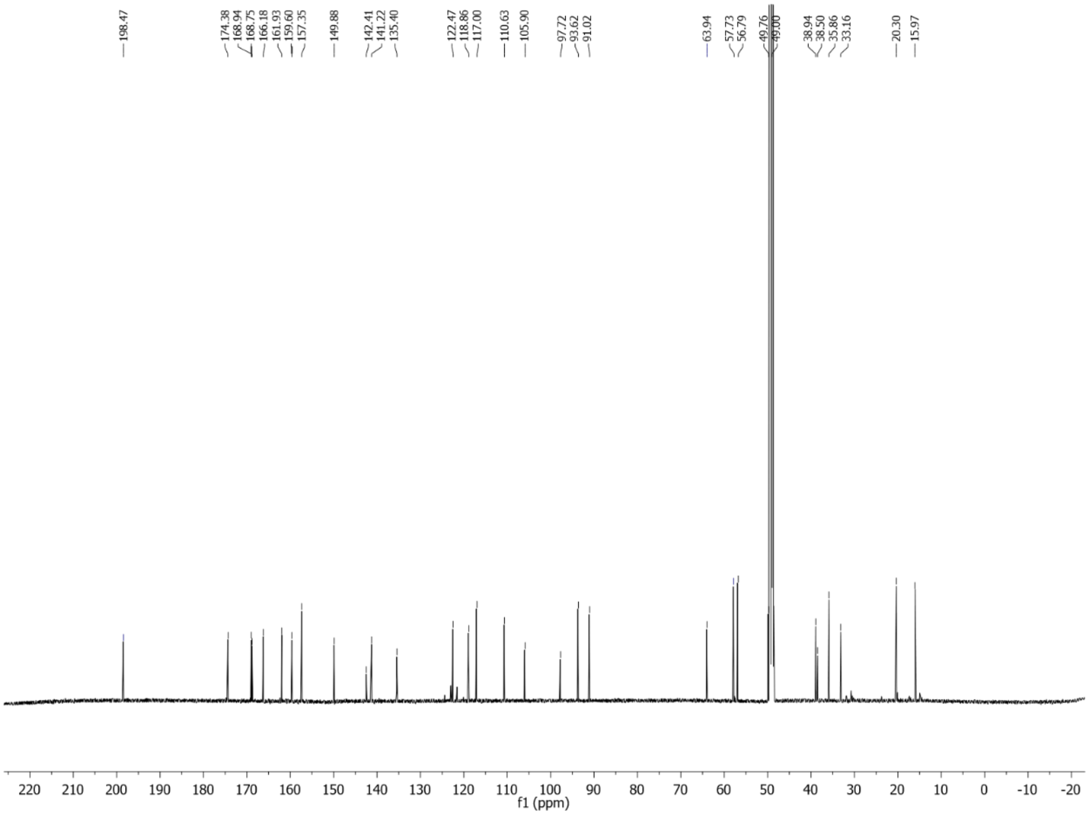

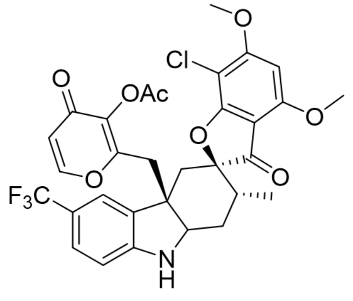
OAc
Cl
O
F$_3$C
N
H

**8j**

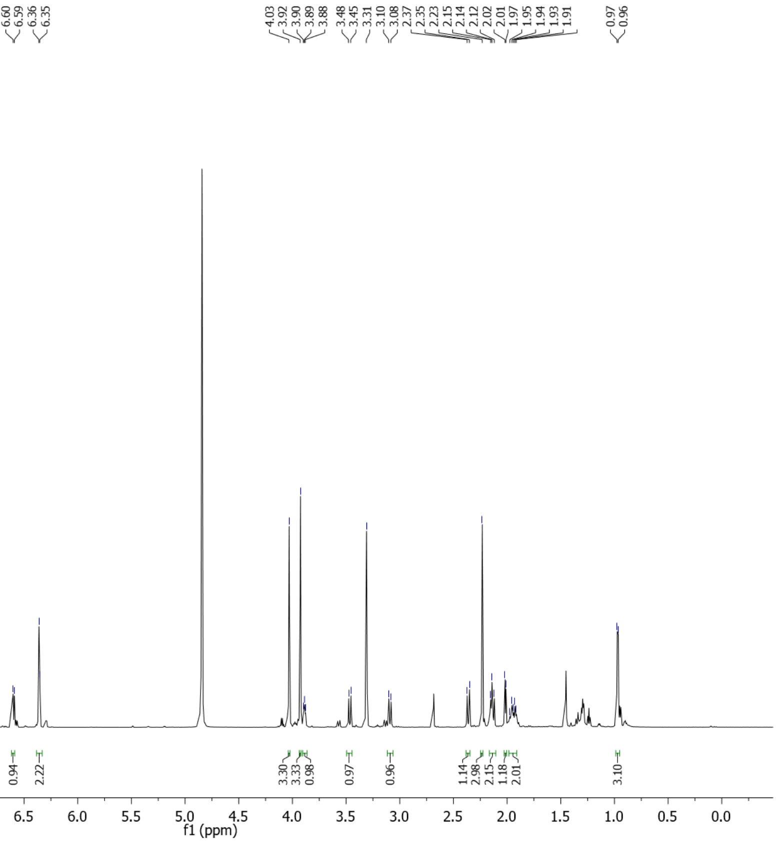
7.81
7.80
7.27
7.26
7.23
6.60
6.59
6.36
6.35
4.03
3.92
3.90
3.89
3.88
3.48
3.45
3.31
3.10
3.08
2.37
2.35
2.23
2.15
2.14
2.12
2.02
2.01
1.97
1.95
1.94
1.93
1.91
0.97
0.96
1.00
0.96
1.15
0.94
2.22
3.30
3.33
0.98
0.97
0.96
1.14
2.98
2.15
1.18
2.01
3.10
9.5 9.0 8.5 8.0 7.5 7.0 6.5 6.0 5.5 5.0 4.5 4.0 3.5 3.0 2.5 2.0 1.5 1.0 0.5 0.0
f1 (ppm)

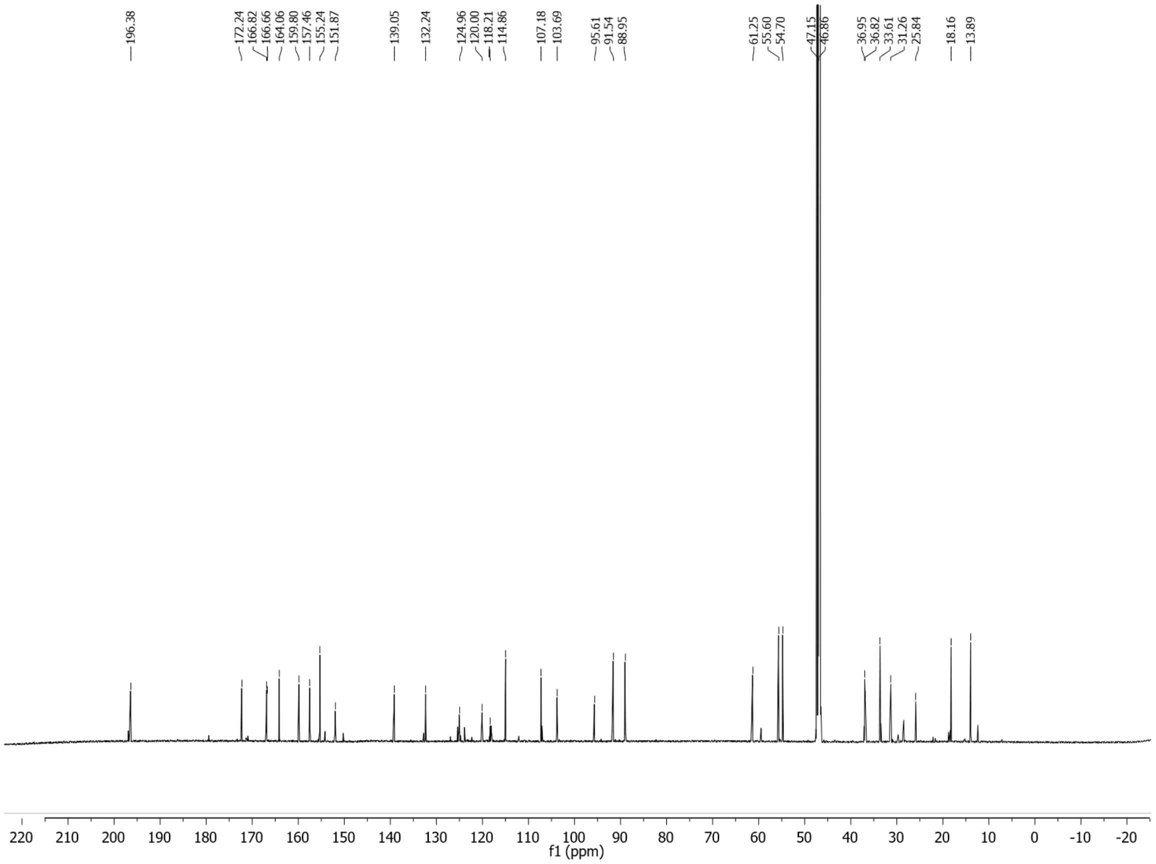
196.38
172.24
166.82
166.66
164.06
159.80
157.46
155.24
151.87
139.05
132.24
124.96
120.00
118.21
114.86
107.18
103.69
95.61
91.54
88.95
61.25
55.60
54.70
47.15
46.86
36.95
36.82
33.61
31.26
25.84
18.16
13.89
220 210 200 190 180 170 160 150 140 130 120 110 100 90 80 70 60 50 40 30 20 10 0 -10 -20
f1 (ppm)

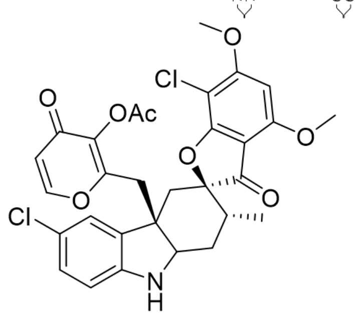
O
OAc
Cl
O
O
O
Cl
O
NH
O

**8l**

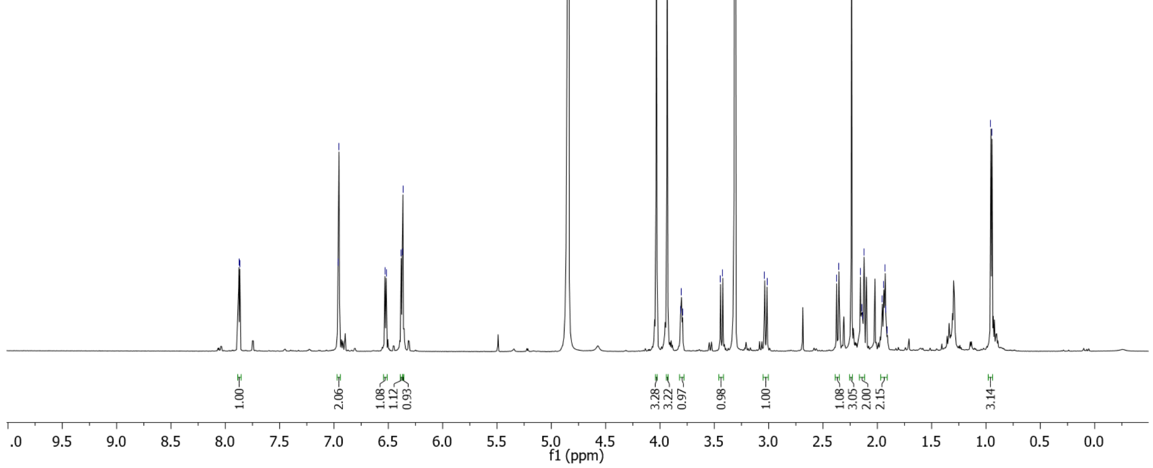
7.87
7.87
6.96
6.95
6.53
6.52
6.38
6.37
6.37
4.03
3.93
3.81
3.80
3.79
3.44
3.42
3.31
3.04
3.02
2.37
2.35
2.24
2.16
2.14
2.13
2.12
1.95
1.94
1.93
1.92
1.91
0.95
0.94
1.00
2.06
1.08
1.12
0.93
3.28
3.22
0.97
0.98
1.00
1.08
3.05
2.00
2.15
3.14
f1 (ppm)

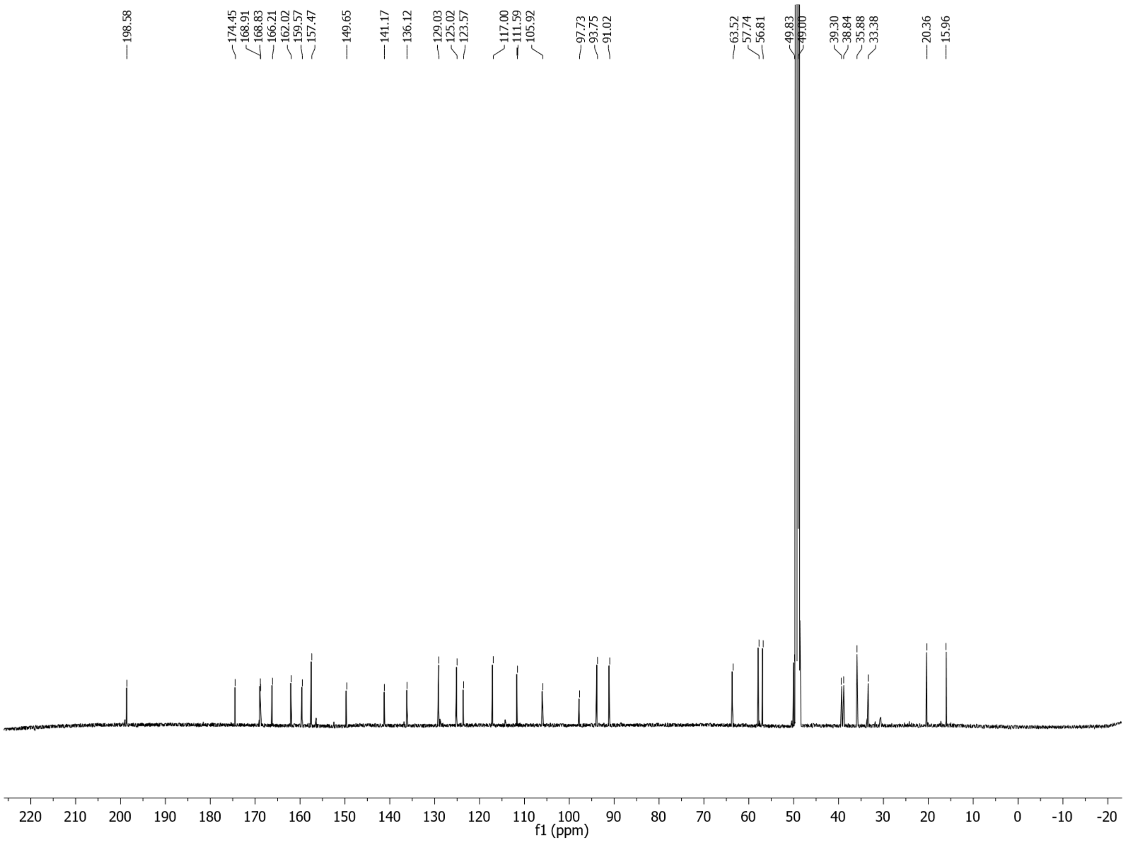
198.58
174.45
168.91
168.83
166.21
162.02
159.57
157.47
149.65
141.17
136.12
129.03
125.02
123.57
117.00
111.59
105.92
97.73
93.75
91.02
63.52
57.74
56.81
49.83
49.00
39.30
38.84
35.88
33.38
20.36
15.96
f1 (ppm)

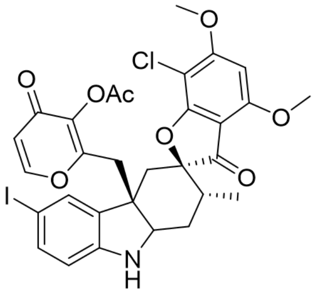
Cl
OAc
O
O
O
O
O
O
I
N
H

**8m**

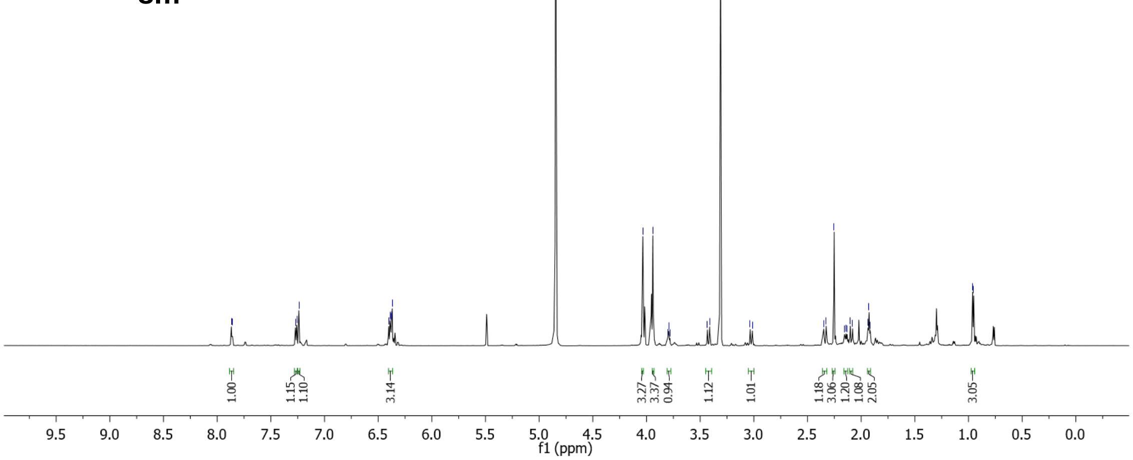
7.87
7.86
7.27
7.26
7.24
6.40
6.39
6.38
6.38
6.37
4.03
3.94
3.80
3.79
3.78
3.43
3.41
3.31
3.03
3.01
2.35
2.33
2.25
2.16
2.14
2.13
2.10
2.08
1.94
1.93
1.92
0.96
0.95
1.00
1.15
1.10
3.14
3.27
3.37
0.94
1.12
1.01
1.18
3.06
1.20
1.08
2.05
3.05
9.5 9.0 8.5 8.0 7.5 7.0 6.5 6.0 5.5 5.0 4.5 4.0 3.5 3.0 2.5 2.0 1.5 1.0 0.5 0.0
f1 (ppm)

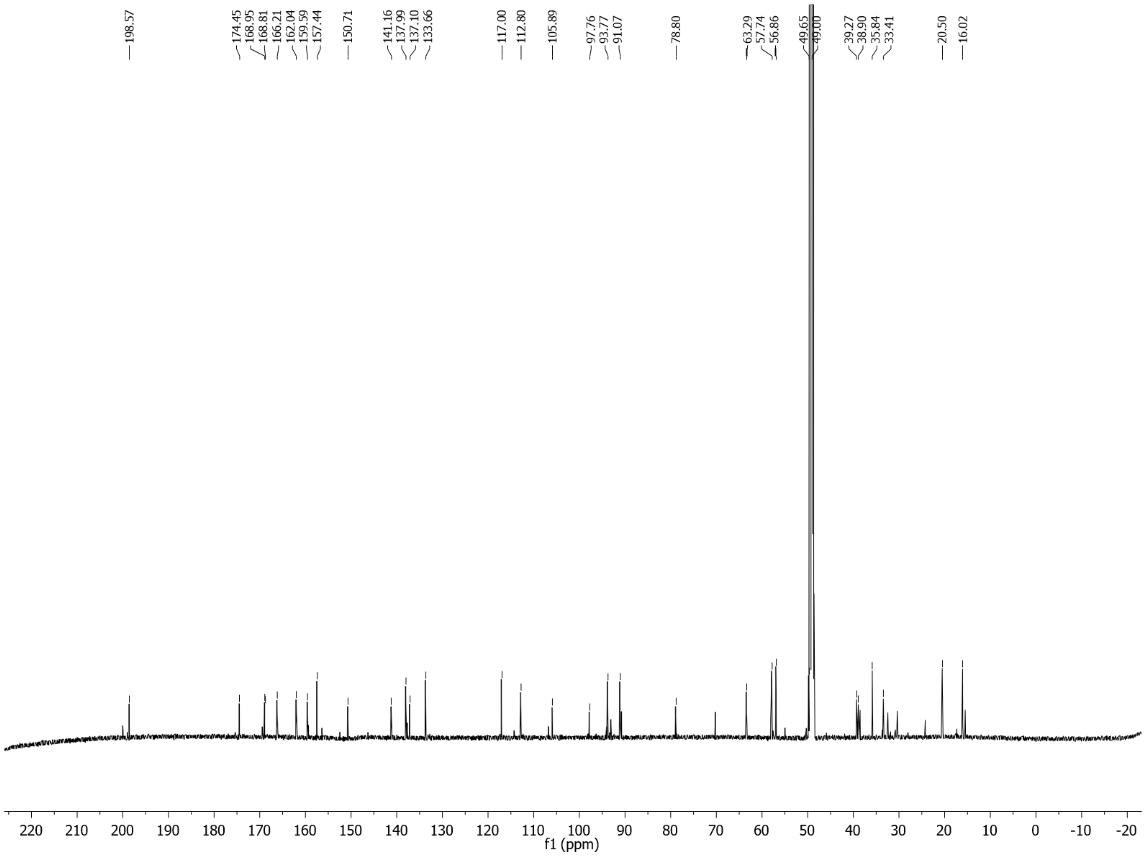
198.57
174.45
168.95
168.81
166.21
162.04
159.59
157.44
150.71
141.16
137.99
137.10
133.66
117.00
112.80
105.89
97.76
93.77
91.07
78.80
63.29
57.74
56.86
49.65
49.00
39.27
38.90
35.84
33.41
20.50
16.02
220 210 200 190 180 170 160 150 140 130 120 110 100 90 80 70 60 50 40 30 20 10 0 -10 -20
f1 (ppm)
